# Visualizing and diagnosing spillover within randomized concurrent controlled trials through the application of diagnostic test assessment methods

**DOI:** 10.1186/s12874-024-02296-1

**Published:** 2024-08-16

**Authors:** James C. Hurley

**Affiliations:** 1 Melbourne Medical School, University of Melbourne, Ballarat, Australia; 2 Internal Medicine Service, Ballarat Health Services, Grampians Health, PO Box 577, Ballarat, 3353 Australia; 3 Ballarat Clinical School, Deakin University, Ballarat, Australia

**Keywords:** Spillover, Infection prevention, Intensive care, Diagnostic test assessment, Randomized concurrent controlled trials, Heterogeneity, SROC plots, Arms-based, Contrast-based, Caterpillar plots

## Abstract

**Background:**

Spillover of effect, whether positive or negative, from intervention to control group patients invalidates the Stable Unit Treatment Variable Assumption (SUTVA). SUTVA is critical to valid causal inference from randomized concurrent controlled trials (RCCT). Spillover of infection prevention is an important population level effect mediating herd immunity. This herd effect, being additional to any individual level effect, is subsumed within the overall effect size (ES) estimate derived by contrast-based techniques from RCCT’s. This herd effect would manifest only as increased dispersion among the control group infection incidence rates above background.

**Methods and results:**

The objective here is to explore aspects of spillover and how this might be visualized and diagnosed. I use, for illustration, data from 190 RCCT’s abstracted in 13 Cochrane reviews of various antimicrobial versus non-antimicrobial based interventions to prevent pneumonia in ICU patients. Spillover has long been postulated in this context. Arm-based techniques enable three approaches to identify increased dispersion, not available from contrast-based techniques, which enable the diagnosis of spillover within antimicrobial versus non-antimicrobial based infection prevention RCCT’s. These three approaches are benchmarking the pneumonia incidence rates versus a clinically relevant range, comparing the dispersion in pneumonia incidence among the control versus the intervention groups and thirdly, visualizing the incidence dispersion within summary receiver operator characteristic (SROC) plots. By these criteria there is harmful spillover effects to concurrent control group patients.

**Conclusions:**

Arm-based versus contrast-based techniques lead to contrary inferences from the aggregated RCCT’s of antimicrobial based interventions despite similar summary ES estimates. Moreover, the inferred relationship between underlying control group risk and ES is ‘flipped’.

**Supplementary Information:**

The online version contains supplementary material available at 10.1186/s12874-024-02296-1.

## What is new?

### What is already known on this topic

- Spillover of effect from intervention to concurrent control group patients invalidates the Stable Unit Treatment Variable Assumption (SUTVA) fundamental to valid inferences from randomized concurrent controlled trials (RCCT).
- Diagnostic test assessment (DTA) methods use an arms-based framework of meta-analysis versus the contrast-based framework traditionally applied to the meta-analysis of RCCT’s.
- In the ICU population, colonization, which underlies infections such as pneumonia, is contagious. The occurrence of spillover was postulated in the earliest study of an antimicrobial based pneumonia prevention in an ICU population.
- How much spillover effect, whether positive or negative, that originates from antimicrobial interventions to prevent pneumonia in ICU populations remains unaddressed in > 50 meta-analyses and systematic reviews, including four Cochrane reviews of the topic.

### Key findings

- The range and dispersion of pneumonia incidence among the RCCT component (control and intervention) groups within ICU acquired pneumonia prevention RCCT’s within 13 Cochrane reviews, are best displayed within SROC plots as used in DTA.
- These SROC’s display unusual dispersion patterns in the pneumonia incidence among the control groups of RCCT’s of antimicrobial based interventions.

## What are the implications?

Diagnosing spillover among the RCCT’s of infection prevention interventions within systematic reviews requires visualizing dispersion with which to appraise SUTVA. These are enabled only within arms-based techniques and not within contrast-based techniques.

## Background

Spillover of intervention effect, by influencing the event rate among concurrent control groups, threatens the Stable Unit Treatment Variable Assumption (SUTVA) [1]. This assumption is required for valid inference from the effect size (ES) estimates from randomized concurrent controlled trials (RCCT) and, by flow on, from the summary ES estimates derived within systematic reviews using the traditional contrast-based framework [2]. SUTVA, if true, permits a valid causal inference from the ES derived from RCCT’s. If false, an RCCT derived ES estimate is not easily interpretable [3]. In which case other study designs such as a cluster randomized trial (CRT), would be required [4]. Of note, there being no simple test of SUTVA, RCCT’s commonly either assume it to be valid or fail to mention SUTVA.

## Outline

The objectives here are to demonstrate how an arms-based analysis enables a visualization and possible diagnosis of spillover among RCCT’s and why this is not possible from a traditional contrast-based analysis. Three aspects are discussed below; the nature of spillover, how spillover will not be identifiable using conventional contrast based methods and the novel application of diagnostic test assessment (DTA) methods as an arms based method together with measures of dispersion to enable its recognition. The tutorial uses data from 190 RCCT’s abstracted in 13 Cochrane reviews of various antimicrobial versus non-antimicrobial based interventions to prevent pneumonia in ICU patients as an example for diagnosing spillover. Spillover has long been postulated for antimicrobial interventions in this context but never formally evaluated.

### Spillover and infection prevention interventions

Spillover is an indirect effect mediated by contagion occurring within populations originating from those who receive an intervention of interest to impact those individuals that do not [5–8]. Spillover is important to consider in estimating the population level effects of infection prevention interventions such as vaccination programs against contagious infections such as COVID, cholera, typhoid, and influenza [6, 7]. In these examples, spillover mediates herd protection though lowering the infection rate in both those recipients of the intervention and, indirectly, those non-recipients concurrent within the same population. Moreover, in evaluating the population level effects of vaccination interventions, the causal inference (efficacy) for individuals is not of primary interest whereas the population effectiveness is [5, 6].

**Fig. 1:**
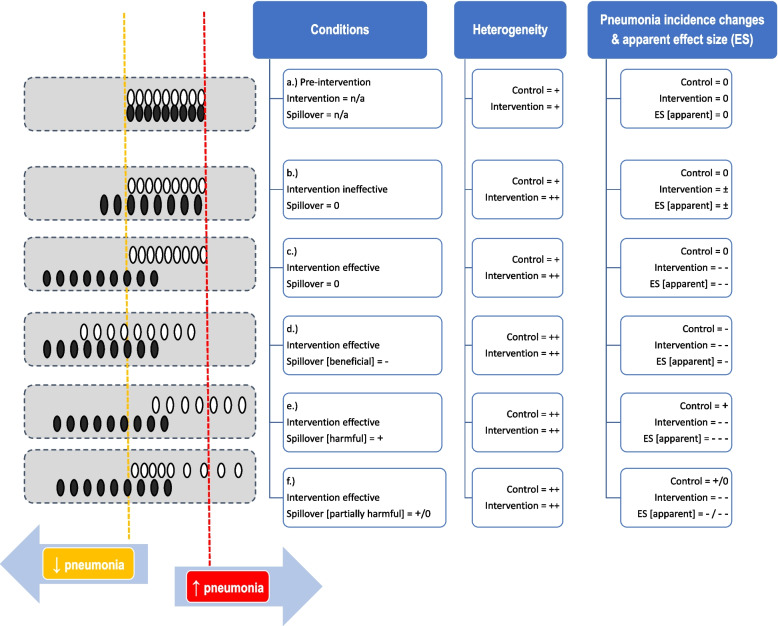
Schema of six conditions of spillover ('a'. - 'f'.) contributing to heterogeneity in pneumonia incidence proportions among component groups of ICU patients within RCCT’s contained within a systematic review in relation to a clinically relevant incidence range (dotted lines) within this population. Note that pneumonia in the ICU context arises from colonization which is contagious within the ICU context. Movement to the right and left represents increasing or decreasing pneumonia incidence above or below the upper or lower end of the clinically relevant range (dotted red or yellow lines, respectively). The dotted rectangles at left represent systematic reviews reporting data for control (○) and intervention (●) groups of RCCT’s. The conditions (‘a’ – ‘f’) provide exposure to interventions which might be effective (–) or ineffective ( ±) at preventing pneumonia for individuals within intervention groups. Any intervention or spillover effect will contribute to heterogeneity at both the level of the group and the study ES. At the ‘herd’ (group) level there is either no spillover (0) or spillover which is beneficial (-) or harmful ( +) towards pneumonia incidence for individuals within control groups. The nett result is the apparent ES reported as the summary ES in each RCCT and systematic review. Note that ‘-’ equates to prevention (i.e. reduction) in pneumonia and ‘ + ‘ is the converse. a. Unexposed (Pre-exposure) component groups to intervention (potential outcomes not yet observed). b. Ineffective intervention and no spillover. c. Effective intervention and no spillover. d. Effective intervention and spillover which is beneficial (reducing pneumonia). e. Effective intervention and spillover which is harmful (increasing pneumonia). f. Effective intervention and spillover which is harmful (increasing pneumonia) but is uneven being present among some RCCT’s and not others

Ideally, RCCT’s enable the estimation of an intervention ES by comparing the event rates in concurrent control and intervention groups. In conducting an RCCT of an infection prevention intervention any spillover will influence the event rate within the concurrent control groups of the RCCT’s although the size of the spillover effect will vary between RCCT’s. Hence spillover will likely amplify any inherent dispersion of the event rate among concurrent control groups depending on the strength of this indirect effect. By contrast, the dispersion among the corresponding intervention groups will mostly reflect the heterogeneity in the ES of the various infection prevention interventions under study within different RCCT’s in addition to the inherent dispersion in the incidence rate (Fig. 1). Hence, the overall ES estimate from an RCCT incorporates both the direct effect of the intervention on the intervention group individuals plus any indirect spillover effect, whether positive or negative, on the control group individuals.

Diagnosing spillover, being a population (i.e. herd) level effect manifest on individuals, will require the following conditions; a defined end point of interest, clusters of populations of interest, an intervention of interest with spillover potential, and the exposure, or not, of these multiple comparable defined populations (i.e. exchangeable herds) to the intervention with incomplete penetrance. An example of where these conditions have been met is the inference of spillover on typhoid incidence among individuals within eighty Kolkata neighbourhoods cluster randomized to receive exposure, or not, to a population typhoid vaccination program delivered with incomplete penetrance to individual residents within the neighbourhoods [8].

Identifying spillover will require methods to quantify the amount and direction of increased dispersion in the event rate among the non-recipients within these populations to one of three comparators. Firstly, this could be relative to background dispersion such as that among the non-recipients within herds exposed to an intervention ineffective against the end point of interest. In the typhoid example, the ineffective intervention was neighbourhood exposure to a hepatitis vaccination program [8]. Second, this could be relative to the dispersion among the recipients of the effective intervention. Thirdly, this could be relative to a clinically relevant incidence range for the end point of interest for the population of interest, where this is available.

### DTA and the arms-based framework

Clinical studies of diagnostic tests differ fundamentally from RCCT’s in that the study sub-populations, those with versus without the disease of interest, have not been defined by random allocation [9]. Also, the diagnostic test threshold typically varies across studies to accommodate ‘‘rule in’’ versus ‘‘rule out’’ testing strategies [10–12]. The SUTVA is generally neither valid nor a relevant consideration in relation to DTA. Hence DTA meta-analyses are undertaken within an arms-based framework with the test performance characteristics reported as summary sensitivities and specificities. Whilst a summary diagnostic odds ratio (DOR) might be available, this is not generally of interest except when comparing the results for different diagnostic tests or applications in different populations. Whereas the aggregation of results from high quality RCCT studies to achieve a more precise causal effect estimate is usually a realistic and desirable goal (in the absence of spillover), this is not the case for DTA.

For DTA, the primary interest is the projection to future applications of the test. To achieve this objective, current DTA methods provide three outputs not usually of interest within a contrast-based synthesis [13–15]. Firstly, the study level and summary sensitivities and specificities are often provided together with associated 95% confidence intervals. Second, DTA methods provide the summary receiver operator characteristic (SROC) plot, which displays both the dispersion in the sensitivities and specificities and how they co-vary across the aggregated studies. The visual representation of the SROC plot summary has evolved over time from a summary point (Q*, where summary sensitivity = 1 minus specificity), the SROC curve and, most recently, as a 95% confidence ellipse [9]. Thirdly, the dispersion of sensitivity and specificity are visualized in the SROC as a 95% prediction ellipse. These outputs are of great interest towards projecting future utility of the diagnostic test to applications in comparable populations.

### Parallels between the SROC and L’abbe plots

The SROC derived within a DTA resembles the L’abbe plot as derived within a meta-analysis of RCCT’s. Each displays the dispersion in event rates in the two component groups, along the y-axis for one versus the x-axis for the other [16, 17]. For the L’abbe plot, these are the event rates in the intervention versus control groups, respectively. For the SROC plot, these are the test positive rates among the diseased (sensitivity) versus the non-diseased (which equates to 1 minus specificity), respectively. In both cases, the diagonal (y = x line) represents the locus where the event rates in the two populations in the comparison are equal. The two plots differ in how the covariation away from this line is displayed and how event rate dispersion is inferred. For the L’abbe plot, depending on whether the ES is defined as an odds ratio (OR), a risk ratio (RR) or a risk difference (RD) giving a visual representation of covariation as variously a line parallel to the y = x line (RD), a line that passes through the origin (RR), or a curve (OR), respectively. For the L’abbe plot, dispersion is assessed merely as a subjective visual impression which is governed by whether the presumptive underlying relationship is a RD, RR or OR.

For the SROC plot, on the other hand, the underlying relationship is always as an OR and the dispersion in event rates, being quantified as a summary point together with the derivation of an enveloping 95% prediction ellipse, enables projections of the sensitivity and specificity to future applications of the diagnostic test.

The most recent DTA methods require logistic transformation of sensitivity and specificity with the covariation defined within either bivariate or multi-level random effects models [18–20]. On logistic transformation, the SROC relationship has a linear (straight line) regression which, on back transformation to the linear scale, becomes curved. The SROC displays the summary operating point, which map the summary values of sensitivity and specificity along the SROC curve within the plot. Moreover, these models provide bi-directional 95% confidence regions (as ellipses) rather than as two unidirectional 95% confidence limits together with 95% prediction ellipses. On back transformation to the linear scale, these 95% ellipse regions lose their elliptical shape.

### Indicators of dispersion

Dispersion of ES estimates within the contrast-based framework are of interest towards understanding the stability of the ES estimate. Commonly calculated measures are tau^2^, I^2^, and H2 although they are each imperfect measures which are widely mis-interpreted [21]. For example, I^2^, and H2 merely provide the ratio between the proportion of observed variance that might be due to variation in true effects versus sampling error [22]. The 95% prediction limits, although less commonly reported, are considered a better representation of the potential dispersion of the ES estimate. That there is > 200 types of graphical displays that are available for meta-analysis and systematic reviews in part reflects that in conducting a meta-analysis, dispersion is best appreciated when visualized [16, 17].

A key role for graphical displays of dispersion, within both the contrast-based and the arms-based framework, is its application towards identifying the balance between potential outlier versus inlier study results towards the summary effect. The L’abbe plot is not optimal in this role compared to other methods [23]. Another method for achieving this visually is within a caterpillar plot which is a forest plot with the studies ordered by increasing study specific incidence of ES [24]. However, caterpillar plots are infrequently used because their interpretation is limited if there are insufficient studies. Additionally, within the arms-based framework, there is the potential to reference either a clinically relevant range, where this is available either from expert opinion or independent sources, or a range that is considered meaningful [25].

The above commentary does not consider the application of contrast-based versus arms-based analysis within network meta-analysis. This is an active area of research beyond what is considered here in the diagnosis of spillover on concurrent control groups within infection prevention RCCT’s [26].

### Illustrative example

#### Pneumonia prevention among ICU patients

Patients receiving mechanical ventilation are at high risk of acquiring pneumonia (Ventilator associated pneumonia; VAP) whilst in the intensive care unit (ICU) [27–30]. An extensive range of methods, being either non-antimicrobial [31–39] or antimicrobial [40–43] based, have been studied among patients receiving, or likely to receive, mechanical ventilation towards preventing VAP. Many of the interventions studied in these RCCT’s are included within national programs aiming for “pneumonia zero” [30]. Of note, the pneumonia incidence in the ICU population is considered by experts to lie within 5 and 40% [28] or as a more conservative range 8 to 28% [29]. Length of ICU stay is a strong correlate [27].

These RCCT’s have been summarized within Cochrane reviews [31–43]. The summary ES derived within these Cochrane reviews estimate pneumonia incidence reductions of > 50% using antimicrobial based interventions [40–43], versus non-antimicrobial based interventions [31–39] which achieve more modest or no significant reductions.

Antimicrobial based interventions, using either topical antiseptics and oral care [40, 41] or antibiotics [42, 43], were presumed to alter the microbiome of the entire ICU. This spillover of intervention effect was anticipated from the first study [44] being postulated as “….*having heavily contaminated patients next to decontaminated patients might adversely affect the potentially beneficial results* [postulate one]. *Secondly, a reduction of the number of contagious patients by applying [selective digestive decontamination] SDD in half of them, might reduce the acquisition, colonisation and infection incidence in the not-SDD-treated control group* [postulate two].” [44].

Whilst antimicrobial interventions are believed to mediate prevention by altering the ICU microbiome [45–47], neither the size nor the direction of spillover has ever been estimated despite > 60 RCT’s and > 50 systematic reviews and meta-analyses of antimicrobial based interventions. The original presumption that the spillover from antimicrobial based interventions, as for the herd effects of vaccination interventions, would always be beneficial has never been proven [44]. By contrast, any spillover for non-antimicrobial interventions will likely be minimal, because they are relatively ineffective at preventing pneumonia and also because they have minimal impact on the ICU microbiome.

This tutorial uses the data from 190 RCCT’s abstracted in 13 Cochrane reviews of non-antimicrobial [31–39] and antimicrobial based [40–43] interventions to prevent pneumonia in ICU patients receiving or likely to receive, mechanical ventilation. This collection of studies has been analysed elsewhere [48] where additional details together with both an arms-based and a traditional contrast-based analysis of the data is available.

#### Pneumonia prevention among ICU patients: the data and the interventions

The non-antimicrobial category includes upper gastro-intestinal tract (UGIT) [31], feeding [32–34], airway [35–38], and probiotic [39] based interventions. The antimicrobial category includes topical antiseptic or oral care [40, 41], and topical antibiotic [42, 43] based interventions.

For some antimicrobial RCCT’s the control group patients received a protocolized antimicrobial intervention in addition to standard care. These RCCT’s, here termed antimicrobial duplex studies, are separately classified in the Cochrane reviews [40–43] and here constitute a third category.

All data analyzed are provided in the supplemental material. The data is arrayed in a layout as for the analysis of a diagnostic test with the count of patients with pneumonia and the count without pneumonia for the intervention and control groups, respectively. The Stata commands are listed in the supplement.

#### Contrast-based analysis

For the contrast-based analysis, the meta-analysis models of prevention ES with associated estimates of heterogeneity were undertaken using mixed-effect methods of meta-analysis using the ‘meta’ and ‘meta meregress’ command in Stata 18 (Stata Corp., College Station, TX, USA) [49].

#### Arms-based analysis

For the arms-based analysis, the pneumonia count data was analysed as if for a diagnostic test with the counts in the intervention and control groups representing the disease positive and negative groups, respectively. The analysis was conducted as if for a DTA using the ‘metandi’ user command to generate summary measures of ‘sensitivity’ and ‘1 minus specificity’ (pneumonia incidences in the intervention and control groups, respectively) [13]. The SROC plots were generated with the ‘metandiplot’ command [13]. SROC plots generated using the more recently developed ‘metadta’ command [14] are also displayed for comparison.

#### Diagnostic approaches

The diagnosis of spillover requires the identification of increased dispersion in event rate, whether assessed visually, within SROC plots, or by using heterogeneity metrics, among control groups of RCCT’s within these three categories. There are three approaches to assessing this dispersion.

- by comparison to the dispersion among the corresponding intervention groups receiving the antimicrobial intervention,
- by comparison to the dispersion among the control groups within RCCT’s of an ineffective intervention, which here is the non-antimicrobial based RCCT’s,
- by comparison to the clinically relevant pneumonia incidence benchmark range [28, 29].

All three approaches are used here.

The principal analysis examines the three broad categories of intervention. A secondary level of analysis, located in the supplement, explores the intervention subcategories corresponding to listings within individual Cochrane reviews [30–43].

#### Simulation studies

To explore the utility of DTA methods for visualizing spillover, I conducted simulation studies based on the non-antimicrobial studies. The RCCT’s of non-antimicrobial interventions can be expected to have spillover between control and intervention groups at a level that would be no greater than that occurring in the ICU context under standard operating conditions.

To simulate negative spillover, the control group pneumonia count was decreased by 2.5 or 5 per 100 control group patients. This equates to the conditions of Fig. 1d.

Positive spillover was simulated under conditions of uniform (Fig. 1e) or partial (Fig. 1f) spillover across RCCT’s. To simulate uniform positive spillover, the control group pneumonia count was increased by 2.5, 5, or 10 per 100 control group patients. Spillover that was positive and partial was simulated by increasing the control group pneumonia count by 10 or 20 per 100 control group patients in half or a quarter of randomly selected control groups.

The outcomes of the simulations were assessed using the SROC plots and the metrics of heterogeneity associated with the control groups.

## Results

### Characteristics of the studies

There were 317 studies listed in 13 Cochrane reviews [31–43] of which 127 studies, either being duplicate or without VAP data or with < 50% of patients receiving MV, were excluded leaving 190 studies including ten multi-arm studies (Table 1). The pneumonia counts for control and intervention groups for each study are presented (Electronic supplementary material: ESM Tables S1 – S7).

**Table 1:** Characteristics of studies

Characteristics	Non-antimicrobial	Antimicrobial duplex	Antimicrobial
Review characteristics			
Number of reviews	9	2	2
Sources [References]	[1-9]	[10-13]	[10-13]
Listed studies^a^	236	16	68
Excluded studies			
• Duplicate	3	0	0
• NCC/ < 50% MV^b^	3	0	2
• No pneumonia data	121	0	0
Listing (ESM table)	Table s1 – s5	Table s7	Tables s6 & s8
Study characteristics			
Eligible^c^	109	16	66
MV for > 48 h for < 90%^d^	8	0	5
Majority quality score^e^	51	7	45
Study publication year (range)	1986–2016	1992–2019	1987–2019
Control group characteristics			
Number of patients	8206	1170	4829
Number of groups^f^	115	16	65
Length of stay, mean, days	11.8	12.9	13.6
95% CI	10.5–13.0	9.9–15.9	12.1–15.2
Group mean age, years 95% CI	52 43–58	48 38–59	53 43–58
Number of patients per group Med (IQR)	44 28 – 71	39 34 – 109	58 31—92
Contrast-based analysis as for RCCT			
VAP prevention effect^g^ (odds ratio; 95% CI; n)	0.82; 0.71—0.93 (122)	0.79; 0.54—1.14 (16)	0.39; ^h^ 0.31—0.48 (68)
Heterogeneity			
• Q	229	34.4	203.7
• df	121	15	67
• Tau^2^	.241	.254	.49
• I^2^	50.4	53.4	72.7
• H2	2.02	2.14	3.67
Arms-based analysis as for DTA			
DOR ^i^ (odds ratio; 95% CI; n)	0.82; 0.71—0.94 (122)	0.84; 0.54—1.3 (16)	0.37; ^j^ 0.3—0.47 (68)
SROC plot	Fig. 2	Fig. 3	Fig.4

^a^Number of studies listed in the original Cochrane review, including one study listed as both as antimicrobial and antimicrobial duplex

^b^NCC = non concurrent control; <50% MV = less than 50% of patients were receiving mechanical ventilation

^c^The number eligible are the numbers meeting inclusion criteria for this analysis

^d^Studies for which less than 90% of patients were reported to receive > 48 hours of MV

^e^Majority quality score derived as meeting the majority of quality criteria as scored in each of Cochrane review

^f^After exclusion of duplicate control groups, the number of groups is less than the number of studies

^g^Derived using random effect model

^h^Pneumonia prevention ES for Antimicrobial studies with control group pneumonia incidence > 40% is 0.26 (0.18 – 0.35; *n* = 35) and for studies with control group pneumonia incidence < 40% is 0.59 (0.48 – 0.73; *n* = 33)

^i^Derived using metandi

^j^Pneumonia prevention ES for Antimicrobial studies as DOR derived by metandi with control group pneumonia incidence > 40% is 0.25 (0.18 – 0.34; *n* = 35) and for studies with control group pneumonia incidence < 40% is 0.58 (0.47 – 0.71; *n* = 33). ESM Fig S9

Most studies were published between 1990 and 2010. There were 21 broad types of interventions among the non-antimicrobial RCCT’s and 28 different topical antiseptic, oral care or topical antibiotic intervention regimens among the antimicrobial RCCT’s. The group mean LOS, the group mean age and the publication year were similar across the sub-categories of studies. A majority quality score was awarded to 45 of 66 (68%) antimicrobial intervention RCCT’s but only 51 of 109 (47%) non-antimicrobial intervention RCCT’s (Table 1).

### Contrast-based analysis

The summary intervention ES’s for the three categories of study are listed in Table 1 together with ES heterogeneity estimates derived from a random effects meta-analysis. Of interest, the ES estimates derived from the arms-based (as ‘diagnostic’ odds ratios from the ‘metandi’ command) are each similar to those derived by the contrast-based analysis.

In the analysis of sub-categories, the summary intervention ES (as OR’s) for VAP prevention for each of the six broad sub-categories of intervention were in each case similar to those as listed (mostly as RR’s) in the original systematic reviews (ESM Table S8; ESM Fig S1 – S8). The ES heterogeneity estimates are highest for the two subcategories of antimicrobial based interventions.

### Arms-based analysis

The DTA methods estimate summary pneumonia incidence proportions for intervention and for control arms separately (Table 2).

**Table 2:** Pneumonia incidences and heterogeneity statistics^a^

	Summary proportion %	SE	95% CI	Q	df	tau^2^	I^2^%	H2	95% PI
All studies									
Control (*n* = 196)	27	3.2	24—30	1658	195	.84	90.2	10.2	5.7—69
Intervention (*n* = 206)	19	2.3	17—20	1137	205	.533	83.1	5.9	5.1—49
Non-antimicrobial									
Control (*n* = 115)	23	3.7	20—26	700	114	.671	86.5	7.4	5.5—60
Intervention (*n* = 122)	19	3.1	17—22	628	121	.564	82.9	5.8	5.1—52
Antimicrobial duplex									
Control (*n* = 16)	20	8.2	14—27	87	15	.491	83.5	6.0	4.9—54
Intervention (*n* = 16)	16	7.9	12—23	108	15	.543	83.3	6.0	3.7—51
Antimicrobial^b^									
Control (*n* = 65)	37	5.9	32—43	613	64	.87	92.1	12.6	8.2—79
Intervention (*n* = 68)	18	3.9	16—21	388	67	.499	83.3	6.0	5.1—48

^a^Summary proportions were derived by pooling the logit transformed study proportions using the Stata command “meta esize’ with the ‘logitprop’ option and then with back-transformation to percentages

^b^A sensitivity analysis for RCCT’s of Antimicrobial interventions stratified by whether the control group pneumonia incidence was > 40% versus <40% is presented in Table S10 and as Fig S9

For the intervention groups, paradoxically, the summary pneumonia incidences for the three categories were similar with each being in the range 16 to 19% and similar also with all 206 intervention groups combined as were the respective tau^2^, I^2^ and H2 heterogeneity metrics.

For the control groups of the antimicrobial RCCT’s, by contrast, these differed strikingly in three respects to all other categories of control and intervention group. The summary VAP incidences were higher by > 10 percentage points, the associated incidence heterogeneity metrics (tau^2^ = 0.87; I^2^ = 92.1% and H2 = 12.6) were highest, being even higher than for all 196 intervention groups combined, and the 95% prediction limits were widest (8.2 – 79%). These findings are broadly similar when the control groups of the sub-categories of the antimicrobial versus the non-antimicrobial RCCT’s are examined separately (ESM Table S10). Strikingly, the incidence heterogeneity metrics for control groups from the antimicrobial RCCT’s are higher than those from the intervention groups from the antimicrobial RCCT’s.

**Fig. 2:**
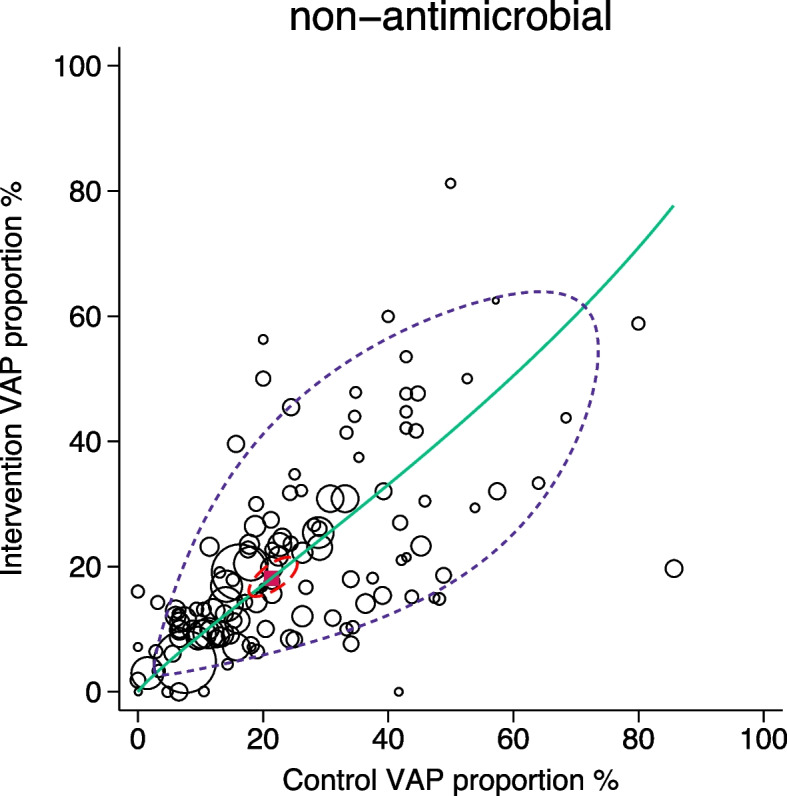
SROC with 95% confidence limits (dotted red inner ellipse) and 95% prediction limits (dotted purple outer ellipse) of pneumonia incidence among control and intervention groups (symbol size proportional to group size) of non-antimicrobial based pneumonia [VAP] prevention interventions drawn from nine Cochrane reviews. Also shown is the summary point (solid red square) and the hierarchical summary ROC curve (green). Note that in this adaptation of the SROC plot to visualize RCCT data in the DTA framework, the intervention group incidence equates to ‘sensitivity’ and the control group incidence equates to 1 minus ‘specificity’

**Fig. 3:**
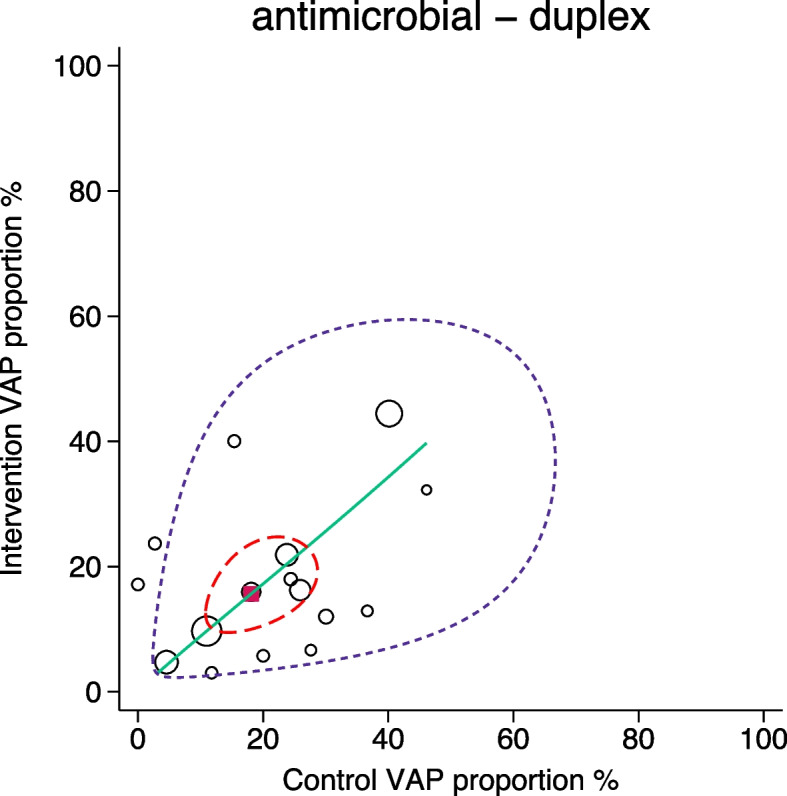
SROC with 95% confidence limits (dotted red inner ellipse) and 95% prediction limits (dotted purple outer ellipse) of pneumonia incidence among control and intervention groups (symbol size proportional to group size) of duplex antimicrobial based pneumonia [VAP] prevention interventions drawn from four Cochrane reviews. Also shown is the summary point (solid red square) and the hierarchical summary ROC curve (green). Note that in this adaptation of the SROC plot to visualize RCCT data in the DTA framework, the intervention group incidence equates to ‘sensitivity’ and the control group incidence equates to 1 minus ‘specificity’

**Fig. 4:**
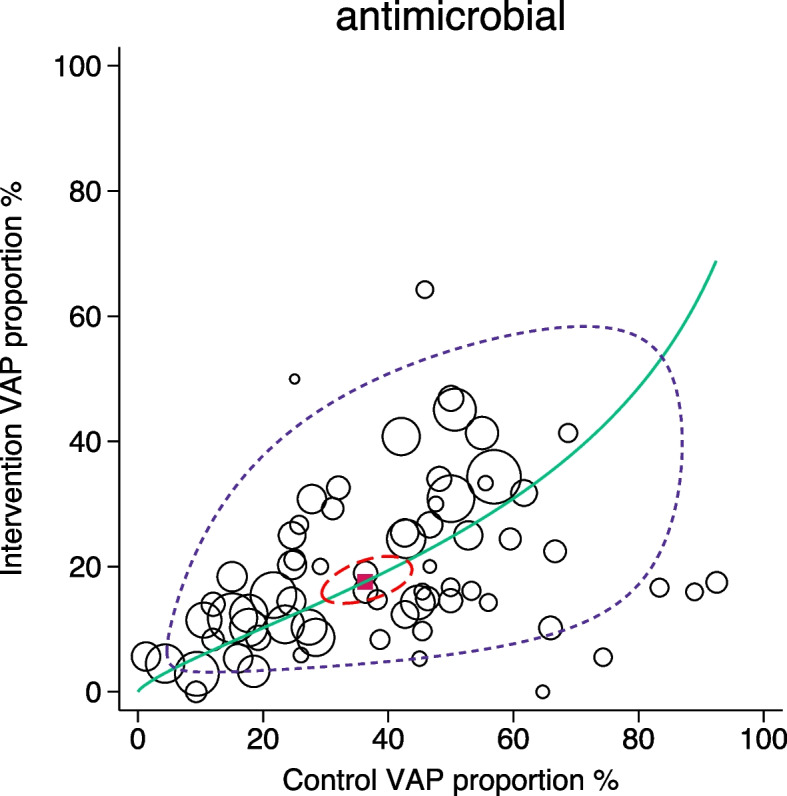
SROC with 95% confidence limits (dotted red inner ellipse) and 95% prediction limits (dotted purple outer ellipse) of pneumonia incidence among control and intervention groups (symbol size proportional to group size) of antimicrobial based pneumonia [VAP] prevention interventions drawn from four Cochrane reviews. Also shown is the summary point (solid red square) and the hierarchical summary ROC curve (green). Note that in this adaptation of the SROC plot to visualize RCCT data in the DTA framework, the intervention group incidence equates to ‘sensitivity’ and the control group incidence equates to 1 minus ‘specificity’

The SROC plots are displayed in Figs. 2, 3 and 4. Because the interventions prevent pneumonia, most results appear below the y = x line. For the non-antimicrobial interventions, which are relatively ineffective, the study results are close to the y = x line and the 95% prediction ellipse is slim. For the antimicrobial intervention RCCT’s, the results are dispersed away from the y = x line and the prediction ellipse is wide along the x-axis. For the duplex antimicrobial intervention RCCT’s, the results straddle the y = x line and the prediction ellipse is squat.

The SROC plots generated with ‘metandiplot’ [13] and ‘metadta’ [14] were in most cases similar (ESM Fig S1 – S8) to each other within each sub-categories.

The SROC plot was repeated for the antimicrobial intervention RCCT’s limited to those with control groups for which the pneumonia incidence is < 40% (Fig s9). The 95% prediction ellipse is narrower, and both the incidence heterogeneity (Table 2) and the summary ES (as a contrast-based OR, Table 1) is attenuated in comparison to that with all antimicrobial RCCT’s combined.

#### Simulation studies

The results of simulations of positive and negative spillover applied to the studies of non-antimicrobial interventions are presented in Table 3 and in Figures s10-s12. The magnitude of the control group heterogeneity metrics under simulations of negative spillover approached those derived for the duplex study control groups. The associated odds ratio under simulations of negative spillover became non-significantly different from unity.

**Table 3:** Non-antimicrobial control group pneumonia incidences and heterogeneity statistics in spillover simulation studies^a^

Spillover	SROC metrics^b^			Control group metrics^c^									
	Odds ratio	95% CI	SROC Fig	Summary proportion %	SE	95% CI	Q	df	tau^2^	I^2^ %	H2	95% PI	
No spillover^d^	0.82	0.71-0.94	Fig 2	23	3.7	20 - 26	731	114	.671	86.5	7.4	5.5 - 60	
Positive spillover (uniform)^e^													
+0.025 spillover	0.68	0.6-0.78	Fig S10a	25.3	3.4	22.5 – 28.3	693	114	.57	85.5	6.9	7 – 60.4	
+0.05 spillover	0.58	0.51-0.66	Fig S10b	28.2	3.3	25.3–31.2	661	114	.51	84.9	6.6	8.7 – 61.7	
+0.1 spillover	0.45	0.39-0.51	Fig S10c	33.1	3.1	30.3–36	586	114	.38	82.9	5.8	12.6 – 62.9	
Positive spillover (partial)^f^													
+0.1 spillover (half)	0.59	0.51-0.68	Fig S11a	27.9	3.5	24.9 – 31	720	114	.57	86.3	7.3	7.9 – 63.6	
+0.1 spillover (quarter)	0.7	0.6-0.81	Fig S11b	25.1	3.6	22.2 – 28.2	769	114	.645	86.4	7.4	6.3 – 62.4	
+0.2 spillover (quarter)	0.62	0.52-0.73	Fig S11c	27.2	3.8	24 - 30.6	889	114	.699	87.7	8.1	6.6-66.4	
Negative spillover (uniform)^g^													
-0.025 spillover	0.94	0.81-1.1	Fig S12a	20.0	3.7	17.3 – 23.1	761	114	.795	86.8	7.6	4.1 – 60	
-0.05 spillover	1.13	0.94-1.35	Fig S12b	17.2	3.8	14.6 – 20.2	769	114	.93	87.1	7.7	3 – 58.7	

^a^*Abbreviations*; *95% CI* 95% confidence interval, *SE* Standard error, *95% PI* 95% Prediction interval, *df* Degrees of freedom

^b^Odds ratio derived as the diagnostic odds ratio as shown in the SROC plots

^c^Control group statistics were derived by pooling the logit transformed study proportions using the Stata command “meta esize’ with the ‘logitprop’ option and then with back-transformation to percentages

^d^No spillover equates to the conditions as in Fig 1c

^e^Positive and uniform spillover simulated as an pneumonia count increase of 2.5, 5 or 10 (numerator) per 100 control group patients (denominator) respectively. This equates to the conditions as in Fig 1e

^f^Positive and partial spillover simulated as an increase pneumonia count of 10 or 20 (numerator) per 100 control group patients (denominator) in half or a quarter of control groups respectively. This equates to the conditions as in Fig 1f

^g^Negative and uniform simulated as a decrease pneumonia count of 2.5 or 5 (numerator) per 100 control group patients (denominator) respectively. This equates to the conditions as in Fig 1d

The magnitude of the control group heterogeneity metrics under simulations of positive spillover approached those derived for the antimicrobial control groups. The associated odds ratios shifted further away from unity under simulations of positive spillover. These changes in control group heterogeneity metrics were most evident under simulations of partial positive spillover.

## Discussion

Where an infection prevention intervention appears effective within an RCCT, three questions follow which will be not be answerable from a contrast-based analysis. How much of the apparent ES is attributable to a direct effect of the intervention on the intervention group patients versus how much results from an indirect effect arising as an altered infection risk in the concurrent control group patients? Secondly, can the findings be projected towards population targets such as ‘pneumonia zero’? Thirdly, does the intervention ES vary with underlying (i.e. control group) risk and what is the nature of any covariation?

Designing a study to answer these three questions and establish the population safety of antimicrobials used as an intervention to prevent infections within ICU populations would be challenging both logistically and ethically [50].

Methods applicable to DTA analysis enable these questions to be addressed within an arms-based framework from three approaches. In addressing question one, all three approaches indicate greater dispersion among the control groups (Table 2, Figs. 2, 3 and 4) of the antimicrobial category of RCCT’s and also when the RCCT’s are analysed by sub-category (Table s10). The dispersion resembles that for Fig. 1f. Paradoxically, the dispersion among the intervention groups of the antimicrobial category of RCCT’s are similar to both that among the control groups and also to that among the intervention groups of the non-antimicrobial category of RCCT’s.

For question two, relating to projections to population targets and whether zero might be achievable [51], it can readily be appreciated from Figs. 2, 3 and 4 for all intervention categories that few intervention groups achieved an intervention group pneumonia incidence below the clinically relevant range, whether 5% [28] or 8% [29].

In relation to question three, the variation in ES with underlying risk, the intervention ES derived from an analysis restricted to those antimicrobial RCCT’s with control group pneumonia incidence > 40% is enhanced compared to an ES derived including all antimicrobial RCCT’s (Table s10). The traditional contrast-based analysis in conjunction with a presumption that SUTVA is valid would infer that this indicates increased antimicrobial ES in association with increased underlying control group risk [52]. However, from an arms-based analysis and in the simulations as undertaken here, which does not presume SUTVA, the inference is ‘flipped’ given that the summary incidence of the antimicrobial intervention groups of the high pneumonia group is 25%, being at the upper end of the clinically relevant range [28, 29] and the corresponding control group incidences are unusually disperse. Moreover, pneumonia incidences below the lower limit of the clinically relevant range, whether conservative (8%, [29]) or liberal (5% [28]) are paradoxically rare regardless of the underlying risk (Table s10, Fig s9).

Three other observations, reported elsewhere, support the inference of positive spillover from the intervention groups among the antimicrobial RCCT’s. Firstly, studies of antimicrobial interventions appear ineffective within studies with a CRT versus RCCT study design (CRT design studies are generally excluded from Cochrane reviews). Moreover, the CRT control and intervention groups have event rates within the clinically relevant benchmark range [53, 54]. Of note, there is no opportunity for spillover in a CRT.

For example, the prevention of mortality has recently been compared in a large systematic review of antimicrobial based prevention within ICU patients using TAP. Among 27 RCCT’s (5699 patients) of antimicrobial-based interventions, there is a summary 15-percentage point mortality difference between control versus intervention groups whereas by contrast for three large CRT’s (18,335 patients) there is a summary zero-percentage point difference [55].

Second, there are discrepancies in several microbiologically documented pneumonia and bacteraemia end points among the RCCT studies such that the control groups have patterns of isolates resembling those in the antimicrobial intervention groups [56–58].

Third, even in analyses adjusting for group level measures of underlying risk, such as group mean length of ICU stay, year of study publication and group mean age, the pneumonia incidences remain unaccountably higher among concurrent control groups of RCCT’s [48].

Of note, cross infection from both ICU staff and the ICU environment is widespread but usually inapparent [59–61]. Rebound patient and ICU colonization from the cessation of antimicrobial interventions is a difficult to quantify driver [47, 63–65]. The group level incidence of VAP associated with *Staphylococcus aureus* shows evidence of rebound in association with prolonged length of ICU stay [58].

### Limitations

Among RCCT’s included within the 13 Cochrane reviews, there is substantial clinical heterogeneity in the populations, modes of pneumonia diagnosis, length of ICU stay, group size, study quality, and study designs among studies published over several decades included in the analysis here. Hence the amount of any spillover effect will be context specific.

There is also considerable heterogeneity in the antimicrobial and also non-antimicrobial interventions as well as use of alternate interventions as part of ‘standard care’ among the control groups of non-antimicrobial RCCT’s which might be expected to add to the overall heterogeneity. In the case of the antimicrobial RCCT’s, this was able to be addressed in that the RCCT’s receiving protocolized antimicrobial prophylaxis (duplex antimicrobial RCCT’s) were removed into a separate third category.

Another limitation is that the influence of publication bias and the possibility of missing studies has not been factored here.

Despite these limitations, it is striking that in all comparisons, the dispersion in pneumonia incidence, either as heterogeneity metrics (Table 2) or more strikingly, visually (Figs. 2, 3 and 4), is most apparent among the control groups of antimicrobial RCCT’s.

Spillover with vaccination interventions used to prevent infection usually causes a beneficial effect [6–8]. How a beneficial spillover effect might be diagnosed has not been explored here in detail. However, the duplex RCCT’s, wherein the control groups were protocolized to receive partial antimicrobial intervention, wherein a negative spillover could be construed as constituted within the study design, gives an impression and provides an aditional perspective. For the category of duplex RCCT’s, the prediction ellipse is widened in both directions. However, with only 16 RCCT’s, there are too few groups to judge its shape or the heterogeneity metrics.

This tutorial here explores spillover among RCCT’s of antimicrobial based interventions to prevent VAP in the ICU context as postulated in the first such study [44]. In addition to the simulation studies undertaken here, the data from these RCCT’s as used here can be used to simulate a CRT [66] as evidence of a mortality spillover which is associated with unusually high mortality in these RCCT’s [66].

## Conclusion

Spillover, whether beneficial or harmful, cannot be diagnosed within a contrast-based framework and an arms-based framework as used for DTA is required. Spillover among RCCT’s of antimicrobial interventions used to prevent pneumonia among ICU patients is diagnosed from a higher and more dispersed pneumonia incidence among the control groups of antimicrobial RCCT’s. The arms-based framework enables three approaches to assessing this increased incidence dispersion; by comparison to intervention groups of antimicrobial RCCT’s, by comparison to control groups of non-antimicrobial RCCT’s and, by comparison to the clinically relevant incidence range. The spillover would not be apparent within either individual RCCT’s nor within systematic reviews examined using a contrast-based framework which assume, without evidence, that SUTVA is valid. Moreover, this spillover would perversely conflate the appearance of benefit of antimicrobial based interventions used to prevent pneumonia in ICU patients. Also, any inferred association between apparent increasing ES with increasing underlying risk among an aggregate of studies would be ‘flipped’.

### Supplementary Information

Supplementary Material 1.

## Data Availability

No datasets were generated or analysed during the current study.

## Abbreviations

SUTVA: Stable Unit Treatment Variable Assumption
SROC: Summary receiver operator characteristic
RCCT: Randomized concurrent controlled trials
ES: Effect size
DTA: Diagnostic test assessment
OR: Odds ratio
RR: Risk ratio
RD: Risk difference
VAP: Ventilator associated pneumonia
ICU: Intensive care unit
UGIT: Upper gastro-intestinal tract
ESM: Electronic supplementary material
CI: Confidence interval
PI: Prediction interval

## Glossary

Spillover effect: An indirect effect mediated by contagion occurring within populations originating from those who receive an intervention of interest to impact those individuals that do not.
Negative spillover: An infection prevention effect applied to an intervention group which causes a decrease in infection incidence among concurrent and collocated control group patients.
Positive spillover: An infection prevention effect applied to an intervention group which causes (paradoxically) an increase in infection incidence among concurrent and collocated control group patients. This could result from the impact of the intervention on the microbiome of the entire population and an increase in contagion.
SUTVA (Stable Unit Treatment Variable Assumption): (SUTVA Part 1) The response of a particular unit depends only on the treatment to which that unit was assigned, not the treatments of others around them and (SUTVA Part 2) The potential outcomes for any unit do not vary with the treatments assigned to other units.
Dispersion: The amount by which a set of observations deviate from their mean or central value.
Heterogeneity: The extent to which a group of observations deviate from each other.
