# Supplementary material for: Visualizing and diagnosing spillover within randomized concurrent controlled trials through the application of diagnostic test assessment methods
Source: BMC Med Res Methodol. 2024 Aug 16;24:182. doi: 10.1186/s12874-024-02296-1 (PMC11328391; doi:10.1186/s12874-024-02296-1)

JC Hurley

Contents	Data	Contrast based (Forrest plots)	Arms based (SROC)
**Non-antimicrobial interventions**			
Upper GI tract (UGIT) interventions			
• Interventions versus placebo	Table s1	Fig s1a	Fig s1 b&c
• Interventions versus other	Table s2	Fig s2a	Fig s2 b&c
Feeding interventions			
• EN versus PN<br>• EN versus EN + PN<br>• EN versus late EN<br>• PP versus gastric	Table s3	Fig s3a	Fig s3 b&c
Airway interventions			
• Closed versus open tracheal suction (CTSS)<br>• Heat and moisture exchange (HME)<br>• Silver endotracheal tube (ETT)<br>• Semi-recumbent	Table s4	Fig s4a	Fig s4 b&c
Probiotic interventions	Table s5	Fig s5a	Fig s5 b&c
**Antimicrobial interventions**			
Antiseptic and oral care interventions	Table s6	Fig s6a	Fig s6 b&c
Antimicrobial duplex interventions	Table s7	Fig s7a	Fig s7 b&c
Antibiotic interventions	Table s8	Fig s8a	Fig s8 b&c
SROC plot: Antimicrobial interventions (Control pneumonia < 40% vs >40%)			Fig s9 a&b
**Simulation studies**			
Non-antimicrobial RCCT's with simulated positive uniform spillover			Fig s10a, b & c
Non-antimicrobial RCCT's with simulated positive partial spillover			Fig s11a, b & c
Non-antimicrobial RCCT's with simulated negative uniform spillover			Fig s12a, & b
Summary of contrast-based analysis by sub-category versus Cochrane review findings	Table s9		p 11
Summary of arms-based analysis by sub-category	Table s10		p 12 - 13
References			p 14 – 22
**Stata script [Stata version 18];**			p 23
Caterpillar & SROC plots (Fig s1 – s12)			p 24- 44

Abbreviations

UC = usual care; H2RA = histamine receptor antagonists; EN = enteral nutrition; PN = parenteral nutrition; TAP = topical antibiotic prophylaxis; PPAP = protocolized parenteral antibiotic prophylaxis

ivapn = intervention group patients with VAP
ivapm= intervention group patients without VAP
cvapn = control group patients with VAP
cvapm= control group patients without VAP

Table s1: Upper GI tract (UGIT) Interventions versus placebo

Author & notes	year	ref	intervention	Patients			
				ivapn	ivapm	cvapn	cvapm
**Apte**	1992	1	H2RA v pl	13	3	9	9
**Ben-Menachem**	1994	2	H2RA v pl	13	87	3	47
**Ben-Menachem**	1994	2	Suc v pl	12	88	3	47
**Eddleston '94**	1994	3	Suc v pl	1	13	0	12
**Hanisch**	1998	4	H2RA v pl	10	47	6	22
**Hanisch**	1998	4	Ach v pl	10	34	6	22
**Kantorova**	2004	5	H2RA v pl	7	64	5	70
**Kantorova**	2004	5	PPI v pl	8	64	5	70
**Kantorova**	2004	5	Suc v pl	6	63	5	70
**Karlstadt**	1990	6	H2RA v pl	1	53	0	33
**Martin**	1993	7	H2RA v pl	0	56	4	57
**Metz**	1993	8	H2RA v pl	12	72	15	64
**Yildizdas**	2002	9	H2RA v pl	20	22	6	8
**Yildizdas**	2002	9	PPI v pl	17	21	6	8
**Yildizdas**	2002	9	Suc v pl	16	22	6	8

Table s2: Upper GI tract (UGIT) Interventions versus other

Author & notes	year	ref	intervention	Patients			
				ivapn	ivapm	cvapn	cvapm
**Behrens**	1994	10	Ach v H2RA	0	22	1	20
**Ben-Menachem**	1994	2	H2RA v Suc	13	87	12	88
**Bonten '95**	1995	11	Atc v Suc	16	58	15	52
**Cioffi**	1994	12	Suc v Ac	9	41	17	33
**Conrad**	2005	13	PPI v H2RA	17	164	20	158
**Cook**	1998	14	Suc v H2RA	114	482	98	506
**de Azevedo**	2000	15	H2RA v PPI	5	33	4	34
**de Azevedo**	2000	15	PPI v Suc	5	33	3	30
**Driks**	1987	16	Suc v Ac	16	53	7	54
**Eddleston '91**	1991	17	Suc v H2RA	3	27	10	20
**Ephgrave**	1998	18	Atc v Suc	11	59	15	55
**Fabian**	1993	19	Suc v H2RA	15	50	52	127
**Fogas**	2013	20	PPI v H2RA	9	29	10	31
**Hanisch**	1998	4	Ac v H2RA	10	34	10	47
**Kantorova**	2004	5	H2RA v Suc	7	64	6	63
**Kappstein**	1991	21	Suc v H2RA	25	30	12	37
**Khorvash**	2014	22	Suc v PPI	10	61	24	42
**Laggner**	1989	23	Suc v H2RA	2	14	1	15
**Lee**	2014	24	H2RA v PPI	1	29	1	29
**Levy**	1997	25	PPI v H2RA	5	30	1	31
**Lin**	2016	26	Other v PPI	6	54	4	56
**Mahul**	1992	27	Suc v Ac	17	55	13	60
**Maier**	1994	28	Suc v H2RA	14	37	10	37
**Martin**	1980	29	H2RA v Ac	12	28	7	30
**Mustafa**	1994	30	Suc v H2RA	9	7	3	12

Table s2: UGIT Interventions versus other - continued

Author & notes	year	ref	intervention	Patients			
				ivapn	ivapm	cvapn	cvapm
**Phillips**	1998	31	PPI v H2RA	4	21	0	33
**Pickworth**	1993	32	Suc v H2RA	5	39	6	33
**Prakash**	2008	33	Suc v H2RA	15	10	10	15
**Prod'hom**	1994	34	Suc v H2RA	10	73	21	59
**Prod'hom**	1994	34	Suc v Ac	18	63	21	59
**Ryan_S**	1993	35	Suc v H2RA	7	49	8	50
**Simms_S**	1991	36	Suc v H2RA	8	22	9	23
**Sirvent**	1994	37	Suc v H2RA	11	14	9	17
**Solouki**	2009	38	PPI v H2RA	6	62	8	53
**Somberg**	2008	39	H2RA v PPI	3	32	16	151
**Thomason**	1996	40	H2RA v Suc	42	94	43	97
**Thomason**	1996	40	H2RA v Ac	42	94	49	99
**Tryba**	1987	41	Ac v Suc	3	26	11	21
**Tryba**	1988	42	Ac v H2RA	6	194	3	197
**Yildizdas**	2002	9	H2RA v PPI	20	22	17	21

Table s1 & s2 footnotes

ivapn = intervention group patients with VAP

ivapm= intervention group patients without VAP

cvapn = control group patients with VAP

cvapm= control group patients without VAP

Interventions; UC = Usual care; H2RA = Histamine 2 receptor antagonists; PPI = Proton pump inhibitors; Suc = sucralfate; Atc = antacids; Ach = Anticholinergics; Pg = Prostaglandin inhibitors.

Notes;

1. Davies 2012 analysed as an enteral nutrition study (Table s3).
2. Fan 2016 analysed as an enteral nutrition study (Table s3)
3. Behrens 1994 & Yildizdas 2002 are pediatric studies
4. Martin 1993 & Tryba 1987; Patients with pneumonia on study entry were not counted in the pneumonia denominator.

Data provenance

Ref 1 – 9 from Analysis 1.2 of Ref 191

Ref 10-42 from Analysis 9.2, 10.2. 11.2, 12.2, 15.2, 18.2, 22.2 of Ref 191

191 Toews_I, George_AT, Peter_JV, Kirubakaran_R, Fontes_LES, Ezekiel_JPB, Meerpohl_JJ. Interventions for preventing upper gastrointestinal bleeding in people admitted to intensive care units. *Cochrane Database of Systematic Reviews* 2018, Issue 6. Art. No.: CD008687.

Table s3: Feeding interventions

Author & notes	year	ref	intervention	Patients			
				ivapn	ivapm	cvapn	cvapm
**Feeding interventions**							
**Adams**	1986	43	EN	11	12	8	15
**Altintas**	2011	44	EN	5	25	11	30
**Borzotta**	1994	45	EN	15	13	9	12
**Fan**	2016	46	EN	20	20	8	32
**JustoMeirelles**	2011	47	EN	2	10	2	8
**Kudsk**	1992	48	EN	6	45	14	31
**Young**	1987	49	EN	9	19	6	17
**Wischmeyer**	2017	50	EN	18	55	12	40
**Chourdakis**	2012	51	late EN	20	14	20	5
**Eyer**	1993	52	late EN	4	15	8	11
**Hill**	2002	53	late EN	4	18	9	15
**Moses**	2009	54	late EN	12	17	10	20
**Nguyen**	2008	55	late EN	6	8	3	11
**Acosta-escribano**	2010	56	SB feeding	16	34	31	23
**Day**	2001	57	PP_EN	1	13	2	9
**Davies**	2002	58	SB feeding	2	29	1	34
**Davies**	2012	59	SB feeding	18	73	19	70
**Hsu**	2009	60	SB feeding	5	54	15	47
**Kearns**	2000	61	SB feeding	4	17	3	20
**Kortbeek**	1999	62	SB feeding	10	27	18	25
**Montecalvo**	1992	63	SB feeding	0	19	2	17
**Montejo**	2002	64	SB feeding	16	34	20	31
**White**	2009	65	SB feeding	5	45	11	43

Table s3 footnotes

Interventions; PN = Parenteral nutrition; EN = Enteral nutrition; TPN = Total parenteral nutrition; G_EN is gastric EN; PP_EN is post pyloric EN

Notes;

1. Borzotta 1994 & Kudsk 1992; Patients with early mortality were not counted in the pneumonia denominator.
2. Fan & Davies are duplicate studies also abstracted in Toews [191]

Data provenance

192 Lewis SR, Schofield-Robinson OJ, Alderson P, Smith AF. Enteral versus parenteral nutrition and enteral versus a combination of enteral and parenteral nutrition for adults in the intensive care unit. Cochrane Database of Systematic Reviews 2018, Issue 6. Art. No.: CD012276.

193 Padilla PF, Martínez G, Vernooij RW, Urrútia G, i Figuls MR, Cosp XB. Early enteral nutrition (within 48 hours) versus delayed enteral nutrition (after 48 hours) with or without supplemental parenteral nutrition in critically ill adults. Cochrane Database of Systematic Reviews. 2019(10).

194 Alkhawaja S, Martin C, Butler RJ, Gwadry-Sridhar F. Post-pyloric versus gastric tube feeding for preventing pneumonia and improving nutritional outcomes in critically ill adults. Cochrane Database of Systematic Reviews. 2015(8).

Table s4: Airway interventions.

Author & notes	year	ref	intervention	Patients			
				ivapn	ivapm	cvapn	cvapm
**OTSS vs CTSS**							
**Adams**	1997	66	CTSS	0	10	0	10
**Combes**	2000	67	CTSS	4	50	9	41
**Conrad**	1989	68	CTSS	6	10	6	11
**Deppe**	1990	69	CTSS	12	34	11	27
**Johnson**	1994	70	CTSS	8	8	10	9
**Lorente'05**	2006	71	CTSS	43	167	42	191
**Lorente'06a**	2005	72	CTSS	33	203	31	190
**Rabitsch**	2004	73	CTSS	0	12	5	7
**Topeli**	2004	74	CTSS	13	28	9	28
**Welte**	1997	75	CTSS	9	18	16	9
**Zeitoun**	2003	76	CTSS	7	16	11	13
**HH vs HME**							
**Alcoforado**	2012	77	HME	5	3	4	3
**Boots**	1997	78	HME	6	36	7	34
**Boots**	2006	79	HME	32	158	27	164
**Branson**	1996	80	HME	3	46	3	51
**Diaz**	2002	81	HME	8	15	5	15
**Dreyfuss**	1991	82	HME	6	55	8	62
**Hurni**	1997	83	HME	5	54	7	49
**Kirton**	1997	84	HME	10	130	22	118
**Kollef '98**	1998	85	HME	15	148	15	132
**Lacherade '05**	2005	86	HME	47	138	53	131
**Lorente'06b**	2006	87	HME	21	32	8	43
**Martin**	1990	88	HME	2	29	8	34
**Memish**	2001	89	HME	14	109	19	101
**Roustan**	1992	90	HME	5	50	9	52
**Silver ETT**							
**Kollef'08**	2008	91	silver	37	729	56	687
**Supine vs SR**							
**Cai**	2006	92	semir	4	23	13	14
**Drakulovic**	1999	93	semir	3	36	16	31
**Hang**	2012	94	semir	3	17	9	10
**Hu**	2012	95	semir	8	35	21	22
**van Nieuwenhoven**	2006	96	semir	13	99	8	101
**Keeley**	2007	97	semir	5	12	7	6
**Leng**	2012	98	semir	5	23	5	28
**Wu**	2009	99	semir	11	45	48	8
**Xue**	2012	100	semir	4	44	12	36
**Yu**	2012	101	semir	5	28	14	18

Table s4: Airway interventions
Interventions; OTSS = Open tracheal suction systems; CTSS = Closed tracheal suction systems; HH = Heated humidifier; HME heat and moisture exchangers; Sup = supine; semir – semi-recumbent; semir30 – semi-recumbent at 30 ; semir45 – semi-recumbent at 45; Silver = Silver coated Endotracheal tube; UC – usual care.

Data provenance
195 Solà I, Benito S. Closed tracheal suction systems versus open tracheal suction systems for mechanically ventilated adult patients. Cochrane Database of Systematic Reviews 2007, Issue 4. Art. No.: CD004581.
196 Gillies D, Todd DA, Foster JP, Batuwitage BT. Heat and moisture exchangers versus heated humidifiers for mechanically ventilated adults and children. Cochrane Database of Systematic Reviews 2017, Issue 9. Art. No.: CD004711.
197 Wang L, Li X, Yang Z, Tang X, Yuan Q, Deng L, Sun X. Semi-recumbent position versus supine position for the prevention of ventilator-associated pneumonia in adults requiring mechanical ventilation. Cochrane Database of Systematic Reviews 2016, Issue 1. Art. No.: CD009946.
198 Tokmaji G, Vermeulen H, Müller MCA, Kwakman PHS, Schultz MJ, Zaat SAJ. Silver-coated endotracheal tubes for prevention of ventilator-associated pneumonia in critically ill patients. Cochrane Database of Systematic Reviews 2015, Issue 8. Art. No.: CD009201

Notes;
1. Kollef 2008 is the only eligible study from the Cochrane review of Silver coated Endotracheal tube [S198].

Table s5: Probiotic interventions

Author & notes	year	ref	intervention	Patients			
				ivapn	ivapm	cvapn	cvapm
**Barraud**	2010	102	probiotic	23	64	15	65
**Forestier**	2008	103	probiotic	24	78	24	82
**Klarin**	2008	104	probiotic	1	22	3	18
**Knight**	2009	105	probiotic	12	118	17	112
**Kotzampassi**	2006	106	probiotic	15	21	16	20
**Morrow**	2010	107	probiotic	17	56	33	40
**Spindler-Vesel**	2007	108	probiotic	4	22	34	53
**Tan**	2011	109	probiotic	7	9	13	6

Interventions; UC – usual care; pbtic = probiotic

Data provenance
199 Bo L, Li J, Tao T, Bai Y, Ye X, Hotchkiss RS, Kollef MH, Crooks NH, Deng X. Probiotics for preventing ventilator-associated pneumonia. Cochrane Database of Systematic Reviews 2014, Issue 10. Art. No.: CD009066.

Table s6: Antiseptic and oral care interventions

Author & notes	year	ref	intervention	Patients			
				ivapn	ivapm	cvapn	cvapm
**Bellissimo-Rodrigues**	2009	110	Chlx	16	48	17	52
**Berry**	2011	111	Chlx	4	29	1	42
**Cabov**	2010	112	Chlx	1	16	6	17
**Chen**	2008	113	Chlx	16	44	28	32
**De Riso**	1996	114	Chlx	5	168	17	163
**Fourrier'00**	2000	115	Chlx	5	25	14	14
**Fourrier'05**	2005	116	Chlx	13	101	12	102
**Fu**	2019	117	Chlx	7	33	37	3
**Grap**	2011	118	Chlx	7	14	10	8
**Jacomo**	2011	119	Chlx	16	71	11	62
**Koeman-Ch**	2006	120	Chlx	13	114	23	107
**Kusahara**	2012	121	Chlx	15	31	16	34
**Meidani**	2018	122	Chlx	6	44	15	35
**Meinberg**	2012	123	Chlx	18	10	11	13
**Munro**	2009	124	Chlx	38	54	55	45
**Özçaka**	2012	125	Chlx	12	17	22	10
**Scannapieco**	2009	126	Chlx	14	83	12	37
**Sebastian**	2012	127	Chlx	12	29	14	31
**Tantipong**	2008	128	Chlx	5	53	10	42
**Tuon**	2017	129	Chlx	4	4	2	6
**Berry**	2011	111	Bicarb	4	29	1	42
**Berry**	2013	130	Bicarb	6	127	6	132
**Caruso**	2009	131	Saline	14	116	31	101
**Feng**	2012	132	PVI	18	53	29	39
**Feng**	2012	132	furacilin	8	57	29	39
**Hu**	2009	133	Saline	4	21	10	12
**Mo**	2016	134	saline	15	90	47	58
**Nobahar**	2019	135	Hperoxide	5	29	13	21
**Pobo**	2009	136	TB ±Chlx	15	59	18	55
**Prendergast**	2012	137	toothbrush	8	30	10	30
**Seguin**	2006	138	PVI	3	33	12	19
**Seguin**	2014	139	PVI	24	54	20	52
**Stefanescu**	2013	140	biotene	6	14	10	11
**Tang**	2013	141	saline	5	25	25	5
**Xu**	2007	142	saline rinse + swab	11	47	16	28
**Xu**	2007	142	saline rinse	10	52	16	28
**Xu**	2008	143	saline rinse	30	34	26	26
**Yao**	2011	144	TB±Chlx	4	24	14	11
**Zhao**	2012	145	triclosan	73	89	82	80

Table s6 & s7 footnotes; Interventions; UC = usual care; Chlx = Chlorhexidine; HPO = hydrogen peroxide; PVI = Povidone iodine; Hperoxide = hydrogen peroxide; TB = Toothbrushing.

Data provenance
Ref 110 – 129 from Analysis 1.1 of Ref 200 & Analysis 1.1 of Ref 201
Ref 111, 130-145 from Analysis 2.1, 3.1, 4.1 of Ref 200 & Analysis 3.1, 4.1, 5.1 of Ref 201

Table s7: Antimicrobial duplex interventions

Author & notes	year	ref	intervention	Patients			
				ivapn	ivapm	cvapn	cvapm
**Antiseptic**							
**Berry**	2013	130	Listerine v bicarb	6	121	6	127
**de Lacerda**	2017	146	Chlx v other	17	88	28	80
**Dahiya**	2012	147	Chlx v Hperoxide	2	33	7	28
**Hanifi**	2017	148	Chlx v other	14	21	6	33
**Irani**	2019	149	Chlx v other	6	29	0	35
**Khaky**	2018	150	Chlx v other	9	29	1	36
**Long**	2012	151	PVI v other	4	27	11	19
**Lorente'12**	2012	152	Chlx +TB	21	196	24	195
**Meidani**	2018	153	Chlx v PotP	6	44	7	43
**Panchabhai**	2009	154	Chlx v PotP	14	74	15	68
**Antibiotic**							
**Chaari**	2014	155	TAP_PPAP v PPAP	10	21	6	7
**Ferrer**	1994	156	PTA-Ctx v PPAP	7	32	10	31
**Hammond**	1992	157	TAP_PPAP v PPAP	25	89	30	96
**Laggner**	1994	158	GA v PPAP	1	32	4	30
**Lingnau**	1997	159	TAP_PPAP v PPAP	72	90	71	106
**Stoutenbeek**	1996	160	TAP_PPAP v PPAP	2	28	8	21

Table s7 footnotes

Data provenance
Ref 130, 146-154 from Analysis 2.1, 3.1, of Ref 201
Ref 155-160 from Table 2.4 of Ref 202 & Table 2.2.1 of Ref 203
200 Hua F, Xie H, Worthington HV, Furness S, Zhang Q, Li C. Oral hygiene care for critically ill patients to prevent ventilator-associated pneumonia. Cochrane Database of Systematic Reviews 2016, Issue 10. Art. No.: CD008367.
201 Zhao T, Wu X, Zhang Q, Li C, Worthington HV, Hua F. Oral hygiene care for critically ill patients to prevent ventilator-associated pneumonia. Cochrane Database of Systematic Reviews 2020, Issue 12. Art. No.: CD008367.

Interventions; UC = usual care; Chlx = Chlorhexidine; HPO = hydrogen peroxide; PotP = potassium permanganate; triclsn = triclosan; TB = Toothbrushing; TAP = topical antibiotic prophylaxis; PPAP = protocolized parenteral antibiotic prophylaxis;

Table s8: Antibiotic interventions

Author & notes	year	ref	intervention	Patients			
				ivapn	ivapm	cvapn	cvapm
**Antibiotic interventions**							
**Abele-Horn**	1997	161	PTA-Ctx	13	45	20	10
**Aerdts**	1991	162	TAP_PPAP	1	17	29	10
**Blair**	1991	163	PTA-Ctx	11	115	37	93
**Boland**	1991	164	TAP_PPAP	3	12	7	8
**Cockerill**	1992	165	TAP_PPAP	4	71	12	63
**de la Cal**	2005	166	PTA-Ctx	18	35	26	28
**Finch**	1991	167	PGA-Ctx	4	16	7	17
**Jacobs**	1992	168	PTA-Ctx	0	36	4	39
**Kerver**	1988	169	PTA-Ctx	5	44	31	16
**Krueger**	2002	170	TAP_PPAP	91	174	149	113
**Palomar**	1997	171	TAP_PPAP	10	31	25	17
**Rocha**	1992	172	PTA-Ctx	7	40	25	29
**Sanchez-Garcia**	1992	173	TAP_PPAP	32	99	60	80
**Stoutenbeek**	2007	174	TAP_PPAP	62	139	100	100
**Ulrich**	1989	175	PNoA_Tr	7	41	26	26
**Verwaest PTA**	1997	176	PTA-Ctx	31	169	40	145
**Winter**	1992	177	PTA-Cz	3	88	17	75
**Bergmans**	2001	178	TAP	9	78	38	101
**Brun-Buisson**	1989	179	TAP	3	33	6	44
**Camus**	2005	180	TAP	53	77	53	73
**Gastinne**	1992	181	PTA	26	194	34	191
**Georges**	1994	182	PNeA	3	28	15	18
**Koeman**	2006	183	oral	16	112	23	107
**Korinek**	1993	184	TAP	20	43	37	23
**Pneumatikos**	2002	185	PTA	5	26	16	14
**Pugin**	1991	186	TAP	4	21	24	3
**Quinio**	1996	187	TAP	19	57	38	34
**Rodríguez-Roldán**	1990	188	PTNeA	0	13	11	6
**Unertl**	1987	189	PGA	1	18	9	11
**Wiener**	1995	190	PGNy	8	22	8	23

Interventions; UC – usual care; TAP = topical antibiotic prophylaxis; PPAP = protocolized parenteral antibiotic prophylaxis

Notes;

1. The control group patients in duplex studies all received protocolized parenteral antibiotic therapy [PPAP]. Nonconcurrent control groups for Bergmans, Brun-Buisson & Winter in the original publications and subtracted from the combined control group data as abstracted within the Minozzi data.
2. The abstracted data as listed in the two reviews differ. Several studies in the earlier Cochrane review [Liberatti, 199] obtained and used intention to treat data where available [Aerdts; Blair; Boland; Brun-Buisson; Cerra; Finch; Hammond; Jacobs; Lignau; Korinek; Palomar; Pugin; Rocha; Rodriguez-Rolda; Sanchez-Garcia; Verwaest; Ulrich] whereas the Minozzi [203] Cochrane review used the data as published. It is the data as listed in the Minozzi Cochrane review that is listed here so as to maintain consistency between all Cochrane review data.
3. The Lignau data as listed in Minozzi [203] has the two treatment groups combined and without early exclusions. By contrast, in Liberati [202] the data for this study lists intention to treat data with early exclusions retained for the three groups separately.
4. Patients with early mortality were not counted in the pneumonia denominator for Finch 1991, Rocha 1992, Verwaest 1992 & Bergmans 2001.
5. Verwaest, Camus (2005) and Koeman are three, four and three arm studies but only data for one (TAP) intervention group is provided in the source documents [ref 202 & ref 203].
6. VAP data for Camus is as listed in Minozzi despite major discrepancies (>10%) versus the original report.

Data provenance; from Table 1.4 & Table 2.4 of ref 202 and Table 1.2 & Table 2.2.2. of ref 203

202 Liberati A, D’Amico R, Pifferi S, Torri V, Brazzi L, Parmelli E. Antibiotic prophylaxis to reduce respiratory tract infections and mortality in adults receiving intensive care. Cochrane Database of Systematic Reviews 2009, Issue 4. Art. No.: CD000022.
203 Minozzi S, Pieri S, Brazzi L, Pecoraro V, Montrucchio G, D'Amico R. Topical antibiotic prophylaxis to reduce respiratory tract infections and mortality in adults receiving mechanical ventilation. Cochrane Database of Systematic Reviews 2021, Issue 1. Art. No.: CD000022.

Table s9: Summary of contrast based findings and contrast with Cochrane review findings [a, b]

Intervention	Median VAP incidence [c]		OR / RR; (95% CI)	n / N	source [ref]
	Control	Intervention			
**UGIT versus placebo**					
Any vs UC	143	164	RR: 1.15; 0.9 – 1.48	9/ 1331	[p4; 191]
**[Fig s1a]**			OR: 1.23; 0.88 – 1.72	15 / 1481	Fig s1a
**UGIT versus other**					
H2RA vs PPI	123	126	RR: 1.02; 0.77 – 1.35	10/1256	[p11; 191]
H2RA vs antacids	280	294	RR: 1.05; 0.81 – 1.36	4/581	[p13; 191]
H2RA vs Sucralfate	189	230	RR: 1.22; 1.07 – 1.4	17/ 3041	[p14; 191]
Antacids vs Sucralfate	232	242	RR: 1.04; 0.84 – 1.3	7/996	[p16; 191]
**Aggregate [Fig s2a]**			OR: 1.0; 0.84 – 1.19	40 / 6128	Fig s2a
**Feeding interventions**					
EN vs PN	314	268	RR: 1.1; 0.82 – 1.48	7 / 361	[p5; 192]
Post pyloric feeding	285	185	RR: 0.65; 0.51 - 0.84	9 / 819	[p3; 194]
**Aggregate [Fig s3a]**			OR: 0.72; 0.52 – 1.0	23 / 1633	Fig s3a
**Airway interventions**					
CTSS vs OTSS	NS	NS	RR: 0.88; 0.7 – 1.12	11 / 1684	[p26; 195]
Semi-recumbent vs supine	402	145	RR: 0.36; 0.25 – 0.50	8 / 759	[p4; 197]
HH vs HME	32 [d]	30 [d]	RR: 0.93; 0.73 – 1.19	13/2251	[p3; 196]
Silver ETT vs ETT	75	48	RR: 0.64; 0.43 – 0.96	1 / 1509	[p4; 198]
**Aggregate [Fig s4a]**			OR: **0.61; 0.45 – 0.82**	**35 / 5987**	Fig s4a
**Probiotic interventions**					
Probiotic	309	238	OR: **0·7; 0·52 – 0·95**	5 / 1018	[p3; 199]
**[Fig s5a]**			OR: 0.66; 0.42 – 1.05	8 / 1044	Fig s5a
**Antiseptic interventions**					
Chlorhexidine	243	180	RR: **0·75; 0·62 – 0·91**	18 / 2451	[p5; 200]
Chlorhexidine	261	175	RR: **0·67; 0·47 – 0·97**	13 / 1206	[p5; 201]
Oral hygiene	259	179	RR: **0·61; 0·41 – 0·91**	5 / 910	[p7; 201]
**Aggregate (all) [Fig s6a]**			OR: **0.48; 0.37 – 0.64**	**38 / 4850**	Fig s6a
**Antimicrobial-duplex [Fig s7a]**			OR: 0·79; 0·54 – 1·14	16 / 2359	Fig s7a
TAP + PPAP (versus PPAP)	303	248	RR: 0·82; 0·58 – 1·16	6 / 850	[p5; 203]
**Antibiotic interventions**					
TAP + PPAP	417	179	RR: **0·43; 0·35 – 0·53**	17 / 2951	[p4; 203]
TAP (alone)	324	162	RR: **0·50; 0·36 – 0·69**	13 / 1848	[p5; 203]
**Aggregate (all) [Fig s8a]**			OR: **0.30; 0.22 – 0.40**	**30 / 4807**	Fig s8a

Footnotes;

a. Abbreviations: n/N is number of participants / number of studies. Abbreviations; RR = Risk ratio; OR = odds ratio; n = number of studies; UC = usual care; NR = Not reported; effect sizes; PPAP = protocolized parenteral antibiotic prophylaxis. significantly different from null in bold.
b. Summaries derived from fewer than 4 studies not shown
c. Median is per 1000 patients as in the Cochrane review summaries
d. The median VAP incidences reported for ref 196 are as in the original but likely in error.

Table s10. Summary of arms based findings [a]

	Summary proportion %	SE	95% CI	Q	df	tau^2	I^2 %	H2	95% PI
**UGIT versus placebo (Fig s1 b & c)**									
Control (n=9)	14	1.5	7 – 27	36	8	1.13	81.1	5.3	1.1 - 71
Intervention (n=15)	18	5.0	15 – 23	180	14	1.31	89.6	9.7	1.6 - 74
**UGIT versus other (Fig s2 b & c)**									
Control (n=39)	18	4.9	15 – 22	171	38	.44	82.2	5.6	5.5 - 47
Intervention (n=40)	19	5.0	15 – 23	180	39	.49	82.8	5.8	5.2 - 50
**Feeding interventions (Fig s3 b & c)**									
Control (n = 23)	31	7.1	25 – 38	70	22	.349	69.4	3.3	11 - 61
Intervention (n = 23)	25	7.8	19 – 32	82	22	.517	75.1	4.0	6.5 - 60
**Airway interventions (Fig s4 b & c)**									
Control (n = 36)	25	7.3	19 – 32	305	35	.85	90	9.9	4.7 - 69
Intervention (n=36)	17	5.1	13 – 21	186	35	.47	80.5	5.1	4.6 - 45
**Probiotic interventions (Fig s5 b & c)**									
Control (n = 8)	31	14.3	20 – 45	51	7	.65	88.6	8.8	5.1 - 79
Intervention (n=8)	22	1.1	15 – 33	27.8	7	.40	78.3	4.6	5.1 - 61

Table s10 (continued). Summary of arms-based findings [a]

	Summary proportion %	SE	95% CI	Q	df	tau^2	I^2 %	H2	95% PI
**Antiseptic interventions (Fig s6 b & c)**									
Control (n=36)	34	8.3	26 – 42	298	35	.85	90	11.3	6.2 - 79
Intervention (n=39)	19	5.4	16 – 24	235	38	.57	83.5	6.0	4.8 - 53
**Antimicrobial-duplex (Fig s7 b & c)**									
Control (n=16)	20	8.2	14 – 27	87	15	.491	83.5	6.0	4.9 - 54
Intervention (n=16)	16	7.9	12 – 23	108	15	.543	83.3	6.0	3.7 - 51
**Antibiotic interventions (Fig s8 b & c)**									
Control (n = 30)	41	8.4	14 - 27	301	29	.762	92.4	13.2	10 - 81
Intervention (n = 30)	17	5.3	13 – 21	155	29	.40	81.6	5.4	5.1 - 43
**Antimicrobial interventions (low pneumonia) (Fig s9 a)**									
Control (n = 31)	21	4.1	18 – 24	98.9	30	.21	71.7	3.5	8.9 - 40
Intervention (n = 33)	13	4.3	11 – 17	113	32	.36	75.2	4.0	4.2 – 35
**Antimicrobial interventions (hi pneumonia) (Fig s9 b)**									
Control (n = 34)	54	3.8	50 – 58	79.3	33	.096	57.9	2.4	38 - 69
Intervention (n = 35)	25	5.1	21 – 29	134	34	.32	78.5	4.7	9.2 - 52

Footnotes to Table s10

a. Summary proportions were derived by pooling the logit transformed study proportions using the Stata command “meta esize’ with the ‘logitprop’ option and then with back-transformation to percentages.

References

1. Apte NM, Karnad DR, Medhekar TP, Tilve GH, Morye S, Bhave GG. Gastric colonization and pneumonia in intubated critically ill patients receiving stress ulcer prophylaxis: a randomized, controlled trial. Crit Care Med 1992;20(5):590-3.
2. Ben-Menachem T, Fogel R, Patel RV, Touchette M, Zarowitz BJ, et al. Prophylaxis for stress-related gastric hemorrhage in the medical intensive care unit. A randomized, controlled, single-blind study. Ann Intern Med 1994;121(8):568-75.
3. Eddleston JM, Pearson RC, Holland J, Tooth JA, Vohra A, Doran BH. Prospective endoscopic study of stress erosions and ulcers in critically ill adult patients treated with either sucralfate or placebo. Crit Care Med 1994;22(12):1949-54.
4. Hanisch EW, Encke A, Naujoks F, Windolf J. A randomized, double-blind trial for stress ulcer prophylaxis shows no evidence of increased pneumonia. American J Surgery 1998;176(5):453-7.
5. Kantorova I, Svoboda P, Scheer P, Doubek J, Rehorkova D, Bosakova H, et al. Stress ulcer prophylaxis in critically ill patients: a randomized controlled trial. Hepato-gastroenterology 2004;51(57):757-61.
6. Karlstadt RG, Iberti TJ, Silverstein J, Lindenberg L, Bright-Asare P, Rockhold F, et al. Comparison of cimetidine and placebo for the prophylaxis of upper gastrointestinal bleeding due to stress-related gastric mucosal damage in the intensive care unit. J Intens Care Med 1990;5(1):26-32.
7. Martin LF, Booth FV, Karlstadt RG, Silverstein JH, Jacobs DM, Hampsey J, et al. Continuous intravenous cimetidine decreases stress-related upper gastrointestinal hemorrhage without promoting pneumonia. Crit Care Med 1993;21(1):19-30.
8. Metz CA, Livingston DH, Smith JS, Larson GM, Wilson TH. Impact of multiple risk factors and ranitidine prophylaxis on the development of stress-related upper gastrointestinal bleeding: a prospective, multicenter, double-blind, randomized trial. The Ranitidine Head Injury Study Group. Crit Care Med 1993;21(12):1844-9.
9. Yildizdas D, Yapicioglu H, Yilmaz HL. Occurrence of ventilator-associated pneumonia in mechanically ventilated pediatric intensive care patients during stress ulcer prophylaxis with sucralfate, ranitidine, and omeprazole. J Crit Care 2002;17:240-5.
10. Behrens R, Hofbeck M, Singer H, Scharf J, Rupprecht T. Frequency of stress lesions of the upper gastrointestinal tract in paediatric patients after cardiac surgery: effects of prophylaxis. British Heart J. 1994;72(2):186-9.
11. Bonten MJ, Gaillard CA, van der Geest S, van Tiel FH, Beysens AJ, Smeets HG, et al. The role of intragastric acidity and stress ulcers prophylaxis on colonization and infection in mechanically ventilated ICU patients. A stratified, randomized, double-blind study of sucralfate versus antacids. Am J Respir Crit Care Med 1995;152(6 Pt 1):1825-34.
12. Cioffi WG, McManus AT, Rue LW, Mason AD, McManus WF, Pruitt BA. Comparison of acid neutralizing and non-acid neutralizing stress ulcer prophylaxis in thermally injured patients. J Trauma1994;36(4):544-7.
13. Conrad SA, Gabrielli A, Margolis B, Quartin A, Hata JS, Frank WO, et al. Randomized, double-blind comparison of immediate-release omeprazole oral suspension versus intravenous cimetidine for the prevention of upper gastrointestinal bleeding in critically ill patients. Crit Care Med 2005;33(4):760-5.
14. Cook D, Guyatt G, Marshall J, Leasa D, Fuller H, Hall R, et al. A comparison of sucralfate and ranitidine for the prevention of upper gastrointestinal bleeding in patients requiring mechanical ventilation. Canadian Critical Care Trials Group. N Engl J Med 1998;338(12):791-7.
15. De Azevedo JR, Soares MD, Silva GA, De Lima PG. Prevention of stress ulcer bleeding in high risk patients. Comparison of three drugs. Gastroenterologia Endoscopia Digestiva 2000;19(6):239-44.
16. Driks MR, Craven DE, Celli BR, Manning M, Burke RA, Garvin GM, et al. Nosocomial pneumonia in intubated patients given sucralfate as compared with antacids or histamine type 2 blockers. The role of gastric colonization. N Engl J Med 1987;317(22):1376-82.
17. Eddleston JM, Vohra A, Scott P, Tooth JA, Pearson RC, McCloy RF, et al. A comparison of the frequency of stress ulceration and secondary pneumonia in sucralfate- or ranitidine-treated intensive care unit patients. Crit Care Med 1991;19(12):1491-6.
18. Ephgrave KS, Kleiman-Wexler R, Pfaller M, Booth BM, Reed D, Werkmeister L, et al. Effects of sucralfate vs antacids on gastric pathogens: results of a double-blind clinical trial. Archives of Surgery 1998;133(3):251-7
19. Fabian TC, Boucher BA, Croce MA, Kuhl DA, Janning SW, Coffey BC, et al. Pneumonia and stress ulceration in severely injured patients. A prospective evaluation of the effects of stress ulcer prophylaxis. Archives of Surgery 1993;128(2):185-92.
20. Fogas JF, Kiss KK, Gyura FG, Tobias ZT, Molnar ZM. Eects of proton pump inhibitor versus H2-receptor antagonist stress ulcer prophylaxis on ventilator-associated pneumonia: A pilot study. Critical Care 2013;17:S150-1.
21. Kappstein I, Schulgen G, Friedrich T, Hellinger P, Benzing A, Geiger K, et al. Incidence of pneumonia in mechanically ventilated patients treated with sucralfate or cimetidine as prophylaxis for stress bleeding: bacterial colonization of the stomach. Am J Med 1991;91(2A):125S-31S.
22. Khorvash F, Abbasi S, Meidani M, Dehdashti F, Ataei B. The comparison between proton pump inhibitors and sucralfate in incidence of ventilator associated pneumonia in critically ill patients. Advanced Biomedical Research 2014;3(52):1-6.

23. Laggner AN, Lenz K, Base W, Druml W, Schneeweiss B, Grimm G. Prevention of upper gastrointestinal bleeding in long-term ventilated patients. Sucralfate versus ranitidine. Am J Med 1989;86(6A):81-4.

24. Lee TH, Hung FM, Yang LH. Comparison of the efficacy of esomeprazole and famotidine against stress ulcers in a neurosurgical intensive care unit. *Adv Dig Med* 2014;**1**(2):50-3.

25. Levy MJ, Seelig CB, Robinson NJ, Ranney JE. Comparison of omeprazole and ranitidine for stress ulcer prophylaxis. Digestive Diseases and Sciences 1997;42(6):1255-9.

26. Lin CC, Hsu YL, Chung CS, Lee TH. Stress ulcer prophylaxis in patients being weaned from the ventilator in a respiratory care center: a randomized control trial. *J Formosan Medi Assoc* 2016;**115**(1):19-24.

27. Mahul P, Auboyer C, Jospe R, Ros A, Guerin C, el Khouri Z, et al. Prevention of nosocomial pneumonia in intubated patients: respective role of mechanical subglottic secretions drainage and stress ulcer prophylaxis. Inten Care Med 1992;18(1):20-5.

28. Maier RV, Mitchell D, Gentilello L. Optimal therapy for stress gastritis. Annals of Surgery 1994;220(3):353-60.

29. Martin LF, Max MH, Polk HC Jr. Failure of gastric pH control by antacids or cimetidine in the critically ill: a valid sign of sepsis. Surgery 1980;88(1):59-68.

30. Mustafa NA, Akturk G, Ozen I, Koksal I, Erciyes N, Solak M. Sucralfate versus ranitidine prophylaxis in intensive care patients. Turkish J Medical Sciences1994; Vol. 22, issue 2:103-6.

31. Phillips JO, Metzler MH, Huckfeldt RE, et al. A multicenter,prospective, randomised clinical trial of continuous I.V.ranitidine vs. Omeprazole suspension in the prophylaxis of stress ulcer prophylaxis (abstract). Crit Care Med. 1998; 26, A101.

32. Pickworth_KK, Falcone_RE, Hoogeboom_JE, Santanello_SA. Occurrence of nosocomial pneumonia in mechanically ventilated trauma patients: a comparison of sucralfate and ranitidine. *Crit Care Med.* 1993;**21**(12):1856-62.

33. Prakash S, Rai A, Gogia RA. Nosocomial pneumonia in mechanically ventilated patients receiving ranitidine or sucralfate as stress ulcer prophylaxis. Indian J Anaesthesia 2008;52(2):179-84.

34. Prod'hom G, Leuenberger P, Koerfer J, Blum A, Chiolero R, Schaller MD, et al. Nosocomial pneumonia in mechanically ventilated patients receiving antacid, ranitidine, or sucralfate as prophylaxis for stress ulcer. A randomized controlled trial. Ann Intern Med 1994;120(8):653-62.

35. Ryan P, Dawson J, Teres D, Celoria G, Navab F. Nosocomial pneumonia during stress ulcer prophylaxis with cimetidine and sucralfate. Archives of Surgery 1993;128(12):1353-7.

36. Simms HH, DeMaria E, McDonald L, Peterson D, Robinson A, Burchard KW. Role of gastric colonization in the development of pneumonia in critically ill trauma patients: results of a prospective randomized trial. *J Trauma* 1991;**31**(4):531-6; discussion 536-7.

37. Sirvent JM, Verdaguer R, Ferrer MJ, Avila FJ, Diaz-Prieto A, Carratala J. Mechanical ventilation-associated pneumonia and the prevention of stress ulcer. A randomized clinical trial of antacids and ranitidine versus sucralfate. *Medicina Clinica* 1994;**102**(11):407-11.

38. Solouki M, Marashian SM, Kouchak M, Mokhtari M, Nasiri E. Comparison between the preventive effects of ranitidine and omeprazole on upper gastrointestinal bleeding among ICU patients. Tanaffos 2009;8(4):37-42.

39. Somberg L, Morris J, Fantus R, Graepel J, Field BG, Lynn R, et al. Intermittent intravenous pantoprazole and continuous cimetidine infusion: effect on gastric pH control in critically ill patients at risk of developing stress-related mucosal disease. J Trauma 2008;64(5):1202-10.

40. Thomason MH, Payseur ES, Hakenewerth AM, Norton HJ, Mehta B, Reeves TR, et al. Nosocomial pneumonia in ventilated trauma patients during stress ulcer prophylaxis with sucralfate, antacid, and ranitidine. J Trauma1996;41(3):503-8.

41. Tryba M. Risk of acute stress bleeding and nosocomial pneumonia in ventilated intensive care unit patients: sucralfate versus antacids. Am J Med 1987;83(3B):117-24.

42. Tryba M, Zevounou F, Wruck G. Stress bleeding and postoperative pneumonia in intensive care patients with ranitidine or pirenzepine [Streßblutungen und postoperative Pneumonien bei Intensivpatienten unter Ranitidin oder PirenzepinM.]. Deutsche Medizinische Wochenschrift 1988;113(23):930-6.

43. Adams S, Dellinger EP, Wertz MJ, Oreskovich MR, Simonowitz D, Johansen K. Enteral versus parenteral nutritional support following laparotomy for trauma: a randomized prospective trial. *J Trauma* 1986;**26**(10):882-91.

44. Altintas ND, Aydin K, Türkoğlu MA, Abbasoğlu O, Topeli A. Effect of enteral versus parenteral nutrition on outcome of medical patients requiring mechanical ventilation. Nutrition in Clinical Practice: Official Publication of the American Society for Parenteral and Enteral Nutrition 2011;26(3):322-9.

45. Borzotta AP, Pennings J, Papasadero B, Paxton J, Mardesic S, Borzotta R, et al. Enteral versus parenteral nutrition after severe closed head injury. J Trauma1994;37(3):459-68.

46. Fan MC, Wang QL, Fang W, Jiang Y, Li L, Sun P, et al. Early enteral combined with parenteral nutrition treatment for severe traumatic brain injury: effects on immune function, nutritional status and outcomes. *Chinese Med Sci J* 2016;**31**(4):213-20.

47. Justo Meirelles CM, Aguilar-Nascimento JE. Enteral or parenteral nutrition in traumatic brain injury: a prospective randomised trial. Nutrición Hospitalaria 2011;26(5):1120-4.

48. Kudsk KA. Gut mucosal nutritional support - enteral nutrition as primary therapy after multiple system trauma. Gut 1994;(1 Suppl):S52-4.

49. Young B, Ott L, Twyman D, Norton J, Rapp R, Tibbs P, et al. The effect of nutritional support on outcome from severe head injury. J Neurosurgery 1987;67(5):668-76.

50. Fan MC, Wang QL, Fang W, Jiang Y, Li L, Sun P, et al. Early enteral combined with parenteral nutrition treatment for severe traumatic brain injury: effects on immune function, nutritional status and outcomes. *Chinese Med Science J* 2016;**31**(4):213-20.

51. Wischmeyer PE, Hasselmann M, Kummerlen C, Kozar R, Kutsogiannis DJ, Karvellas CJ, et al. A randomized trial of supplemental parenteral nutrition in underweight and overweight critically ill patients: the TOP-UP pilot trial. Crit Care 2017;21:142.

52. Chourdakis M, Kraus MM, Tzellos T, Sardeli C, Peftoulidou M, Vassilakos D, et al. EOect of early compared with delayed enteral nutrition on endocrine function in patients with traumatic brain injury: an open-labeled randomized trial. JPEN. J Parenteral and Enteral Nutrition 2012;36(1):108-16.

53. Eyer SD, Micon LT, Konstantinides FN, Edlund DA, Rooney KA, Luxenberg MG, et al. Early enteral feeding does not attenuate metabolic response a3er blunt trauma. J Trauma1993;34(5):639-43.

54. Hill DB, Kearney P, Magnuson B, Charash W, Annis K, Mcclain C. Effects of route and timing of nutrition support in critically ill patients. Gastroenterology 2002;122(Suppl 4):A38.

55. Moses V, Mahendri NV, John G, Peter JV, Ganesh A. Early hypocaloric enteral nutritional supplementation in acute organophosphate poisoning – a prospective randomized trial. Clinical Toxicology 2009;47(5):419-24

56. Nguyen NQ, Fraser RJ, Bryant LK, Burgstad C, Chapman MJ, Bellon M, et al. The impact of delaying enteral feeding on gastric emptying, plasma cholecystokinin, and peptide YY concentrations in critically ill patients. Crit Care Med 2008;36(5):1469-74.

57. Acosta-Escribano J, Fernández-Vivas M, Grau Carmona T, Caturla-Such J, Garcia-Martinez M, et al. Gastric versus transpyloric feeding in severe traumatic brain injury: a prospective, randomized trial. Inten Care Med 2010;36(9):1532-9.

58. Day L, Stotts NA, Frankfurt A, Stralovich-Romani A, Volz M, Muwaswes M, et_al. Gastric versus duodenal feeding in patients with neurological disease. *J Neuroscience Nursing* 2001;**33**(3):148-9, 155-9.

59. Davies AR, Froomes PR, French CJ, Bellomo R, Gutteridge GA, Nyulasi I, etal. Randomized comparison of nasojejunal and nasogastric feeding in critically ill patients. Crit Care Med 2002;30(3):586-90.

60. Davies AR, Morrison SS, Bailey MJ, Bellomo R, Cooper DJ, Doig GS, et al. A multicenter, randomized controlled trial comparing early nasojejunal with nasogastric nutrition in critical illness. Crit Care Med 2012;40(8):2342-8.

61. Hsu CW, Sun SF, Lin SL, Kang SP, Chu KA, Lin CH, et al. Duodenal versus gastric feeding in medical intensive care unit patients. Crit Care Med 2009;37(6):1866-72.

62. Kortbeek JB, Haigh PI, Doig C. Duodenal versus gastric feeding in ventilated blunt trauma patients. J Trauma 1999;46:992-8.

63. Montecalvo MA,Steger KA, Farber HW, Smith BF, Dennis RC, Fitzpatrick GF, et al. Nutritional outcome and pneumonia in critical care patients randomized to gastric versus jejunal tube feedings. Crit Care Med 1992;20(10):1377-87.

64. Montejo JC, Grau T, Acosta J, Ruiz-Santana S, Planas M, García-De-Lorenzo A, et al. Multicenter, prospective, randomized, single-blind study comparing the eJicacy and gastrointestinal complications of early jejunal feeding with early gastric feeding in critically illpatients. Crit Care Med 2002;30(4):796-800.

65. White H, Sosnowski K,Tran K, Reeves A, Jones M. A randomised controlled comparison of early postpyloric versus early gastric feeding to meet nutritional targets in ventilated intensive care patients. Crit Care Med 2009;13(6):1-8.

66. Adams DH, Hughes M, Elliott TS. Microbial colonization of closed-system suction catheters used in liver transplant patients. *Intensive & Critical Care Nursing* 1997;**13**(2):72-6.

67. Combes P, Fauvage B, Oleyer C. Nosocomial pneumonia in mechanically ventilated patients, a prospective randomised evaluation of the Stericath closed suctioning system. Inten Care Med 2000;26(7):878-82.

68. Conrad SA, George RB, Romero MD, Owens MW. Comparison of nosocomial pneumonia rates in closed and open tracheal suction systems [abstract]. *Chest* 1989;**96**:Suppl:184.

69. Deppe SA, Kelly JW, Thoi LL, Chudy JH, Longfield RN, Ducey JP, et al. Incidence of colonization, nosocomial pneumonia, and mortality in critically ill patients using a Trach Care closed-suction system versus an open-suction system: prospective, randomized study. Crit Care Med 1990;18(12):1389-93.

70. Johnson KL, Kearney PA, Johnson SB, Niblett JB, MacMillan NL, McClain RE. Closed versus open endotracheal suctioning: costs and physiologic consequences. *Crit Care Med* 1994;**22**(4):658-66.

71. Lorente L, Lecuona M, Martin MM, Garcia C, Mora ML, Sierra A. Ventilator-associated pneumonia using a closed versus an open tracheal suction system. Crit Care Med 2005;33(1):115-9.

72. Lorente L, Lecuona M, Jimenez A, Mora ML, Sierra A. Tracheal suction by closed system without daily change versus open system. Inten Care Med 2006;32(4):538-44.

73. Rabitsch_W, Kostler_WJ, Fiebiger_W, Dielacher_C, Losert_H, Sherif_C, et al. Closed suctioning system reduces crosscontamination between bronchial system and gastric juices. *Anesthesia and Analgesia* 2004;**99**(3):886-92.

74. Topeli A, Harmanci A, Cetinkaya Y, Akdeniz S, Unal S. Comparison of the effect of closed versus open endotracheal suction systems on the development of ventilator-associated pneumonia. J Hosp Inf 2004;58(1):14-9.

75. Welte_T, Ziesing_S, Schulte_S, Wagner_TOF. Incidence of ventilator associated pneumonia in mechanically ventilated patients: a comparison of closed versus open endotracheal suctioning [abstract]. *Eur Resp J* 1997;**10 Suppl:**(25):319.

76. Zeitoun SS, de_Barros AL, Diccini S. A prospective, randomized study of ventilator-associated pneumonia in patients using a closed vs open suction system. *J Clin Nursing* 2003;**12**(4):484-9.

77. Alcoforado L, Paiva D, Souza da Silva F, Martins Glavúo A, Galindo Filho V, Cunha Brandúo D, et al. Heat and moisture exchanger: protection against lung infections? Pilot study [Trocador de calor e humidade: proteção contra infeções pulmonares? Estudo piloto]. *Fisioterapia e Pesquisa* 2012;**19**(1):57-62.

78. Boots R, Howe S, George N, Harris F, Faoagali J. Clinical utility of hygroscopic heat and moisture exchangers in intensive care patients. Crit Care Med 1997;25(10):1707-12.

79. Boots RJ, George N, Faoagali JL, Druery J, Dean K, Heller RF. Double-heater-wire circuits and heat-and-moisture exchangers and the risk of ventilator-associated pneumonia. Crit Care Med 2006;34(3):687-93.

80. Branson RD, Davis K Jr, Brown R, Rashkin M. Comparison of three humidification techniques during mechanical ventilation: patient selection, cost and infections considerations. *Respiratory Care* 1996;**41**(9):809-16.

81. Diaz RB, Barbosa DA, Bettencourt AR, Vianna LAC, Gir E, Guimaraes T. Evalution [sic] the use of hygroscopic humidifier filters to prevent nosocomial pneumonia [Avaliacao do uso de filtros umidificadores higroscopicos para prevencao de pneumonia hospitalar]. Acta Paulista de Enfermagem 2002;15(4):32-44.

82. Dreyfuss D, Djedaini K, Gros I, Mier L, LeBourdelles G, Cohen Y, et al. Mechanical ventilation with heated humidifiers or heat and moisture exchangers: effects on patient colonization and incidence of nosocomial pneumonia. Am J Respir Crit Care Med 1995;151(4):986-92.

83. Hurni JM, Feihl F, Lazor R, et al. Safety of combined heat and moisture exchanger filters in long-term mechanical ventilation. *Chest* 1997;111:686-91.

84. Kirton O, De Haven B, Morgan J, Morejon O, Civetta J. Rates of nosocomial pneumonia associated with HME/bacterial filter and heated wire humidifiers: a prospective, randomised trial. *International J Intensive Care* 1997;**4**(1):6-13.

85. Kollef MH, Shapiro SD, Boyd V, Silver P, Von Harz B, et al. A randomized clinical trial comparing an extended-use hygroscopic condenser humidifier with heated-water humidification in mechanically ventilated patients. Chest 1998;113:759-67.

86. Lacherade JC, Auburtin M, Cerf C, Van de Louw A, Soufir L, Rebufat Y, et al. Impact of humidification systems on ventilator-associated pneumonia: a randomized multicenter trial. Am J Respir Crit Care Med 2005;172(10):1276-82.

87. Lorente L, Lecuona M, Jimenez A, Mora ML, Sierra A. Ventilator associated pneumonia using a heated humidifier or a heat and moisture exchanger: a randomized controlled trial. *Critical Care* 2006;**10**(4):R116.

88. Martin C, Perrin G, Gevaudan MJ, Saux P, Gouin F. Heat and moisture exchangers and vaporizing humidifiers in the intensive care unit. Chest 1990;97(1):144-9.

89. Memish ZA, Oni GA, Djazmati W, Cunningham G, Mah MW. A randomized clinical trial to compare the effects of a heat and moisture exchanger with a heated humidifying system on the occurrence rate of ventilator-associated pneumonia. Am J Inf Cont 2001;29(5):301-5.

90. Roustan JP, Kienlen J, Aubas P, Aubas S, du Cailar J. Comparison of hydrophobic heat and moisture exchangers with heated humidifier during prolonged mechanical ventilation. Inten Care Med 1992;18(2):97-100.

91. Kollef MH, Afessa B, Anzueto A, Veremakis C, Kerr KM, Margolis BD, Craven DE, Roberts PR, Arroliga AC, Hubmayr RD, Restrepo MI. Silver-coated endotracheal tubes and incidence of ventilator-associated pneumonia: the NASCENT randomized trial. JAMA. 2008;300(7):805-13.

92. **Cai** Fen C. Effect of different body position on ventilation-related pneumonia [**不同体位**对呼吸机相关性肺炎的影响]. *J Nursing* 2006;**12**(04A):606-7.

93. Drakulovic MB, Torres A, Bauer TT, Nicolas JM, Nogue S, Ferrer M. Supine body position as a risk factor for nosocomial pneumonia in mechanically ventilated patients: a randomized trial. Lancet 1999;354(9193):1851-8.

94. Hang H, Gu R. Semirecumbent position for the prevention of ventilator-associated pneumonia [**半坐卧位在**预防呼吸机相关性肺炎**中的**应用]. Hu Li Shi Jian Yu Yan Jiu [Nursing Practice and Research] 2012;9(4):48-9.

95. Hu H. Posture management for the prevention of ventilator associated pneumonia [**体位**护理预防机械通气相关性肺炎的**影响**]. *Yi Xue Qian Yan [Medical Frontier]* 2012;**8**(24):240-1.

96. van Nieuwenhoven CA, Vandenbroucke-Grauls C, van Tiel FH, Joore HC, van Schijndel RJ, van der Tweel I, et al. Feasibility and effects of the semirecumbent position to prevent ventilator-associated pneumonia: a randomized study. Crit Care Med 2006;34(2):396-402.

97. Keeley L. Reducing the risk of ventilator-acquired pneumonia through head of bed elevation. Nursing in Crit Care 2007;12(6):287-94.

98. Leng Y, Yi M, Nie C. 30 degree is more appropriate for the critically ill patients receiving mechanical ventilation. Unpublished but part of results presented in a meta-analysis2012.

99. Wu H, Wei M, Wang J. Effect of different body position on ventilator-associated pneumonia [**两种体位**对呼吸机相关性肺**炎的影响**]. *Chinese J General Practice* 2009;**7**(2):148-9.

100. Xue F, Lin M. The eMects of diMerent positions for the prevention of ventilator-associated pneumonia [**不同体位在**预防呼吸 **机相关性肺炎的效果**评价]. *J Qiqihar University of Medicine* 2012;**33**(16):2261-2.

101. Yu C, Wang W. The body position management in ICU for reducing ventilator-associated pneumonia incidence [ICU**体位** 护理对减少机械通气相关性肺炎发病率的效果评价]. *Chinese J Practical Nursing* 2012;**28**(11):21-2.

102. Barraud D, Blard C, Hein F, Marcon O, Cravoisy A, Nace L, et al. Probiotics in the critically ill patient: a double blind, randomized, placebo-controlled trial. Inten Care Med 2010;36(9):1540-7.

103. Forestier C, Guelon D, Cluytens V, Gillart T, Sirot J, De Champs C. Oral probiotic and prevention of Pseudomonas aeruginosa infections: a randomized, double-blind, placebo-controlled pilot study in intensive care unit patients. *Critical Care* 2008;**12**(3):R69.

104. Klarin B, Molin G, Jeppsson B, Larsson A. Use of the probiotic Lactobacillus plantarum 299 to reduce pathogenic bacteria in the oropharynx of intubated patients: a randomised controlled open pilot study. Crit Care 2008;12(6):R136.

105. Knight D, Gardiner D, Banks A, Snape SE, Weston VC, Bengmark S, et al. Effect of synbiotic therapy on the incidence of ventilator associated pneumonia in critically ill patients: a randomised, double-blind, placebo-controlled trial. Inten Care Med 2009;35(5):854-61.

106. Kotzampassi K, Giamarellos-Bourboulis EJ, Voudouris A, Kazamias P, Eleftheriadis E. Benefits of a synbiotic formula (Synbiotic 2000Forte) in critically Ill trauma patients: early results of a randomized controlled trial. World J Surgery 2006;30(10):1848-55.

107. Morrow LE, Kollef MH, Casale TB. Probiotic prophylaxis of ventilator-associated pneumonia: a blinded, randomized, controlled trial. Am J Respir Crit Care Med 2010;182(8):1058-64.

108. Spindler-Vesel A, Bengmark S, Vovk I, Cerovic O, Kompan L. Synbiotics, prebiotics, glutamine, or peptide in early enteral nutrition: a randomized study in trauma patients. J Parenteral and Enteral Nutrition 2007;31(2):119-26.

109. Tan M, Zhu JC, Du J, Zhang LM, Yin HH. Effects of probiotics on serum levels of Th1/Th2cytokine and clinical outcomes in severe traumatic brain-injured patients: a prospective randomized pilot study. *Critical Care* 2011;**15**(R290):1-10.

110. Bellissimo-Rodrigues F, Bellissimo-Rodrigues WT, Viana JM, Teixeira GC, Nicolini E, Auxiliadora-Martins M, et al. Effectiveness of oral rinse with chlorhexidine in preventing nosocomial respiratory tract infections among intensive care unit patients. Infect Cont Hosp Epidemiol2009;30(10):952-8.

111. Berry AM, Davidson PM, Masters J, Rolls K, Ollerton R. Effects of three approaches to standardized oral hygiene to reduce bacterial colonization and ventilator associated pneumonia in mechanically ventilated patients: A randomised control trial. Internat J Nursing Studies 2011;48(6):681–8.

112. Cabov T, Macan D, Husedzinovic I, Skrlin-Subic J, Bosnjak D, Sestan-Crnek S, et al. The impact of oral health and 0.2% chlorhexidine oral gel on the prevalence of nosocomial infections in surgical intensive-care patients: a randomized placebo-controlled study. Wiener Klinische Wochenschrift 2010;122(13-14):397-404.

113. Chen QL, Ye XF, Jiang YZ, Yan MQ. Application of new oral care method to orotracheal intubation. *Fujian Med J* 2008;**30**(5):155–7.

114. DeRiso AJ, Ladowski JS, Dillon TA, Justice JW, Peterson AC. Chlorhexidine gluconate 0.12% oral rinse reduces the incidence of total nosocomial respiratory infection and nonprophylactic systemic antibiotic use in patients undergoing heart surgery. Chest 1996;109(6):1556-61.

115. Fourrier F, Cau-Pottier E, Boutigny H, Roussel-Delvallez M, Jourdain M, Chopin C. Effects of dental plaque antiseptic decontamination on bacterial colonization and nosocomial infections in critically ill patients. Inten Care Med 2000;26:1239-47.

116. Fourrier F, Dubois D, Pronnier P, Herbecq P, Leroy O, Desmettre T, et al. Effect of gingival and dental plaque antiseptic decontamination on nosocomial infections acquired in the intensive care unit: a double-blind placebo-controlled multicenter study. Crit Care Med 2005;33(8):1728-35.

117. Fu T, Zhong Q, Zheng C. Bacteriostasis effect of oral administration of chlorhexidine on patients with mechanical ventilation and prevention and treatment of ventilator - associated pneumonia. Chinese Nursing Research 2019;33(3):431-4.

118. Grap MJ, Munro CL, Hamilton VA, Elswick RK Jr, Sessler CN, Ward KR. Early, single chlorhexidine application reduces ventilator-associated pneumonia in trauma patients. *Heart & Lung* 2011;**40**(5):e115–22.

119. Jacomo AD, Carmona F, Matsuno AK, Manso PH, Carlotti AP. Effect of oral hygiene with 0.12% chlorhexidine gluconate on the incidence of nosocomial pneumonia in children undergoing cardiac surgery. Infect Cont Hosp Epidemiol 2011;32(6):591-6.

120. Koeman M, Van der Ven AJ, Hak E, Joore HC, Kaasjager K, De Smet AG, et al. Oral decontamination with chlorhexidine reduces the incidence of ventilator-associated pneumonia. Amer J Resp Crit Care Med 2006;173(12):1348-55.

121. Kusahara DM, Peterlini MA, Pedreira ML. Oral care with 0.12% chlorhexidine for the prevention of ventilator-associated pneumonia in critically ill children: Randomised, controlled and double blind trial. International J Nursing Studies 2012;49(11):1354-63.

122. Meidani M, Khorvash F, Abbasi S, Cheshmavar M, Tavakoli H. Oropharyngeal irrigation to prevent ventilator-associated pneumonia: comparing potassium permangenate with chlorhexidine. Internat J Preventive Med 2018;**9**(1):93.

123. Meinberg MC, Cheade M de F, Miranda AL, Fachini MM, Lobo SM. The use of 2% chlorhexidine gel and toothbrushing for oral hygiene of patients receiving mechanical ventilation: effects on ventilator-associated pneumonia [Uso de clorexidina 2% gel e escovacao mecanica na higiene bucal de pacientes sob ventilacao mecanica: efeitos na pneumonia associada a ventilador]. Revista Brasileira de Terapia Intensiva 2012;24(4):369-74

124. Munro CL, Grap MJ, Jones DJ, McClish DK, Sessler CN. Chlorhexidine, toothbrushing, and preventing ventilator-associated pneumonia in critically ill adults. American J Crit Care 2009;18(5):428-37.

125. Ozcaka O, Basoglu OK, Buduneli N, Tasbakan MS, Bacakoglu F, Kinane DF. Chlorhexidine decreases the risk of ventilator-associated pneumonia in intensive care unit patients: a randomized clinical trial. J Periodontal Res 2012;47(5):584-92.

126. Scannapieco FA, Yu J, Raghavendran K, Vacanti A, Owens SI, Wood K, et al. A randomized trial of chlorhexidine gluconate on oral bacterial pathogens in mechanically ventilated patients. Crit Care 2009;13(4):R117.

127. Sebastian MR, Lodha R, Kapil A, Kabra SK. Oral mucosal decontamination with chlorhexidine for the prevention of ventilator-associated pneumonia in children - a randomized, controlled trial. Pediatric Crit Care Med 2012;13(5):e305-10.

128. Tantipong H, Morkchareonpong C, Jaiyindee S, Thamlikitkul V. Randomized controlled trial and meta-analysis of oral decontamination with 2% chlorhexidine solution for the prevention of ventilator-associated pneumonia. Infect Cont Hosp Epidemiol 2008;29(2):131-6.

129. Tuon FF, Gavrilko O, Almeida S, Sumi ER, Alberto T, Rocha JL, et al. Prospective, randomised, controlled study evaluating early modification of oral microbiota following admission to the intensive care unit and oral hygiene with chlorhexidine. *J Global Antimicrob Resist* 2017;**8**:159-63.

130. Berry AM. A comparison of Listerine® and sodium bicarbonate oral cleansing solutions on dental plaque colonisation and incidence of ventilator associated pneumonia in mechanically ventilated patients: A randomised control trial. Intens Crit Care Nursing. 2013;29(5):275-81.

131. Caruso P, Denari S, Ruiz SAL, Demarzo SE, Deheinzelin D. Saline instillation before tracheal suctioning decreases the ncidence of ventilator-associated pneumonia. Crit Care Med 2009;37(1):32-8.

132. Feng S, Sun X, Chen Y. Application of different mouthwashes in oral nursing for patients with orotracheal intubation. *China Medicine and Pharmacy* 2012;**8**(2): 100–1.

133. Hu X, Chen X. Application of improved oral nursing method to orotracheal intubation. Chinese Journal of Misdiagnostics 2009;9(17):4058-9.

134. Mo ZD, Li XL, Ke JY, Wu JP, Chen XW. Analysis of risk factors in ventilator-associated pneumonia and preventive effect of oral care. Chinese Journal of Nosocomiology 2016;26(3):698-9, 705

135. Nobahar M, Razavi MR, Malek F, Ghorbani R. Effects of hydrogen peroxide mouthwash on preventing ventilator-associated pneumonia in patients admitted to the intensive care unit. Brazilian Journal of Infectious Diseases. 2016 Sep;20:444-50.

136. Pobo A, Lisboa T, Rodriguez A, Sole R, Magret M, Trefler S, et al. A randomized trial of dental brushing for preventing ventilator-associated pneumonia. Chest 2009;136(2):433-9.

137. Prendergast V, Jakobsson U, Renvert S, Hallberg IR. Effects of a standard versus comprehensive oral care protocol among intubated neuroscience ICU patients: results of a randomized controlled trial. *J Neuroscience Nursing* 2012;**44**(3):134–46.

138. Seguin P, Laviolle B, Dahyot-Fizelier C, Dumont R, Veber B, Gergaud S, et al. Effect of oropharyngeal povidone-iodine preventive oral care on ventilator-associated pneumonia in severely brain-injured or cerebral hemorrhage patients: a multicenter, randomized controlled trial. Crit Care Med 2014;42:1-8.

139. Seguin P, Tanguy M, Laviolle B, Tirel O, Malledant Y. Effect of oropharyngeal decontamination by povidone-iodine on ventilator-associated pneumonia in patients with head trauma. Crit Care Med 2006;34(5):1514-9

140. Stefanescu BM, Hétu C, Slaughter JC, O'Shea TM, Shetty AK. A pilot study of Biotene OralBalance gel for oral care in mechanically ventilated preterm neonates. Contemporary Clinical Trials 2013;35(2):33-9.Yao LY, Chang CK, Maa SH, Wang C, Chen CC. Brushing teeth with purified water to reduce ventilator-associated pneumonia. J Nursing Res 2011;19(4):289-97.

141. Tang J, Chen SL, Deng JL. EGicacy of mouth cavity irrigation in prevention of ventilator-associated pneumonia. Chinese Journal of Nosocomiology 2013;23(17):4119-21.

142. Xu J, Feng B, He L, Shen H, Chen XY. Influence of Different Oral Nursing Methods on Ventilator-associated Pneumonia and Oral Infection in the Patients Undergoing Mechanical Ventilation [J]. Journal of Nursing Science. 2007;7(22):56-7.

143. Xu HL. Application of improved oral nursing method to the prevention of ventilator-associated pneumonia. Journal of Qilu Nursing. 2008;14(19):15-6.

144. Yao LY, Chang CK, Maa SH, Wang C, Chen CC. Brushing teeth with purified water to reduce ventilator-associated pneumonia. Journal of Nursing Research. 2011 Dec 1;19(4):289-97.

145. Zhao Y. Research on application of Yikou gargle in prevention of ventilation associated pneumonia. *Chinese J Nosocomiology* 2012;**23**(22):5232–3.

146. De Lacerda Vidal_CF, Vidal AK de_L, Monteiro JG de_M, Cavalcanti_A, Henriques AP de_C, Olveira_M,, et al. Impact of oral hygiene involving toothbrushing versus chlorhexidine in the prevention of ventilator-associated pneumonia: a randomized study. BMC Infectious Diseases 2017;17:112

147. Dahiya U. Decontamination with chlorhexidine gluconate reduces the incidence of ventilator associated pneumonia. *Nursing J India* 2012;**103**:89-91.

148. Hanifi N, Masoumi M, Jamshidi MR, Faghihzadeh S. The effect of ozonated water and chlorhexidine gluconate on prevention of ventilator-associated pneumonia: a double-blind, randomized, clinical trial. Iranian Red Crescent Med J 2017;**19**(10):e60576.

149. Irani H, Sargazi G, Dahmardeh AR, Mofrad ZP. The effect of oral care with miswak versus chlorhexidine on the incidence of ventilator-associated pneumonia: a clinical trial study. *Medical- Surgical Nursing J* 2019;**8**(4):e100387.

150. Khaky B, Yazdannik A, Mahjobipoor H. Evaluating the efficacy of nanosil mouthwash on the preventing pulmonary infection in intensive care unit: a randomized clinical trial. Medicinski Arhiv 2018;**72**:206-9.

151. Long Y, Mou G, Zuo Y, lv F, Feng Q, Du J. Effect of modified oral nursing method on the patients with orotracheal intubation. J Nurses Training 2012;27(24):2290-3.

152. Lorente L, Lecuona M, Jimenez A, Palmero S, Pastor E, Lafuente N, et al. Ventilator-associated pneumonia with or without toothbrushing: a randomized controlled trial. Eur J Clin Microbiol Infect Dis 2012;31(10):2621-9.

153. Meidani M, Khorvash F, Abbasi S, Cheshmavar M, Tavakoli H. Oropharyngeal irrigation to prevent ventilator-associated pneumonia: comparing potassium permangenate with chlorhexidine. Internat J Preventive Med 2018;**9**(1):93.

154. Chaari A, Zribi E, Dammak H, Ghadoun H, Chtara K, Sfar S, et al. Does selective digestive decontamination prevent ventilator associated pneumonia in trauma patients? American J Therapeutics 2014;21:470-6.

155. Panchabhai TS, Dangayach NS, Krishnan A, Kothari VM, Karnad DR. Oropharyngeal cleansing with 0.2% chlorhexidine for prevention of nosocomial pneumonia in critically ill patients: an open-label randomized trial with 0.01% potassium permanganate as control. Chest 2009;135(5):1150-6.

156. Ferrer M, Torres A, Gonzàles J, de la Bellacasa JP, El-Ebiary M, Roca M, et al. Utility of selective digestive decontamination in mechanically ventilated patients. Ann Intern Med 1994;120:389-95.

157. Hammond JMJ, Potgieter PD, Saunders GL, Forder AA. Double blind study of selective decontamination of the digestive tract in intensive care. Lancet 1992;340:5-9.

158. Laggner AN, Tryba M, Georgopoulos A, Lenz K, Grimm G, Graninger W, et al. Oropharyngeal decontamination with gentamicin for long-term ventilated patients on stress ulcer prophylaxis with sucralfate?. Wien Klin Wochenschr 1994;106:15-19.

159. Lingnau W, Berger J, Javorsky F, Lejeune P, Mutz N, Benzer H. Selective intestinal decontamination in multiple trauma patients: prospective, controlled trials. J Trauma 1997;42:687-694.

160. Stoutenbeek CP, Van Saene HKF, Zandstra DF. Prevention of multiple organ failure by selective decontamination of the digestive tract in multiple trauma patients. In: Immune consequences of trauma, shock and sepsis. Mechanisms and therapeutic approaches eds; Faist E, Baue AE, Schildberg FW. Pabst Science Publishers, Berlin, 1996;2:1055-66.

161. Abele-Horn M, Dauber A, Bauernfeind A, Russwurm W, Seyfarth-Metzger I, Gleich P, Ruckdeschel G. Decrease in nosocomial pneumonia in ventilated patients by selective oropharyngeal decontamination (SOD). Intensive Care Med 1997;23:187-195.

162. Aerdts SJA, van Dalen R, Clasener HAL, Festen J, van Lier HJJ, Vollaard EJ. Antibiotic prophylaxis of respiratory tract infection in mechanically ventilated patients. Chest 1991;100:783-91.

163. Blair P, Rowlands BJ, Lowry K, Webb H, Amstrong P, Smilie J. Selective decontamination of the digestive tract: a stratified, randomized, prospective study in a mixed intensive care unit. Surgery 1991;110:303-10.

164. Boland JP, Sadler DL, Stewart W, Wood DJ, Zerick W, Snodgrass KR. Reduction of nosocomial respiratory tract infections in the multiple trauma patients requiring mechanical ventilation by selective parenteral and enteral antisepsis regimen (SPEAR) in the intensive care. XVII Congress of Chemotherapy,1991.

165. Cockerill FR, Muller SR, Anhalt JP, Marsh HM, Farnell MB, Mucha P, et al. Prevention of infection in critically ill patients by selective decontamination of the digestive tract. Ann Intern Med 1992;117:545-53

166. de_la Cal_MA, Cerdà_E, Garcia-Hierro_P, van_Saene_HK, Gómez- Santos_D, Negro_E, et al. Survival benefit in critically hill burned patients receiving selective decontamination of the digestive tract. A randomized, placebo-controlled, double-blind trial. *Annals of Intern Med* 1992;**117**:545-53.

167. Finch RG, Tomlinson P, Holliday M, Sole K, Stack C, Rocker G. Selective decontamination of the digestive tract (SDD) in the prevention of secondary sepsis in a medical/surgical intensive care unit. XVII International Congress Chemotherapy,1991.

168. Jacobs S, Foweraker JE, Roberts SE. Effectiveness of selective decontamination of the digestive tract (SDD) in an ICU with a policy encouraging a low gastric pH. Clin Intens Med 1992;3:52-8.

169. Kerver AJH, Rommes JH, Mevissen-Verhage EAE, Hulstaert PF, Vos A, Verhoef J, et al. Prevention of colonization and infection in critically ill patients: A prospective randomized study. Crit Care Med 1988;16:1087.

170. Krueger WA, Lenhart FP, Neeser G, Ruckdeschel G, Schreckhase H, Eissner HJ, et al. Influence of combined intravenous and topical antibiotic prophylaxis on the incidence of infections, organ dysfunctions and mortality in critically ill surgical patients. Am J Respir Crit Care Med 2002;166:1029-37.

171. Palomar M, Alvarez-Lerma F, Jorda R, Bermejo B. Prevention of nosocomial infection in mechanically ventilated patients: Selective digestive decontamination versus sucralfate. Clin Intens Care 1997;8:228-35.

172. Rocha LA, Martin MJ, Pita S, Paz J, Seco C, Margusino L, et al. Prevention of nosocomial infection in critically ill patients by selective decontamination of digestive tract. Inten Care Med 1992;18:398-404.

173. Sanchez-Garcia M, Cambronero JA, Lopez J, Cerda E, Rubio J, et al. Effectiveness and cost of selective decontamination of the digestive tract (SDD) in critically ill intubated patients. A randomized, double blind, placebo-controlled, multicentric trial.. Am J Respir Crit Care Med 1998;158:908-16.

174. Stoutenbeek CP, Van Saene HKF, Little RA, Whitehead A. The effect of selective decontamination on the digestive tract on mortality in multiple trauma patients: a multicentre randomized controlled trial. Inten Care Med 2007;33:261-70.

175. Ulrich C, Harinck-deWeerd JE, Bakker NC, Jacz K, Doornbos L, et al. Selective decontamination of the digestive tract with norfloxacin in the prevention of ICU-acquired infections: a prospective randomized study. Intensive Care Med 1989;15:424-31.

176. Verwaest C, Verhaegen J, Ferdinande P, Schets M, Van der Berghe G, Verbist L, et al. Randomized, controlled trial of selective digestive decontamination in 600 mechanically ventilated patients in a multidisciplinary intensive care unit. Crit Care Med 1997;25:63-71.

177. Winter R, Humphreys H, Pick A, MacGowan AP, Willatts SM, Speller DCE. A controlled trials of selective decontamination of the digestive tract in intensive care and its effect on nosocomial infection. J Antimicrob Chemother 1992;30:73-87.

178. Bergmans DCJJ, Bonten MJM, Gailard CA, Paling JC, van der Geest S, van Tiel FH, et al. Prevention of ventilator-associated pneumonia by oral decontamination: a prospective randomized, double blind, placebo controlled study. Am J Respir Crit Care Med 2001;164(3):382-8.

179. Brun-Buisson C, Legrand P, Rauss A, Richard C, Montravers F, Besbes M, et al. Intestinal decontamination for control of nosocomial multiresistant Gram-negative bacilli. Ann Intern Med 1989;110:873-81.

180. Camus C, Bellissant E, Sebille W, Perrotin D, Garo B, Legras A, et al. Prevention of acquired infections in intubated patients with combination of two decontaminations regimens. Crit Care Med 2005;33(2):307-14.

181. Gastinne H, Wolff M, Delatour F, Faurisson F, Chevret S. A controlled trial in intensive care units of selective decontamination of the digestive tract with nonabsorbable antibiotics. N Engl J Med 1992;326:594-9.

182. Georges B, Mazerolles M, Decun JF, Rouge P, Pomies S, Cougot P, et al. Décontamination digestive sélective résultats d'une ètude chez le polytraumatisé. Réan Urg 1994;3:621-627.

183. Koeman M, van der Ven AJ, Hak E, et al. Oral decontamination with chlorhexidine reduces the incidence of ventilator-associated pneumonia. *Am J Respir Crit Care Med* 2006;173:1348-1355

184. Korinek AM, Laisne MJ, Raskine L, Deroin V, Sanson-Lepors MJ. Selective decontamination of the digestive tract in neurosurgical care units patients: a double blind, randomized, placebo-controlled study. Crit Care Med 1993;21:1466-73.

185. Pneumatikos I, Koulouras V, Nathanail C, Goe D, Nakos G. Selective decontamination of subgloottic area in mechanically ventilated patients with multiple trauma. Inten Care Med 2002;28:432-7.

186. Pugin J, Auckenthaler R, Lew DP, Suter PM. Oropharyngeal decontamination decreases incidence of ventilator-associated pneumonia. JAMA 1991;265:2704-10.

187. Quinio B, Albanèse J, Bues-Charbit M, Viviand X, Martin C. Selective Decontamination of the digestive tract in multiple trauma patients: prospective, double blind, randomised, placebo-controlled study. Chest 1996;109:765-72.

188. Rodrìguez-Roldàn JM, Altuna-Cuesta A, Lòpez A, Carrillo A, Garcia J, Leòn J, Martìnez-Pellùs AJ. Prevention of nosocomial lung infection in ventilated patients:use of an antimicrobial pharyngeal nonabsorbable paste. Crit Care Med 1990;18:1239-42.

189. Unertl K, Ruckdeschel G, Selbmann HK, Jensen U, Forst H, Lenhart FP, et al. Prevention of colonization and respiratory infections in long term ventilated patients by local antimicrobial prophylaxis. Intensive Care Med 1987;13:106-13.

190. Wiener J, Itokazu G, Nothan C, Kabins SA, Weinstein RA. A randomized, double-blind, placebo-controlled trial of selective digestive decontamination in a medical-surgical intensive care unit. Clin Infect Dis 1995;20:861-7.

191. Toews_I, George_AT, Peter_JV, Kirubakaran_R, Fontes_LES, Ezekiel_JPB, Meerpohl_JJ. Interventions for preventing upper gastrointestinal bleeding in people admitted to intensive care units. *Cochrane Database of Systematic Reviews* 2018, Issue 6. Art. No.: CD008687.

192. Lewis SR, Schofield-Robinson OJ, Alderson P, Smith AF. Enteral versus parenteral nutrition and enteral versus a combination of enteral and parenteral nutrition for adults in the intensive care unit. Cochrane Database of Systematic Reviews 2018, Issue 6. Art. No.: CD012276.

193. Padilla PF, Martínez G, Vernooij RW, Urrútia G, i Figuls MR, Cosp XB. Early enteral nutrition (within 48 hours) versus delayed enteral nutrition (after 48 hours) with or without supplemental parenteral nutrition in critically ill adults. Cochrane Database of Systematic Reviews. 2019(10).

194. Alkhawaja S, Martin C, Butler RJ, Gwadry-Sridhar F. Post-pyloric versus gastric tube feeding for preventing pneumonia and improving nutritional outcomes in critically ill adults. Cochrane Database of Systematic Reviews. 2015(8).

195. Solà I, Benito S. Closed tracheal suction systems versus open tracheal suction systems for mechanically ventilated adult patients. Cochrane Database of Systematic Reviews 2007, Issue 4. Art. No.: CD004581.

196. Gillies D, Todd DA, Foster JP, Batuwitage BT. Heat and moisture exchangers versus heated humidifiers for mechanically ventilated adults and children. Cochrane Database of Systematic Reviews 2017, Issue 9. Art. No.: CD004711.

197. Wang L, Li X, Yang Z, Tang X, Yuan Q, Deng L, Sun X. Semi-recumbent position versus supine position for the prevention of ventilator-associated pneumonia in adults requiring mechanical ventilation. Cochrane Database of Systematic Reviews 2016, Issue 1. Art. No.: CD009946.

198. Tokmaji G, Vermeulen H, Müller MCA, Kwakman PHS, Schultz MJ, Zaat SAJ. Silver-coated endotracheal tubes for prevention of ventilator-associated pneumonia in critically ill patients. Cochrane Database of Systematic Reviews 2015, Issue 8. Art. No.: CD009201.

199. Bo L, Li J, Tao T, Bai Y, Ye X, Hotchkiss RS, Kollef MH, Crooks NH, Deng X. Probiotics for preventing ventilator-associated pneumonia. Cochrane Database of Systematic Reviews 2014, Issue 10. Art. No.: CD009066.

200. Hua F, Xie H, Worthington HV, Furness S, Zhang Q, Li C. Oral hygiene care for critically ill patients to prevent ventilator-associated pneumonia. Cochrane Database of Systematic Reviews 2016, Issue 10. Art. No.: CD008367.

201. Zhao T, Wu X, Zhang Q, Li C, Worthington HV, Hua F. Oral hygiene care for critically ill patients to prevent ventilator-associated pneumonia. Cochrane Database of Systematic Reviews 2020, Issue 12. Art. No.: CD008367.

202. Liberati A, D'Amico R, Pifferi S, Torri V, Brazzi L, Parmelli E. Antibiotic prophylaxis to reduce respiratory tract infections and mortality in adults receiving intensive care. Cochrane Database of Systematic Reviews 2009, Issue 4. Art. No.: CD000022.

203. Minozzi S, Pieri S, Brazzi L, Pecoraro V, Montrucchio G, D'Amico R. Topical antibiotic prophylaxis to reduce respiratory tract infections and mortality in adults receiving mechanical ventilation. Cochrane Database of Systematic Reviews 2021, Issue 1. Art. No.: CD000022.

Stata script [Stata version 18];

For the contrast based analysis [Non-antimicrobial interventions]

Declare data for meta-analysis

```
meta esize ivapn ivapm cvapn cvapm, studylabel(author ref)
meta forestplot if ref<110, sort(_meta_es, ascending)
transform(exp)  omarkeropts(mcolor(red)) columnopts(_weight,
format(%9.1g)) esrefline xtitle(odds ratio) xlabel(#5) title(Non-
Antimicobial) nullrefline noohetstats nonotes
```

For the DTA analysis

Generation of summary sensitivity and specificity estimates

```
metandi ivapn cvapn ivapm cvapm if Category labels==[label]
```

Generation of SROC plots [1, 2]

```
metandiplot ivapn cvapn ivapm cvapm if Category labels==[label],
xtitle(Control pneumonia %) xlab(1 "0" .8 "20" .6 "40" .4 "60" .2
"80" 0 "100") ylab(0 "0" .2 "20" .4 "40" .6 "60" .8 "80" 1 "100")
ytitle(Intervention pneumonia %)


metadta ivapn cvapn ivapm cvapm if Category labels==[label],
xtitle("Control VAP proportion") xlabel(0(0.2)1) xscale(range(0 1))
ytitle("Intervention VAP proportion") yscale(range(0 1))
ylabel(0(0.2)1, nogrid) legend(off) graphregion(color(white))
plotregion(margin(zero)) col(blue))
```

Generation of heterogeneity metrics [control groups]

Declare data for meta-analysis

```
generate cvapd = cvapn + cvapm
meta esize cvapn cvapd, esize(logitprop) studylabel(author)
```


1. Harbord RM, Whiting P. Metandi: meta-analysis of diagnostic accuracy using hierarchical logistic regression. The Stata Journal. 2009;9(2):211-29.

2. Nyaga VN, Arbyn M. Metadta: a Stata command for meta-analysis and meta-regression of diagnostic test accuracy data–a tutorial. Archives of Public Health. 2022;80(1):1-5.

Figure s1a. UGIT interventions versus placebo

UGIT interventions versus placebo

Study	Treatment Yes	Treatment No	Control Yes	Control No	Odds ratio with 95% CI	Weight (%)
Martin, 7	0	56	4	57	0.11 [0.01, 2.15]	1.3
Metz , 8	12	72	15	64	0.71 [0.31, 1.63]	16
Hanisch, 4	10	47	6	22	0.78 [0.25, 2.42]	8.7
Yildizdas, 9	16	22	6	8	0.97 [0.28, 3.35]	7.3
Hanisch, 4	10	34	6	22	1.08 [0.34, 3.39]	8.5
Yildizdas, 9	17	21	6	8	1.08 [0.31, 3.72]	7.3
Yildizdas, 9	20	22	6	8	1.21 [0.36, 4.10]	7.5
Kantorova, 5	6	63	5	70	1.33 [0.39, 4.58]	7.3
Kantorova, 5	7	64	5	70	1.53 [0.46, 5.07]	7.8
Kantorova, 5	8	64	5	70	1.75 [0.54, 5.63]	8.2
Karlstadt, 6	1	53	0	33	1.88 [0.07, 47.47]	1.1
Ben-Menachem, 2	12	88	3	47	2.14 [0.57, 7.95]	6.5
Ben-Menachem, 2	13	87	3	47	2.34 [0.64, 8.63]	6.6
Eddleston '94, 3	1	13	0	12	2.78 [0.10, 74.70]	1
Apte , 1	13	3	9	9	4.33 [0.91, 20.60]	4.6
**Overall**					1.23 [0.88, 1.72]	

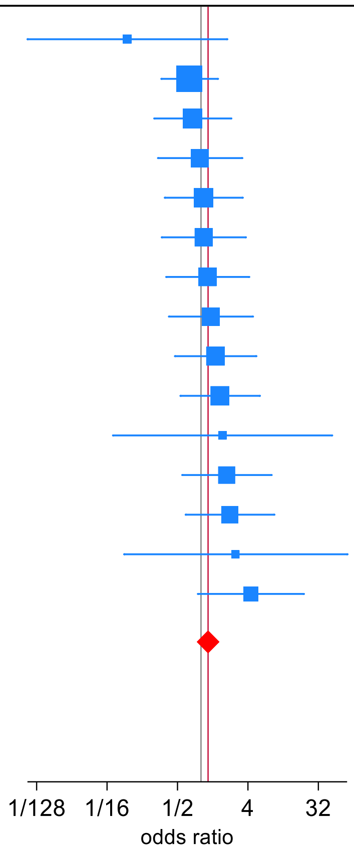

Heterogeneity: $\tau^2 = 0.00$, $I^2 = 0.00\%$, $H^2 = 1.00$

Test of $\theta_i = \theta_j$: $Q(14) = 9.97$, $p = 0.76$

Test of $\theta = 0$: $z = 1.23$, $p = 0.22$

Figure s1 b&c. UGIT interventions versus placebo. SROC's derived using 'metandi' (b) and 'metadta' (c)

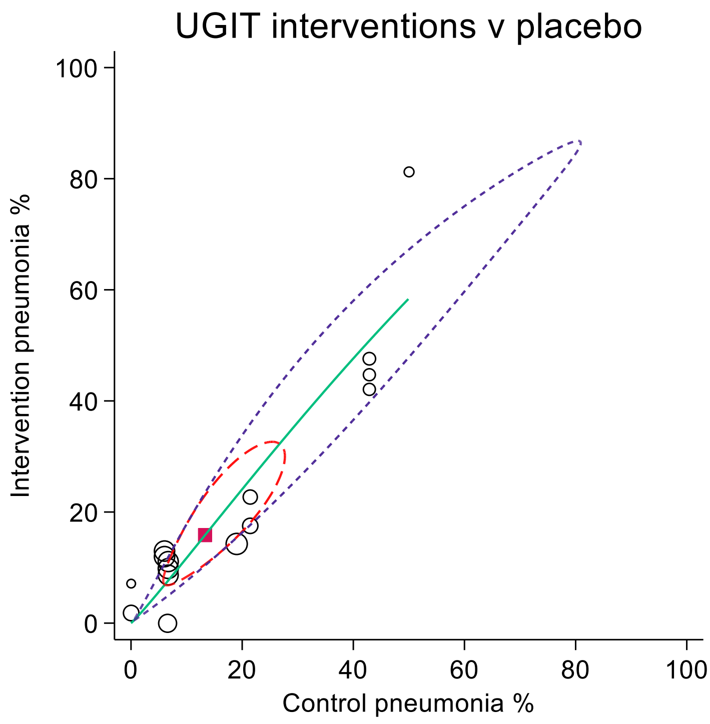

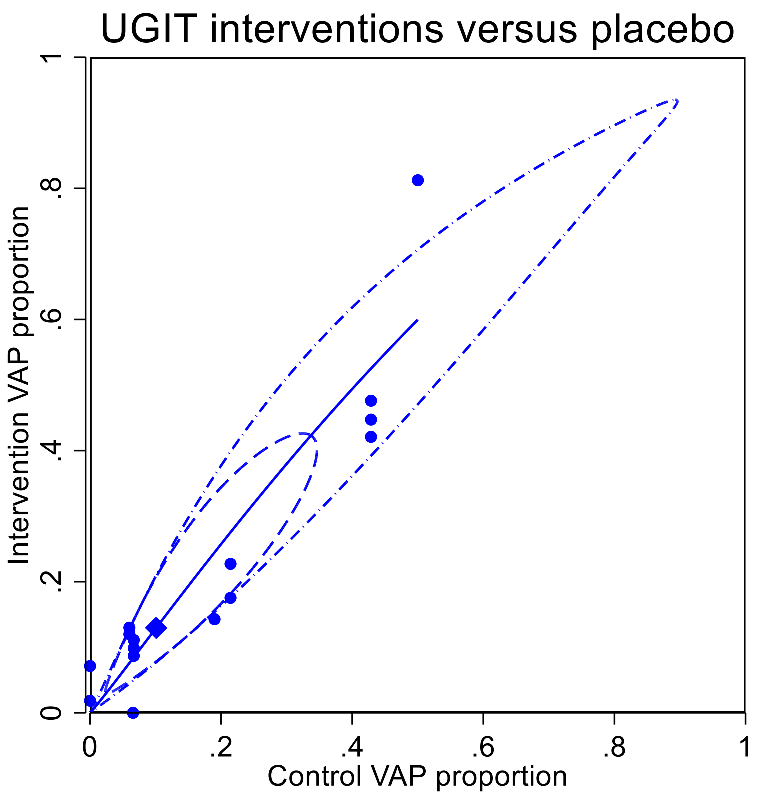

Figure s2a. UGIT interventions versus other

UGIT interventions versus other

Study	Treatment Yes	Treatment No	Control Yes	Control No	Odds ratio with 95% CI	Weight (%)
Tryba, 41	3	26	11	21	0.22 [0.05, 0.89]	1.4
Eddleston '91, 17	3	27	10	20	0.22 [0.05, 0.91]	1.4
Khorvash, 22	10	61	24	42	0.29 [0.12, 0.66]	3.2
Behrens, 10	0	22	1	20	0.30 [0.01, 7.88]	.28
Prod'hom, 34	10	73	21	59	0.38 [0.17, 0.88]	3.2
Cioffi, 12	9	41	17	33	0.43 [0.17, 1.08]	2.7
Solouki, 38	6	62	8	53	0.64 [0.21, 1.97]	2
Ephgrave, 18	11	59	15	55	0.68 [0.29, 1.62]	3.1
Pickworth , 32	5	39	6	33	0.71 [0.20, 2.52]	1.6
Fabian, 19	15	50	52	127	0.73 [0.38, 1.42]	4.4
Prod'hom, 34	18	63	21	59	0.80 [0.39, 1.65]	3.9
Conrad, 13	17	164	20	158	0.82 [0.41, 1.62]	4.2
Somberg, 39	3	32	16	151	0.88 [0.24, 3.22]	1.6
Ryan_C, 35	7	49	8	50	0.89 [0.30, 2.65]	2.1
Thomason, 40	42	94	49	99	0.90 [0.55, 1.49]	6
Simms_S , 36	8	22	9	23	0.93 [0.30, 2.84]	2
Bonten '95, 11	16	58	15	52	0.96 [0.43, 2.12]	3.4
Fogas, 20	9	29	10	31	0.96 [0.34, 2.70]	2.3
Lee, 24	1	29	1	29	1.00 [0.06, 16.76]	.38
Thomason, 40	42	94	43	97	1.01 [0.60, 1.68]	5.8
Ben-Menachem, 2	13	87	12	88	1.10 [0.47, 2.53]	3.2
Yildizdas, 9	20	22	17	21	1.12 [0.47, 2.71]	3
Kantorova, 5	7	64	6	63	1.15 [0.37, 3.61]	2
Cook, 14	114	482	98	506	1.22 [0.91, 1.64]	8.6
de Azevedo, 15	5	33	4	34	1.29 [0.32, 5.22]	1.4
Hanisch, 4	10	34	10	47	1.38 [0.52, 3.69]	2.5
Maier, 28	14	37	10	37	1.40 [0.55, 3.55]	2.7
Mahul, 27	17	55	13	60	1.43 [0.63, 3.21]	3.3
de Azevedo, 15	5	33	3	29	1.46 [0.32, 6.67]	1.2
Sirvent, 37	11	14	9	17	1.48 [0.48, 4.59]	2
Lin, 26	6	54	4	56	1.56 [0.42, 5.82]	1.5
Martin, 29	12	28	7	30	1.84 [0.63, 5.33]	2.2
Tryba, 42	6	194	3	197	2.03 [0.50, 8.24]	1.4
Prakash, 33	15	10	10	15	2.25 [0.73, 6.98]	2
Driks, 16	16	53	7	54	2.33 [0.89, 6.12]	2.6
Kappstein, 21	25	30	12	37	2.57 [1.11, 5.95]	3.2
Mustafa, 30	9	7	3	12	5.14 [1.03, 25.60]	1.1
Levy, 25	5	30	1	31	5.17 [0.57, 46.86]	.6
Phillips, 31	4	21	0	33	14.02 [0.72, 273.76]	.34
**Overall**					1.00 [0.84, 1.19]	

Heterogeneity: $\tau^2 = 0.07$, $I^2 = 25.96\%$, $H^2 = 1.35$

Test of $\theta_i = \theta_j$: Q(38) = 55.69, p = 0.03

Test of $\theta = 0$: z = -0.04, p = 0.97

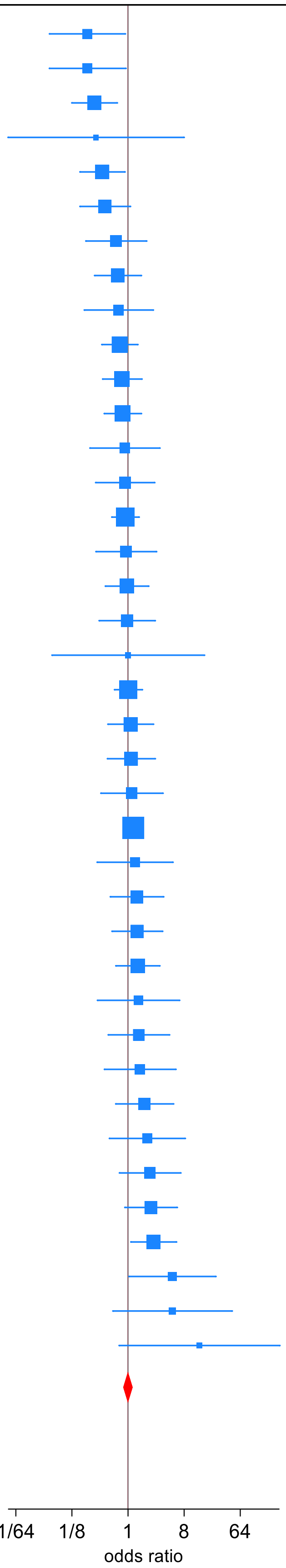

Figure s2 b&c. UGIT interventions versus other. SROC's derived using 'metandi' (b) and 'metadta' (c).

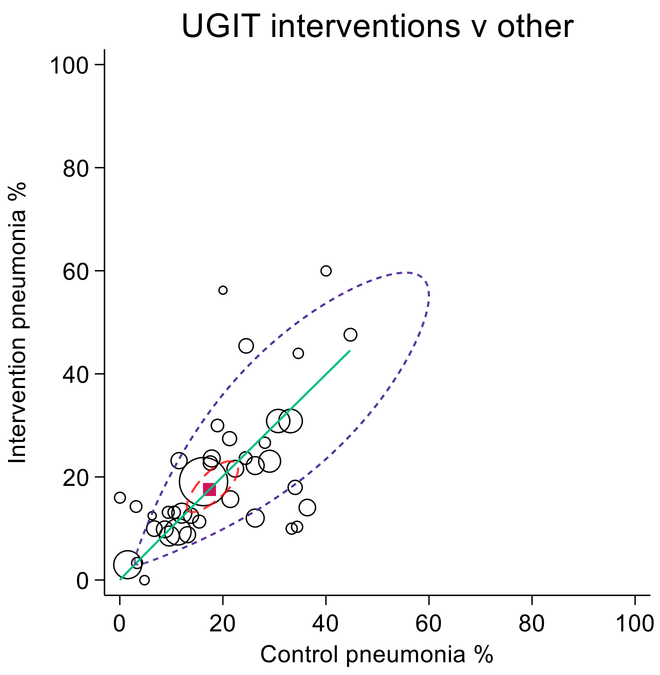

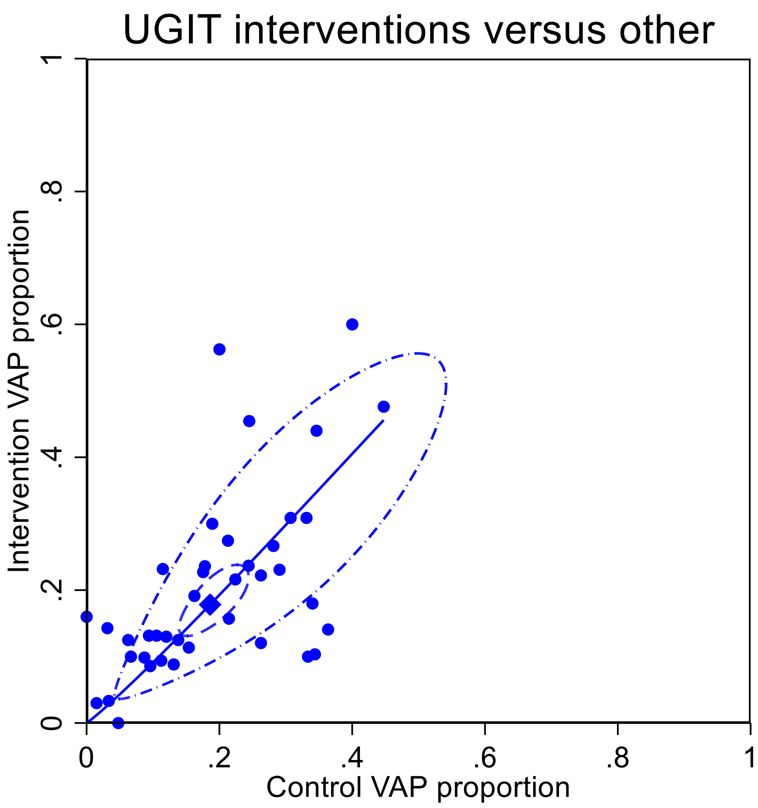

Figure s3a. Feeding interventions

Feeding interventions

Study	Treatment Yes	Treatment No	Control Yes	Control No	Odds ratio with 95% CI	Weight (%)
Montecalvo_G, 63	0	19	2	17	0.18 [0.01, 4.00]	1
Hsu, 60	5	54	15	47	0.29 [0.10, 0.86]	5
Kudsk, 48	6	45	14	31	0.30 [0.10, 0.85]	5.1
Day, 57	1	13	2	9	0.35 [0.03, 4.42]	1.4
Acosta-escribano, 56	16	34	31	23	0.35 [0.16, 0.78]	6.7
Chourdakis, 51	20	14	20	5	0.36 [0.11, 1.18]	4.5
Nguyen, 55	3	11	6	8	0.36 [0.07, 1.91]	2.9
Eyer, 52	4	15	8	11	0.37 [0.09, 1.53]	3.5
Hill, 53	4	18	9	15	0.37 [0.09, 1.45]	3.8
White, 65	5	45	11	43	0.43 [0.14, 1.35]	4.8
Kortbeek , 62	10	27	18	25	0.51 [0.20, 1.32]	5.8
Altintas, 44	5	25	11	30	0.55 [0.17, 1.78]	4.5
Montejo, 64	16	34	20	31	0.73 [0.32, 1.65]	6.6
JustoMeirelles, 47	2	10	2	8	0.80 [0.09, 7.00]	1.9
Davies, 59	18	73	19	70	0.91 [0.44, 1.87]	7.3
Wischmeyer, 50	18	55	12	40	1.09 [0.47, 2.52]	6.5
Young, 49	9	19	6	17	1.34 [0.40, 4.56]	4.4
Moses, 54	12	17	10	20	1.41 [0.49, 4.07]	5.1
Borzotta, 45	15	13	9	12	1.54 [0.49, 4.81]	4.7
Kearns_I, 61	4	17	3	20	1.57 [0.31, 8.01]	2.9
Adams, 43	11	12	8	15	1.72 [0.53, 5.62]	4.5
Davies, 58	2	29	1	34	2.34 [0.20, 27.20]	1.5
Fan, 46	20	20	8	32	4.00 [1.48, 10.79]	5.5
**Overall**					0.72 [0.52, 1.00]	

Heterogeneity: $\tau^2 = 0.24$, $I^2 = 41.60\%$, $H^2 = 1.71$

Test of $\theta_i = \theta_j$: $Q(22) = 35.74$, $p = 0.03$

Test of $\theta = 0$: $z = -1.99$, $p = 0.05$

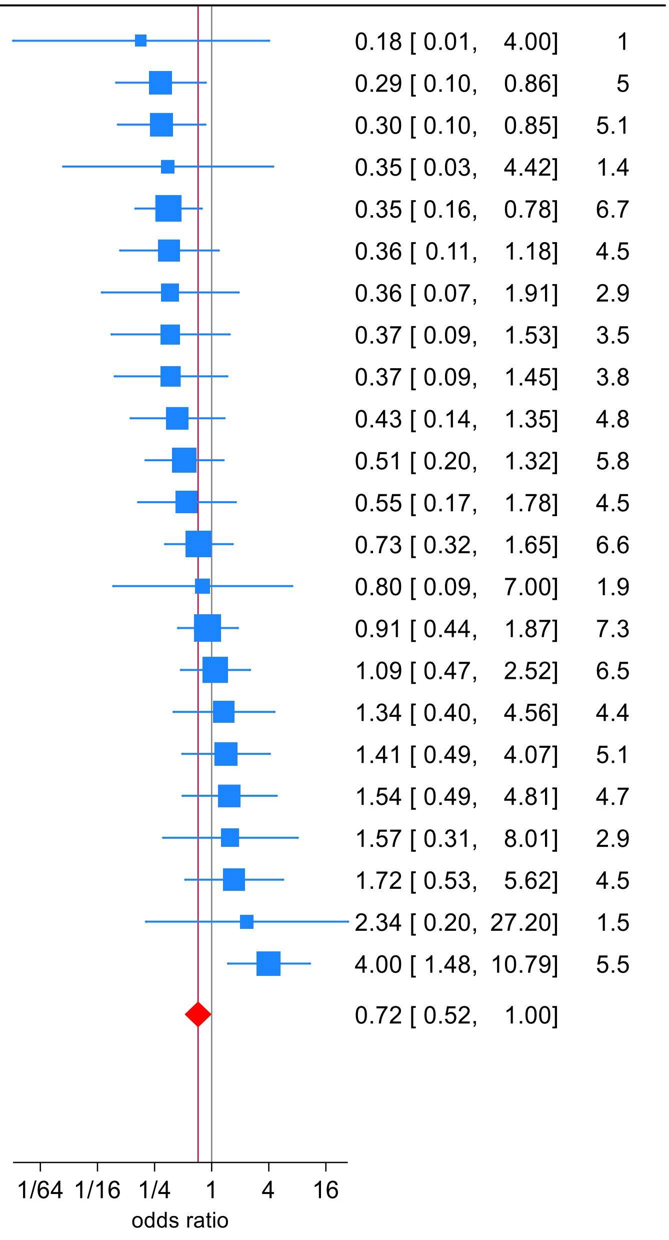

Figure s3 b&c. Feeding interventions. SROC's derived using 'metandi' (b) and 'metadta' (c).

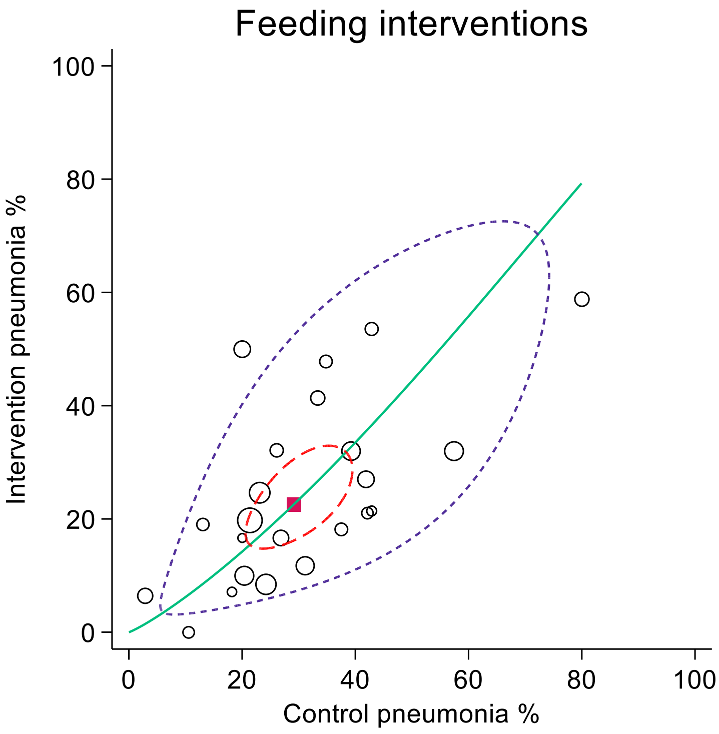

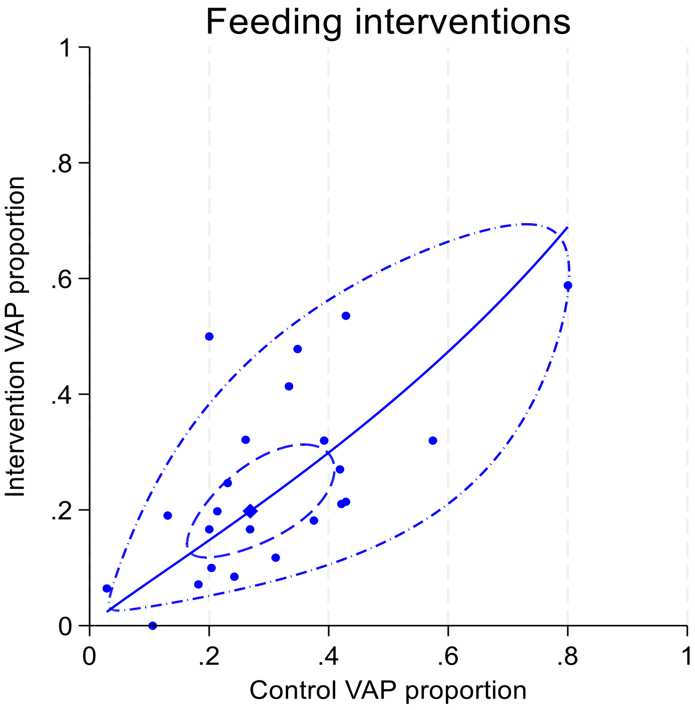

Figure s4a. Airway interventions

Airway interventions

Study	Treatment Yes	Treatment No	Control Yes	Control No	Odds ratio with 95% CI	Weight (%)
Wu, 99	11	45	48	8	0.04 [0.02, 0.11]	3.1
Rabitsch, 73	0	12	5	7	0.05 [0.00, 1.13]	.79
Drakulovic, 93	3	36	16	31	0.16 [0.04, 0.61]	2.4
Cai, 92	4	23	13	14	0.19 [0.05, 0.69]	2.5
Hang, 94	3	17	9	10	0.20 [0.04, 0.90]	2.1
Yu, 101	5	28	14	18	0.23 [0.07, 0.75]	2.7
Hu, 95	8	35	21	22	0.24 [0.09, 0.63]	3.1
Xue, 100	4	44	12	36	0.27 [0.08, 0.92]	2.6
Welte, 75	9	18	16	9	0.28 [0.09, 0.88]	2.8
Martin, 88	2	29	8	34	0.29 [0.06, 1.49]	1.9
Keeley, 97	5	12	7	6	0.36 [0.08, 1.62]	2.1
Combes, 67	4	50	9	41	0.36 [0.10, 1.27]	2.6
Kirton , 84	10	130	22	118	0.41 [0.19, 0.91]	3.5
Zeitoun, 76	7	16	11	13	0.52 [0.16, 1.71]	2.7
Roustan, 90	5	50	9	52	0.58 [0.18, 1.84]	2.7
Kollef'08_silverETT, 91	37	729	56	687	0.62 [0.41, 0.96]	4.3
Hurni, 83	5	54	7	49	0.65 [0.19, 2.18]	2.6
Memish, 89	14	109	19	101	0.68 [0.33, 1.43]	3.7
Boots, 78	6	36	7	34	0.81 [0.25, 2.65]	2.7
Lacherade '05, 86	47	138	53	131	0.84 [0.53, 1.33]	4.3
Dreyfuss, 82	6	55	8	62	0.85 [0.28, 2.59]	2.8
Deppe, 69	12	34	11	27	0.87 [0.33, 2.27]	3.2
Kollef '98, 85	15	148	15	132	0.89 [0.42, 1.89]	3.6
Johnson, 70	8	8	10	9	0.90 [0.24, 3.41]	2.4
Lorente'06a, 72	33	203	31	190	1.00 [0.59, 1.69]	4.1
Adams, 66	0	10	0	10	1.00 [0.02, 55.27]	.48
Conrad, 68	6	10	6	11	1.10 [0.27, 4.55]	2.3
Branson, 80	3	46	3	51	1.11 [0.21, 5.77]	1.9
Lorente'05, 71	43	167	42	191	1.17 [0.73, 1.88]	4.2
Leng, 98	5	23	5	28	1.22 [0.31, 4.73]	2.4
Boots, 79	32	158	27	164	1.23 [0.70, 2.15]	4.1
Alcoforado, 77	5	3	4	3	1.25 [0.16, 9.92]	1.4
Topeli, 74	13	28	9	28	1.44 [0.53, 3.92]	3.1
Diaz, 81	8	15	5	15	1.60 [0.42, 6.03]	2.4
van Nieuwenhoven, 96	13	99	8	101	1.66 [0.66, 4.17]	3.2
Lorente'06b, 87	21	32	8	43	3.53 [1.39, 8.98]	3.2
**Overall**					0.61 [0.45, 0.82]	

Heterogeneity: $\tau^2 = 0.47$, $I^2 = 68.69\%$, $H^2 = 3.19$

Test of $\theta_i = \theta_j$: $Q(35) = 95.14$, $p = 0.00$

Test of $\theta = 0$: $z = -3.32$, $p = 0.00$

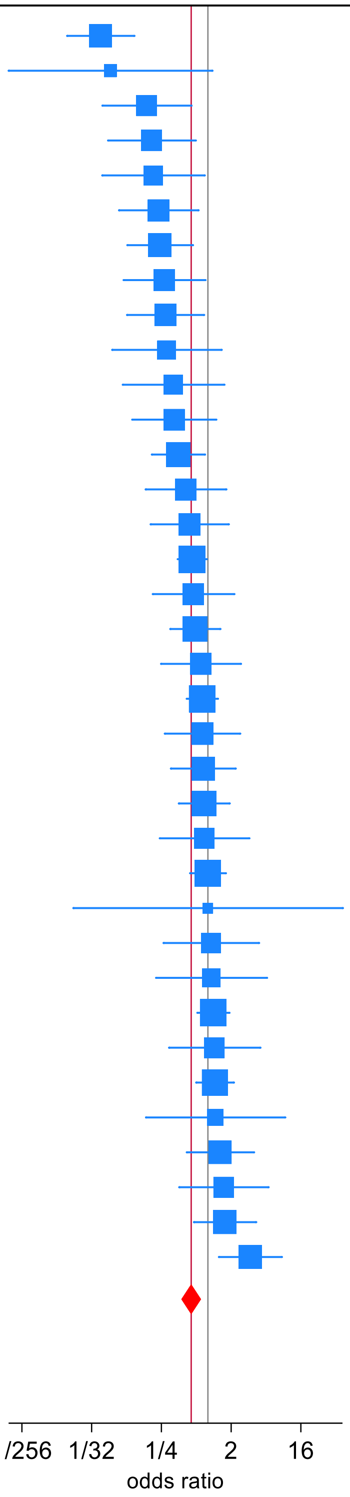

Figure s4 b&c. Airway interventions. SROC's derived using 'metandi' (b) and 'metadta' (c).

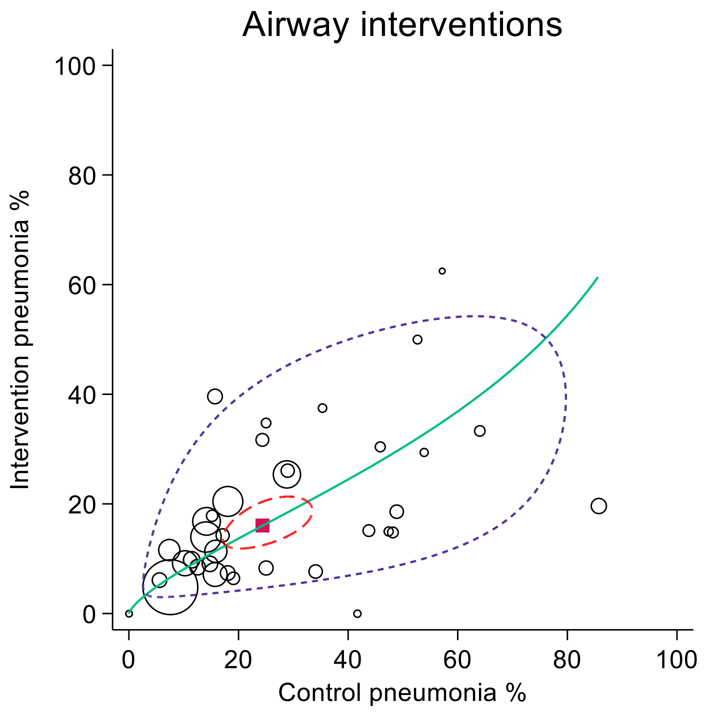

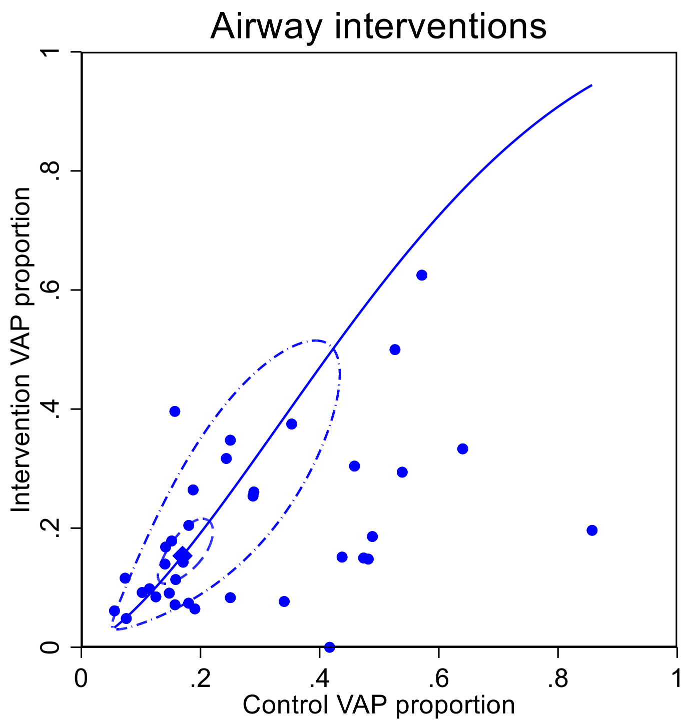

Figure s5a Probiotic interventions

Probiotic

Study	Treatment Yes	Treatment No	Control Yes	Control No	Odds ratio with 95% CI	Weight (%)
Klarin, 104	1	22	3	18	0.27 [0.03, 2.85]	3.4
Spindler-Vesel, 108	4	22	34	53	0.28 [0.09, 0.89]	10
Tan, 109	7	9	13	6	0.36 [0.09, 1.43]	7.9
Morrow, 107	17	56	33	40	0.37 [0.18, 0.75]	17
Knight, 105	12	118	17	112	0.67 [0.31, 1.47]	15
Kotzampassi, 106	15	21	16	20	0.89 [0.35, 2.27]	13
Forestier, 103	24	78	24	82	1.05 [0.55, 2.00]	18
Barraud, 102	23	64	15	65	1.56 [0.75, 3.25]	16
**Overall**					0.66 [0.42, 1.05]	

Heterogeneity: $\tau^2 = 0.20$, $I^2 = 49.16\%$, $H^2 = 1.97$

Test of $\theta_i = \theta_j$: Q(7) = 13.34, p = 0.06

Test of $\theta = 0$: z = -1.76, p = 0.08

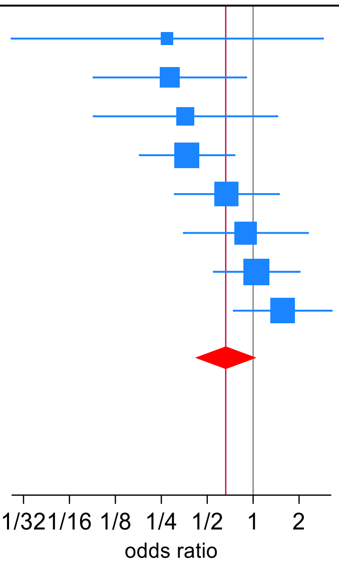

Figure s5 b&c Probiotic interventions. SROC's derived using 'metandi' (b) and 'metadta' (c).

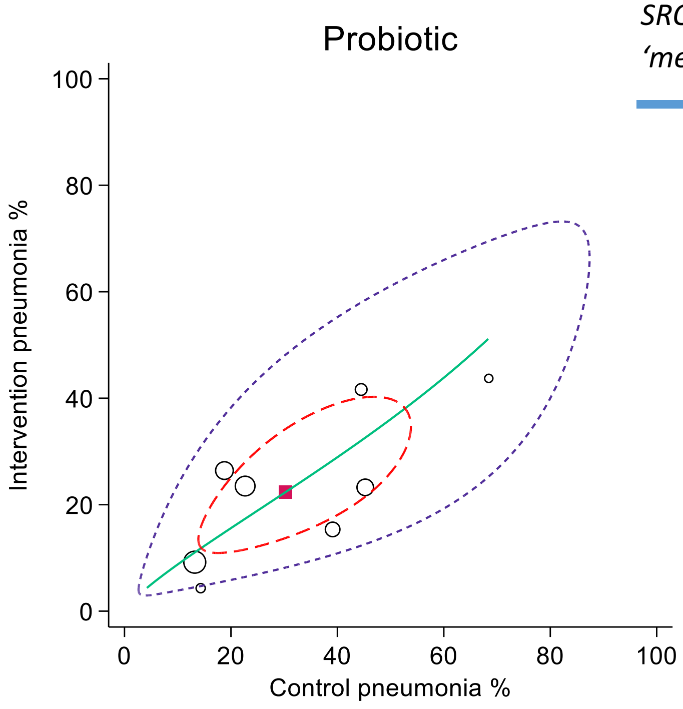

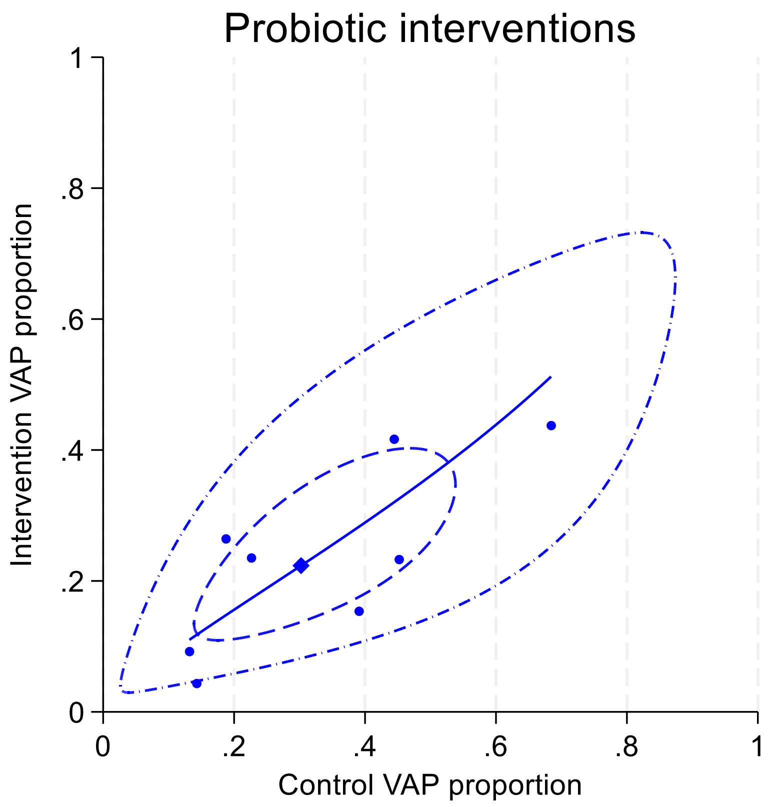

Figure s6a. Antiseptic and oral care interventions

Antiseptic & oral care interventions

Study	Treatment Yes	Treatment No	Control Yes	Control No	Odds ratio with 95% CI	Weight (%)
Fu , 117	7	33	37	3	0.02 [0.00, 0.07]	2
Tang, 141	5	25	25	5	0.04 [0.01, 0.16]	2.1
Yao, 144	4	24	14	11	0.13 [0.03, 0.49]	2.1
Seguin, 138	3	33	12	19	0.14 [0.04, 0.58]	2
Cabov, 112	1	16	6	17	0.18 [0.02, 1.64]	1.1
Feng, 132	8	57	29	39	0.19 [0.08, 0.46]	2.9
Fourrier'00, 115	5	25	14	14	0.20 [0.06, 0.67]	2.3
Mo, 134	15	90	47	58	0.21 [0.11, 0.40]	3.3
Hu, 133	4	21	10	12	0.23 [0.06, 0.89]	2.1
Nobahar, 135	5	29	13	21	0.28 [0.09, 0.90]	2.4
De Riso, 114	5	168	17	163	0.29 [0.10, 0.79]	2.6
Özçaka, 125	12	17	22	10	0.32 [0.11, 0.92]	2.6
Xu, 142	10	52	16	28	0.34 [0.13, 0.84]	2.8
Caruso, 131	14	116	31	101	0.39 [0.20, 0.78]	3.3
Tantipong, 128	5	53	10	42	0.40 [0.13, 1.25]	2.4
Grap, 118	7	14	10	8	0.40 [0.11, 1.47]	2.2
Xu, 142	11	47	16	28	0.41 [0.17, 1.01]	2.9
Chen, 113	16	44	28	32	0.42 [0.19, 0.89]	3.1
Feng, 132	18	53	29	39	0.46 [0.22, 0.94]	3.2
Stefanescu, 140	6	14	10	11	0.47 [0.13, 1.70]	2.2
Scannapieco, 126	14	83	12	37	0.52 [0.22, 1.23]	2.9
Koeman-Ch, 120	13	114	23	107	0.53 [0.26, 1.10]	3.2
Munro, 124	38	54	55	45	0.58 [0.32, 1.02]	3.5
Pobo, 136	15	59	18	55	0.78 [0.36, 1.69]	3.1
Prendergast, 137	8	30	10	30	0.80 [0.28, 2.31]	2.6
Zhao, 145	73	89	82	80	0.80 [0.52, 1.24]	3.7
Xu, 143	30	34	26	26	0.88 [0.42, 1.84]	3.2
Sebastian, 127	12	29	14	31	0.92 [0.36, 2.30]	2.8
Bellissimo-Rodrigues, 110	16	48	17	52	1.02 [0.46, 2.24]	3.1
Kusahara, 121	15	31	16	34	1.03 [0.44, 2.42]	2.9
Berry, 130	6	127	6	132	1.04 [0.33, 3.31]	2.4
Fourrier'05, 116	13	101	12	102	1.09 [0.48, 2.51]	3
Seguin, 139	24	54	20	52	1.16 [0.57, 2.34]	3.2
Meidani , 122	7	43	6	44	1.19 [0.37, 3.84]	2.4
Jacomo, 119	16	71	11	62	1.27 [0.55, 2.94]	3
Meinberg, 123	18	10	11	13	2.13 [0.70, 6.48]	2.5
Berry, 111	4	67	1	42	2.51 [0.27, 23.20]	1.1
Tuon, 129	4	4	2	6	3.00 [0.36, 24.92]	1.2
Berry, 111	4	29	1	42	5.79 [0.62, 54.52]	1.1
**Overall**					0.49 [0.37, 0.65]	

Heterogeneity: $\tau^2 = 0.50$, $I^2 = 69.74\%$, $H^2 = 3.30$

Test of $\theta_i = \theta_j$: Q(38) = 110.81, p = 0.00

Test of $\theta = 0$: z = -4.98, p = 0.00

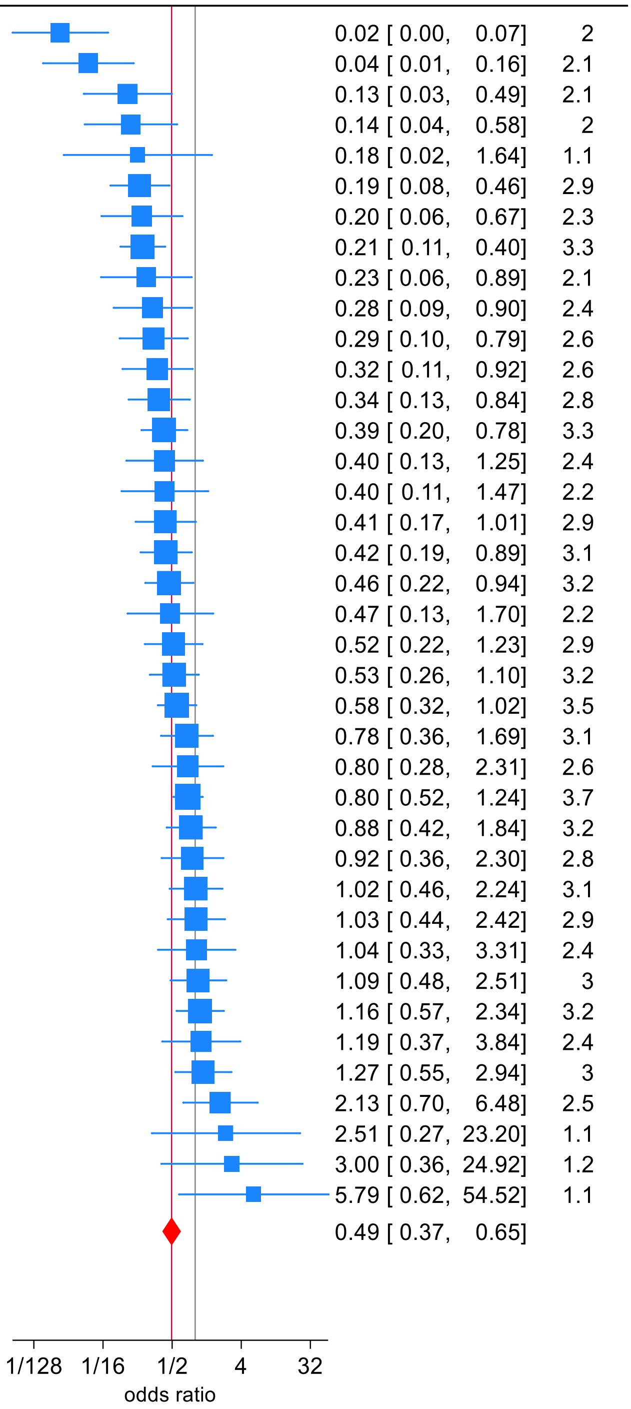

Figure s6 b&c Antiseptic interventions. SROC's derived using 'metandi' (b) and 'metadta' (c).

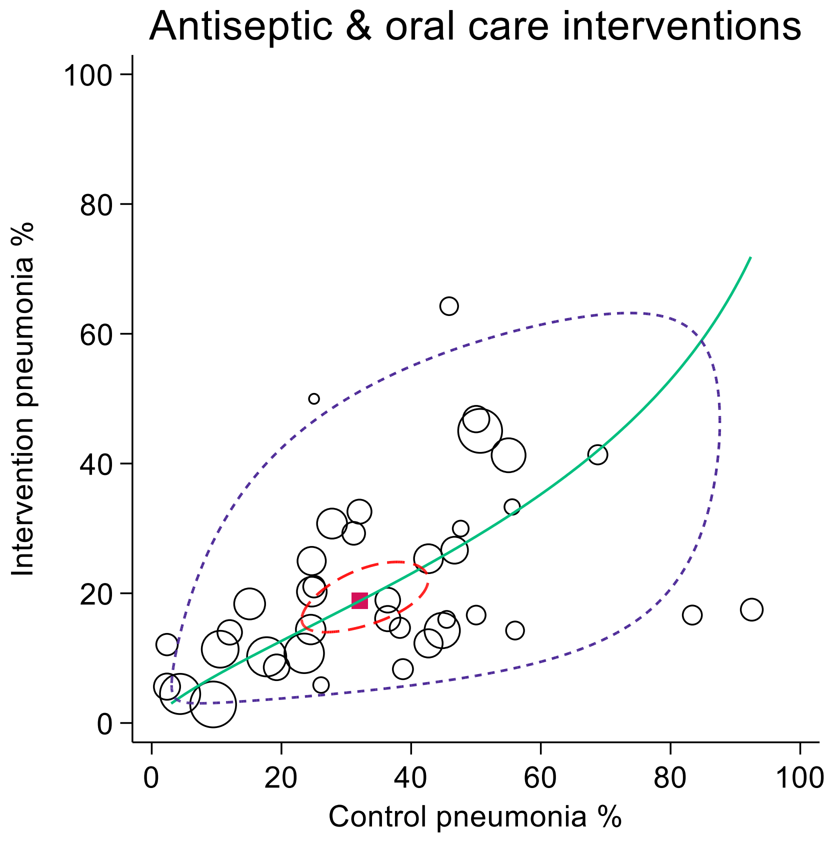

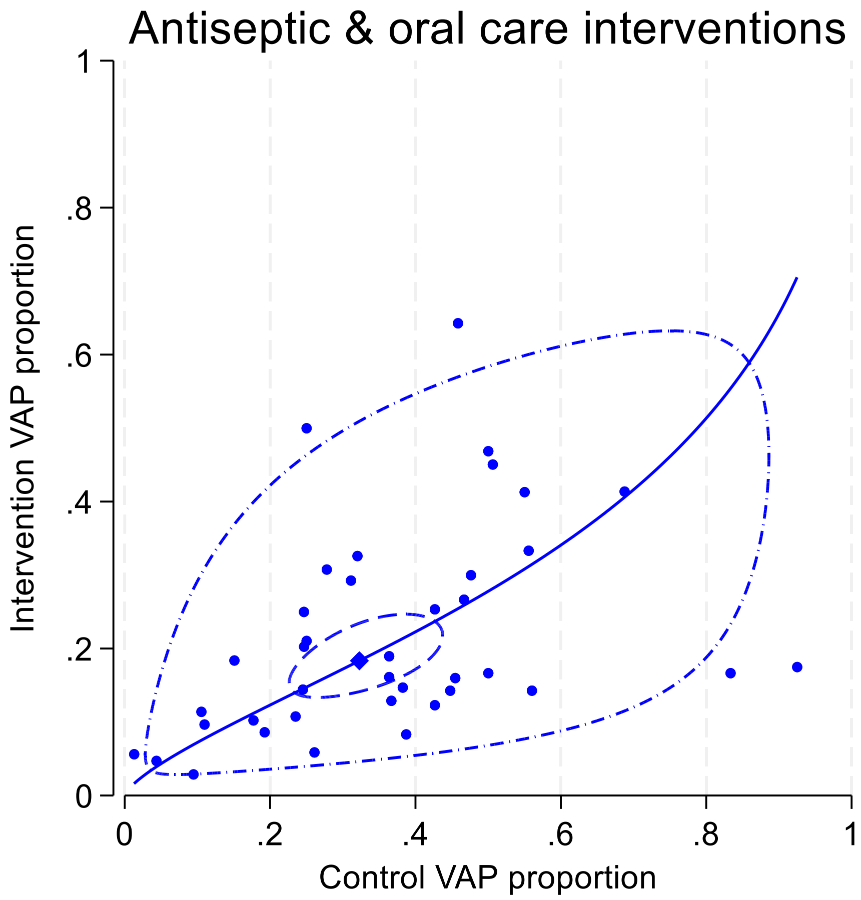

Figure s7a. Antiseptic & Antibiotic [antimicrobial] duplex interventions

Antimicrobial duplex interventions

Study	Treatment Yes	Treatment No	Control Yes	Control No	Odds ratio with 95% CI	Weight (%)
Stoutenbeek, 160	2	28	8	21	0.19 [0.04, 0.98]	3.7
Laggner, 158	1	32	4	30	0.23 [0.02, 2.22]	2.3
Dahiya, 147	2	33	7	28	0.24 [0.05, 1.26]	3.7
Long, 151	4	27	11	19	0.26 [0.07, 0.93]	5.3
Meidani , 153	6	44	15	35	0.32 [0.11, 0.91]	6.7
de Lacerda Vidal, 146	17	88	28	80	0.55 [0.28, 1.08]	9.7
Chaari, 155	10	21	6	7	0.56 [0.15, 2.09]	5.1
Ferrer, 156	7	32	10	31	0.68 [0.23, 2.01]	6.4
Panchabhai, 154	14	74	15	68	0.86 [0.39, 1.91]	8.6
Lorente'12, 152	21	196	24	195	0.87 [0.47, 1.62]	10
Hammond, 157	25	89	30	96	0.90 [0.49, 1.64]	10
Berry, 130	6	121	6	127	1.05 [0.33, 3.34]	6
Lingnau, 159	72	90	71	106	1.19 [0.78, 1.84]	12
Hanifi, 148	14	21	6	33	3.67 [1.22, 11.04]	6.3
Khaky, 150	9	29	1	36	11.17 [1.34, 93.37]	2.5
Irani, 149	6	29	0	35	15.64 [0.85, 289.36]	1.5
**Overall**					0.79 [0.54, 1.14]	

Heterogeneity: $\tau^2 = 0.25$, $I^2 = 53.38\%$, $H^2 = 2.14$

Test of $\theta_i = \theta_j$: Q(15) = 34.38, p = 0.00

Test of $\theta = 0$: z = -1.27, p = 0.21

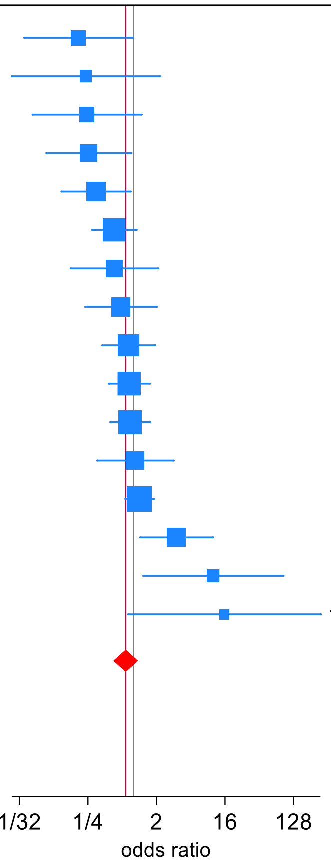

Figure s7b&c. Antiseptic & Antibiotic [antimicrobial] duplex interventions. SROC's derived using 'metandi' (b) and 'metadta' (c).

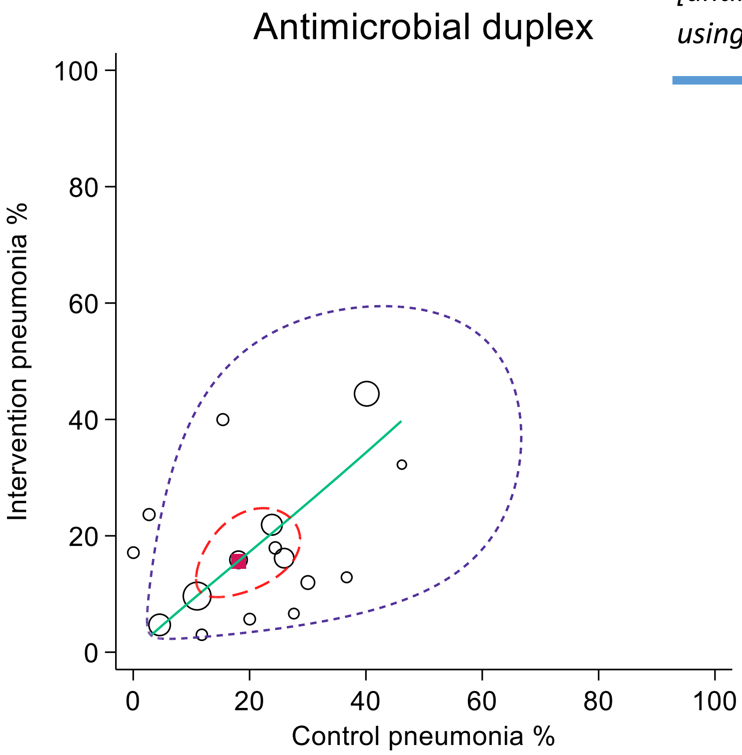

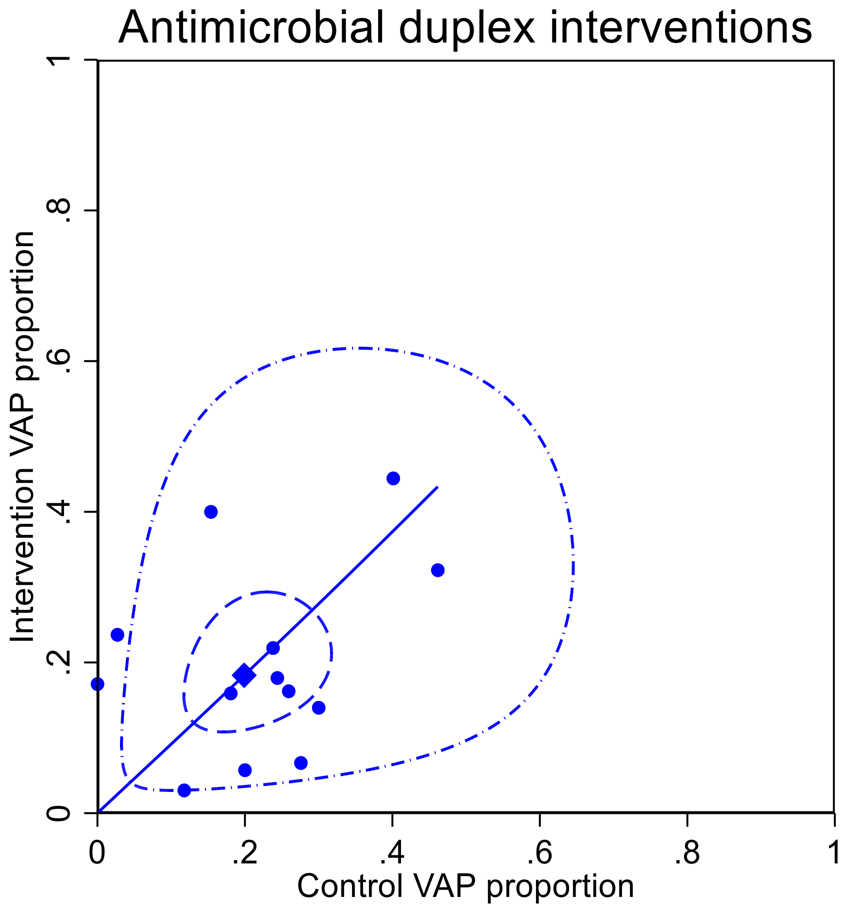

Figure s8a. Antibiotic interventions

Antibiotic interventions

Study	Treatment Yes	Treatment No	Control Yes	Control No	Odds ratio with 95% CI	Weight (%)
Aerdts, 162	1	17	29	10	0.02 [0.00, 0.17]	1.4
Rodríguez-Roldán, 188	0	13	11	6	0.02 [0.00, 0.41]	.85
Pugin, 186	4	21	24	3	0.02 [0.00, 0.12]	2.1
Kerver, 169	5	44	31	16	0.06 [0.02, 0.18]	3.2
Unertl, 189	1	18	9	11	0.07 [0.01, 0.61]	1.4
Jacobs, 168	0	36	4	39	0.12 [0.01, 2.31]	.86
Georges , 182	3	28	15	18	0.13 [0.03, 0.51]	2.6
Abele-Horn, 161	13	45	20	10	0.14 [0.05, 0.38]	3.5
Winter, 177	3	88	17	75	0.15 [0.04, 0.53]	2.8
Pneumatikos , 185	5	26	16	14	0.17 [0.05, 0.56]	2.9
Ulrich, 175	7	41	26	26	0.17 [0.06, 0.45]	3.5
Rocha, 172	7	40	25	29	0.20 [0.08, 0.53]	3.5
Palomar, 171	10	31	25	17	0.22 [0.09, 0.56]	3.6
Blair, 163	11	115	37	93	0.24 [0.12, 0.50]	4.2
Boland, 164	3	12	7	8	0.29 [0.06, 1.45]	2.1
Korinek, 184	20	43	37	23	0.29 [0.14, 0.61]	4.2
Cockerill, 165	4	71	12	63	0.30 [0.09, 0.96]	3
Quinio, 187	19	57	38	34	0.30 [0.15, 0.60]	4.3
Bergmans, 178	9	78	38	101	0.31 [0.14, 0.67]	4
Krueger, 170	91	174	149	113	0.40 [0.28, 0.56]	5.2
Sanchez-Garcia, 173	32	99	60	80	0.43 [0.26, 0.73]	4.8
Stoutenbeek, 174	62	139	100	100	0.45 [0.30, 0.67]	5.1
de la Cal, 166	18	35	26	28	0.55 [0.25, 1.21]	4.1
Finch, 167	4	16	7	17	0.61 [0.15, 2.48]	2.5
Koeman, 183	16	112	23	107	0.66 [0.33, 1.33]	4.3
Verwaest PTA, 176	31	169	40	145	0.66 [0.40, 1.12]	4.8
Brun-Buisson, 179	3	33	6	44	0.67 [0.16, 2.86]	2.4
Gastinne, 181	26	194	34	191	0.75 [0.44, 1.30]	4.7
Camus, 180	53	77	53	73	0.95 [0.58, 1.56]	4.9
Wiener, 190	8	22	8	23	1.05 [0.33, 3.27]	3.1
**Overall**					0.30 [0.22, 0.40]	

Heterogeneity: $\tau^2 = 0.41$, $I^2 = 71.90\%$, $H^2 = 3.56$

Test of $\theta_i = \theta_j$: Q(29) = 86.36, p = 0.00

Test of $\theta = 0$: z = -8.00, p = 0.00

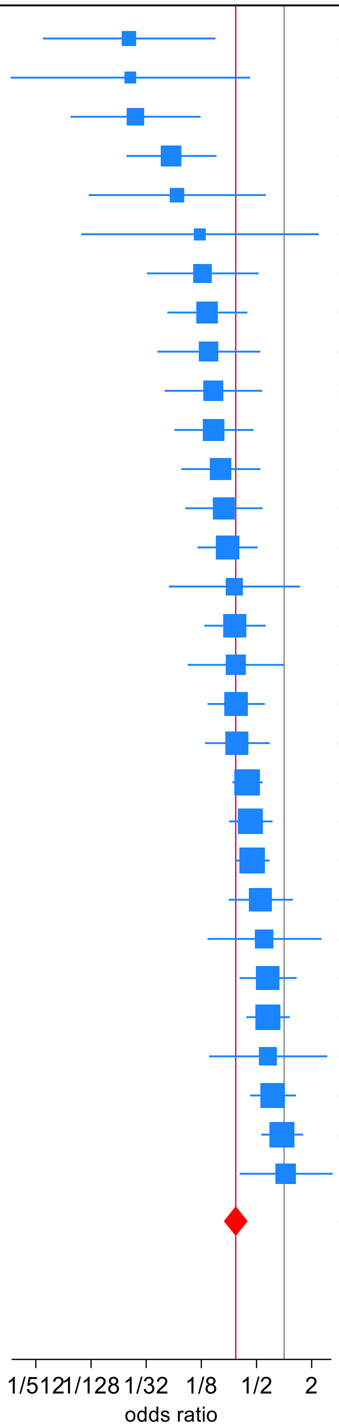

Figure s8 b&c. Antibiotic interventions. SROC's derived using 'metandi' (b) and 'metadta' (c).

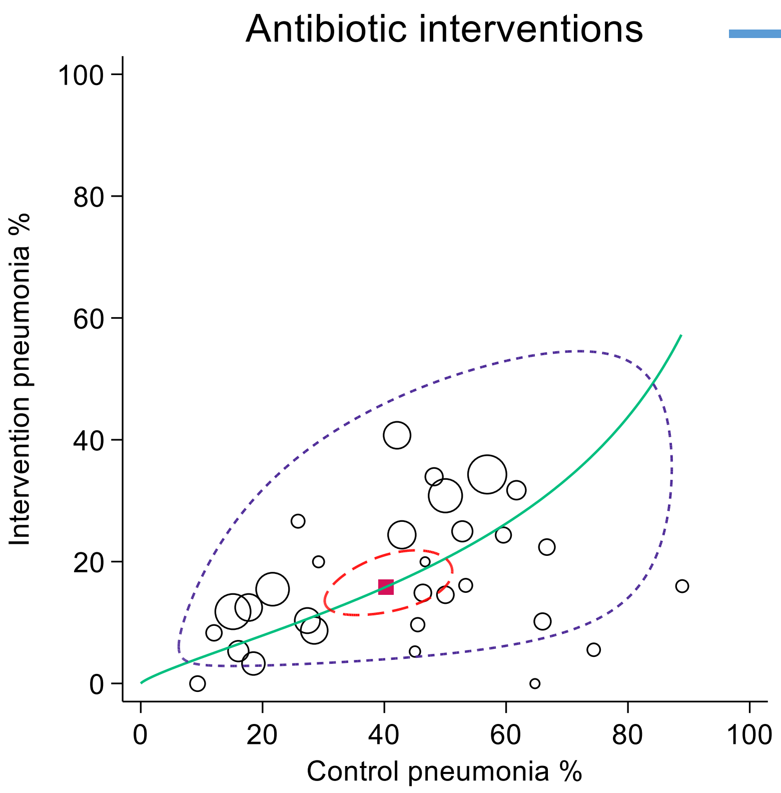

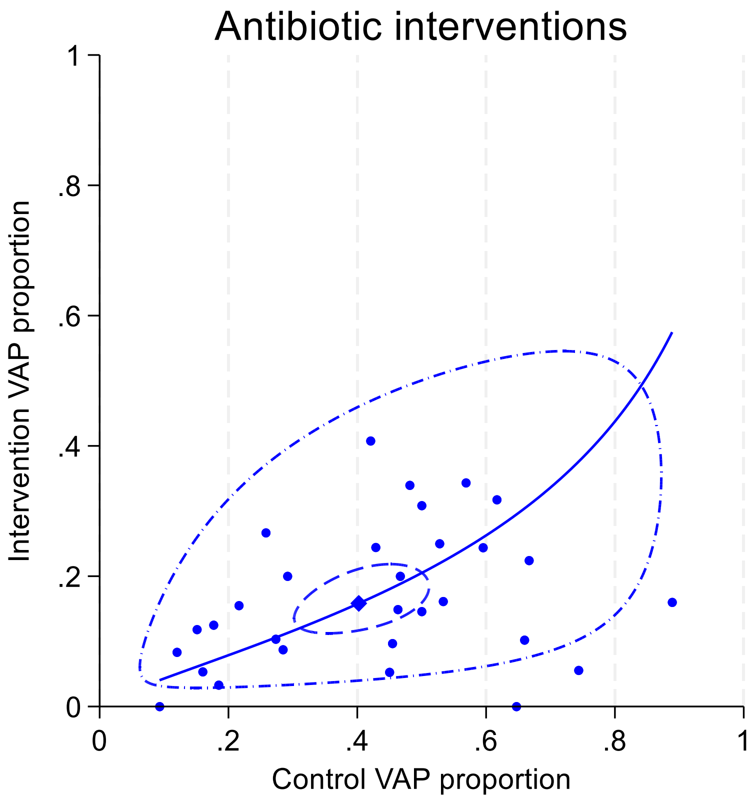

Figure s9 a & b SROC plot: Antimicrobial interventions (Control VAP < 40% [a] versus >40% [b]). SROC's derived using 'metandi'.

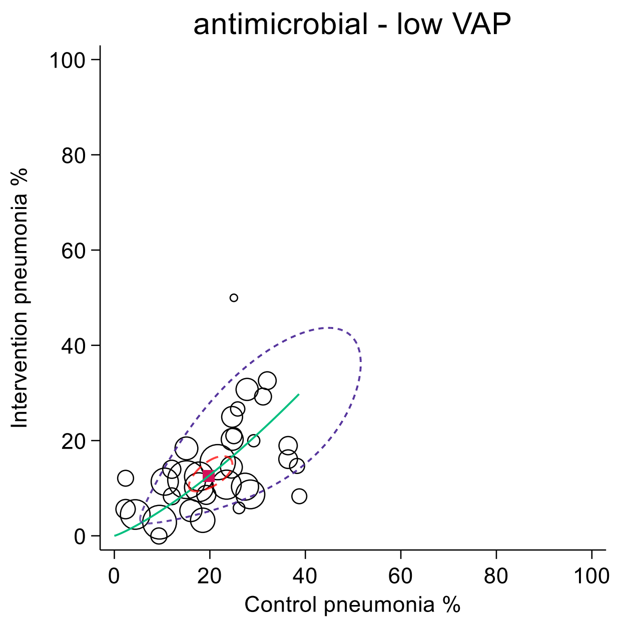

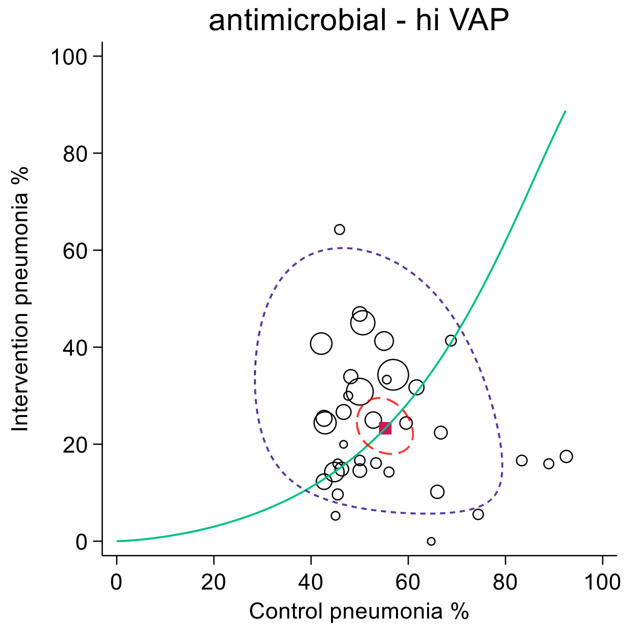

Figure s10 a, b & c Non-antimicrobial RCCT's with simulated positive uniform spillover

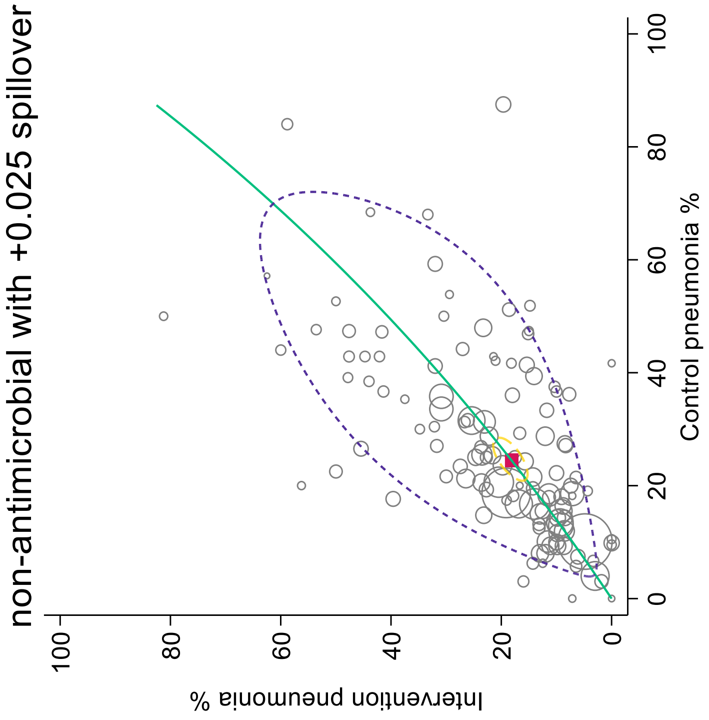

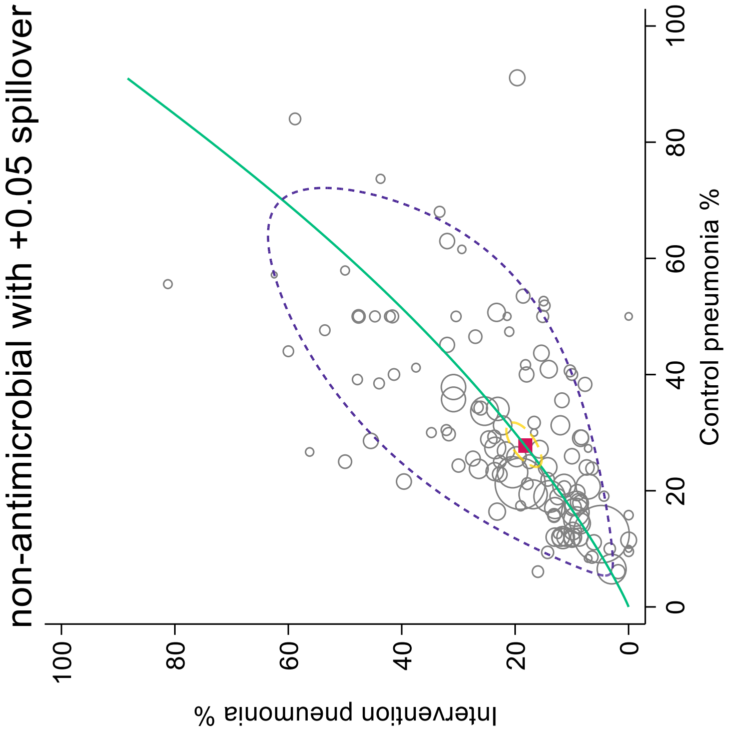

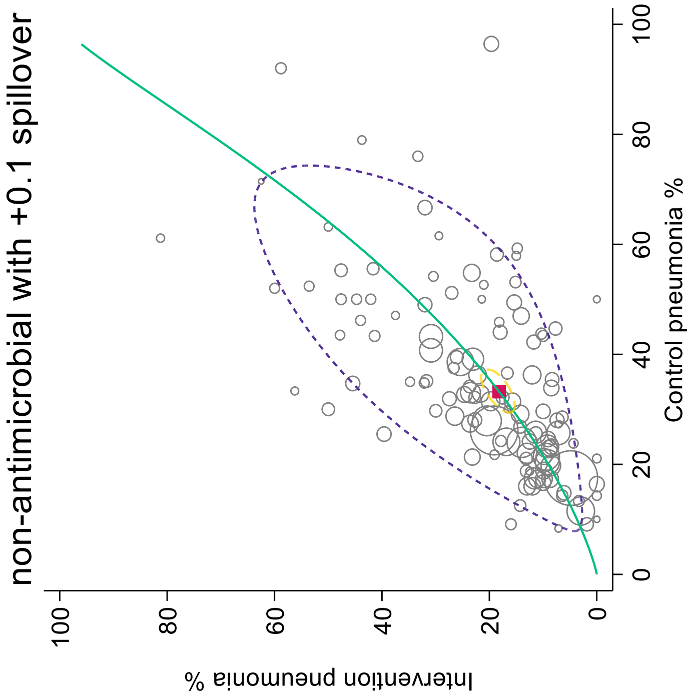

Figure s11 a, b & c Non-antimicrobial RCCT's with simulated positive partial spillover

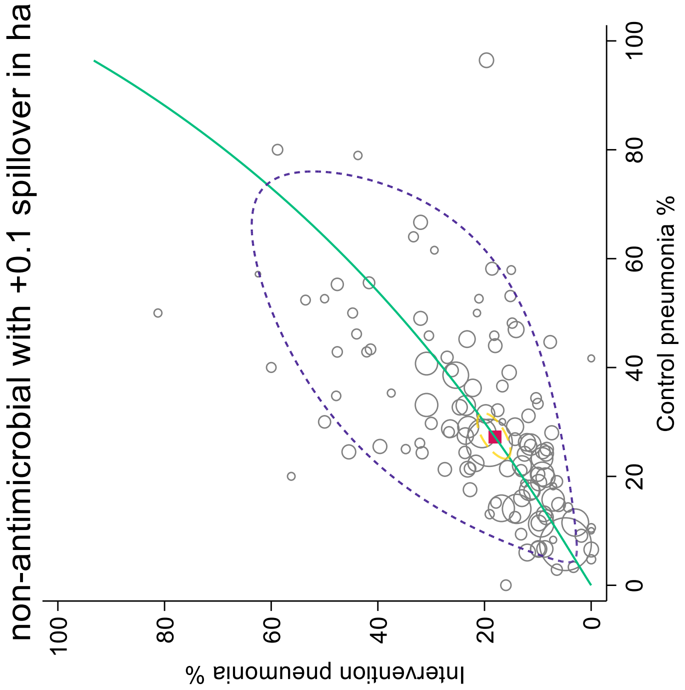

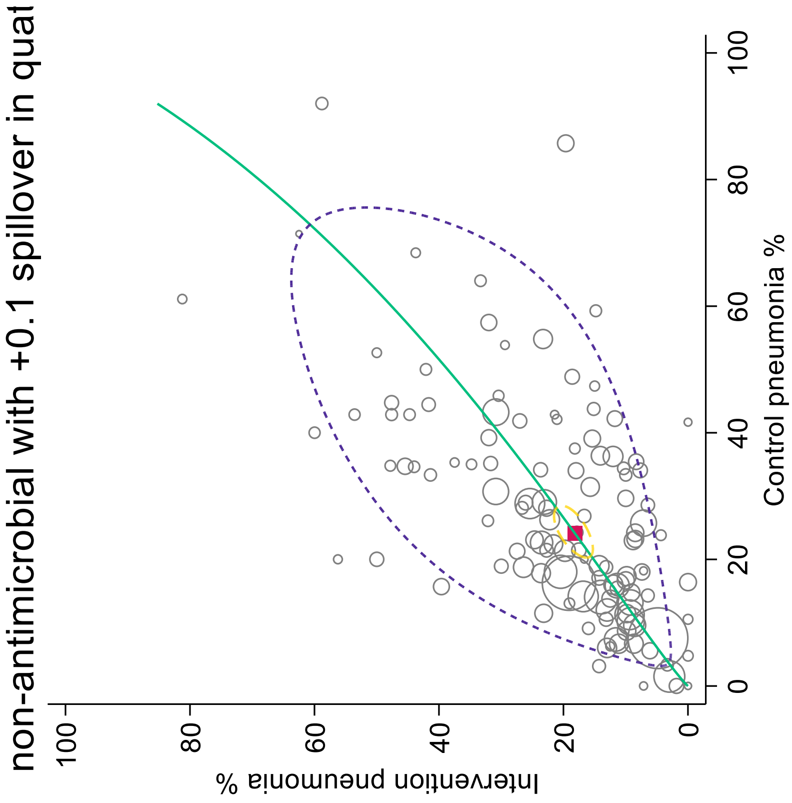
non-antimicrobial with +0.1 spillover in quat
Intervention pneumonia %
Control pneumonia %
0
20
40
60
80
100

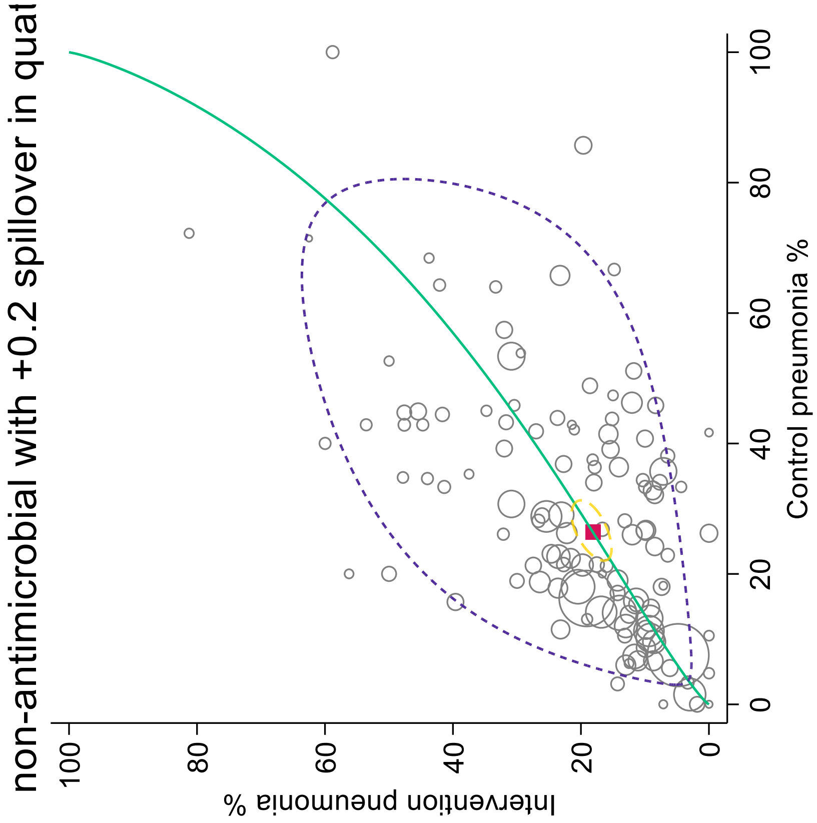
non-antimicrobial with +0.2 spillover in quat
Intervention pneumonia %
Control pneumonia %
0
20
40
60
80
100

Figure s12 a & b Non-antimicrobial RCCT's with simulated negative uniform spillover

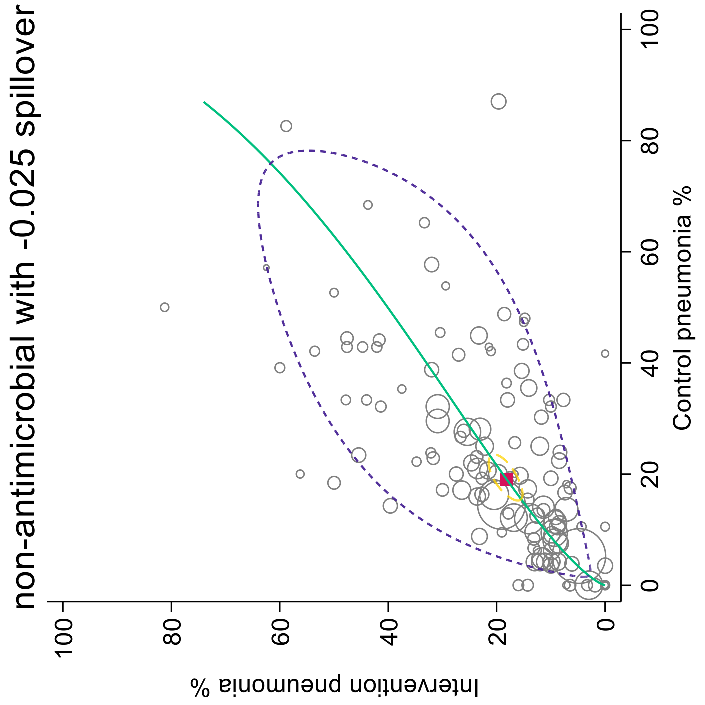

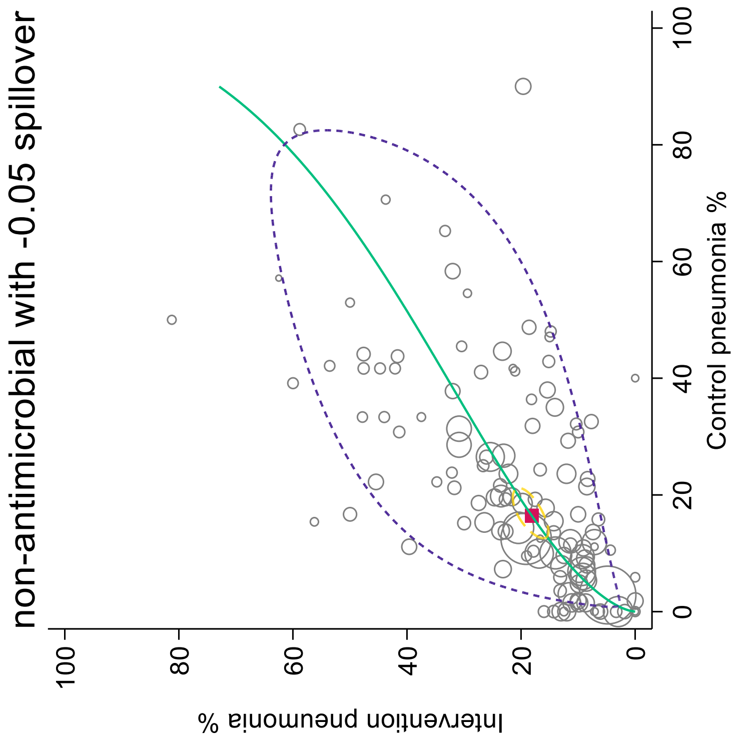

Supplement: Supplementary file 1 — Supplementary Material 1. [file 12874_2024_2296_MOESM1_ESM.pdf]
